# Gene-drive-capable mosquitoes suppress patient-derived malaria in Tanzania

**DOI:** 10.1038/s41586-025-09685-6

**Published:** 2025-12-10

**Authors:** Tibebu Habtewold, Dickson Wilson Lwetoijera, Astrid Hoermann, Rajabu Mashauri, Fatuma Matwewe, Rehema Mwanga, Prisca Kweyamba, Gilbert Maganga, Beatrice Philip Magani, Rachel Mtama, Moze Ally Mahonje, Mgeni Mohamed Tambwe, Felista Tarimo, Pratima R. Chennuri, Julia A. Cai, Giuseppe Del Corsano, Paolo Capriotti, Peter Sasse, Jason Moore, Douglas Hudson, Alphaxard Manjurano, Brian Tarimo, Dina Vlachou, Sarah Moore, Nikolai Windbichler, George K. Christophides

**Affiliations:** 1 Department of Life Sciences, Imperial College London, London, UK; 2 Environmental Health and Ecological Sciences, Ifakara Health Institute, Bagamoyo, Tanzania; 3 Department of Epidemiology and Public Health, Swiss Tropical and Public Health Institute, Allschwil, Switzerland; 4 University of Basel, Basel, Switzerland; 5 National Institute for Medical Research, Mwanza Centre, Mwanza, Tanzania; 6 Present Address: Liverpool School of Tropical Medicine, Liverpool, UK

**Keywords:** Genetic engineering, Pathogens

## Abstract

Gene drive technology presents a transformative approach to combatting malaria by introducing genetic modifications into wild mosquito populations to reduce their vectorial capacity. Although effective modifications have been developed, these efforts have been confined to laboratories in the global north. We previously demonstrated that modifying *Anopheles gambiae* to express two exogenous antimicrobial peptides inhibits the sporogonic development of laboratory-cultured *Plasmodium falciparum*, with models predicting substantial contributions to malaria elimination in Africa when integrated with gene drive^1–3^. However, the effectiveness of this modification against genetically diverse, naturally circulating parasite isolates remained unknown. To address this critical gap, we adapted our technology for an African context by establishing infrastructural and research capacity in Tanzania, enabling the engineering of local *A. gambiae* under containment. Here we report the generation of a transgenic strain equipped with non-autonomous gene drive capabilities that robustly inhibits genetically diverse *P. falciparum* isolates obtained from naturally infected children. These genetic modifications were efficiently inherited by progeny when supplemented with Cas9 endonuclease provided by another locally engineered strain. Our work brings gene drive technology a critical step closer to application, providing a locally tailored and powerful tool for malaria eradication through the targeted dissemination of beneficial genetic traits in wild mosquito populations.

## Abstract (web summary)

Engineering of local *Anopheles gambiae* under containment enables the generation of a transgenic strain equipped with non-autonomous gene drive capabilities that robustly inhibits genetically diverse *Plasmodium falciparum* isolates obtained from naturally infected children.

## Main

Malaria remains a major public health concern, with many African nations being far from meeting their malaria elimination targets^4,5^. Vector control methods including indoor residual spraying and long-lasting insecticide-treated bed nets have played a pivotal role in reducing malaria incidence, but the emergence of insecticide-resistant mosquitoes has impeded further progress^6^. In addition, Africa’s rapidly growing population and persistent malaria receptivity make these interventions increasingly unsustainable as standalone solutions. This highlights the urgent need for innovative, self-sustaining and cost-effective technologies to complement existing efforts in malaria elimination. Gene drive technology, which enables the biased inheritance of selected traits and can spread through populations at rates exceeding those predicted by Mendelian genetics, has emerged as a promising new paradigm^7,8^.

Gene drive can offer a transformative solution for malaria elimination by spreading genetic modifications that can either suppress mosquito populations or render them unable to transmit the disease^2,9,10^. Our work focuses on the latter approach known as mosquito population modification or replacement, whereby antiparasitic effectors introduced into the mosquito genome are spread to fixation within populations using a Cas9 endonuclease-based synthetic gene drive. In our design, the transmission-blocking effector and gene drive functions are separated into distinct genetic traits and strains^3,11–13^. This separation offers several advantages: it allows the development, testing and optimization of effector constructs in endemic settings independently of a full gene drive system; it facilitates rigorous risk assessment and community engagement before introducing self-propagating elements and it provides a safer, more modular pathway towards deployment^12^. Crucially, evaluating non-autonomous effector strains helps address elevated regulatory and containment requirements associated with autonomous gene drive systems.

Genetic modification of mosquitoes to reduce their vectorial capacity was first attempted more than two decades ago, and dozens of transgenic strains have been described in the literature to date^9,14–30^. However, no effector has ever been evaluated against parasites other than laboratory strains many of which were established in the early 1980s^31^. For this reason, their propensity to block the transmission of genetically diverse *Plasmodium* isolates now in circulation is unknown.

We previously demonstrated the efficacy of one such *A. gambiae* effector modification in inhibiting the NF54 strain of laboratory-cultured *P.* *falciparum*. This modification, termed MM-CP, involves two antimicrobial peptides, magainin 2 from the African clawed frog and melittin from the European honeybee^32^, integrated into and expressed from within the endogenous zinc carboxypeptidase A1 gene (*CP*)^33^. This minimal genetic modification that harbours no fluorescent markers interferes with oocyst development causing a significant delay in the release of infectious sporozoites. It also reduces the lifespan of homozygous female mosquitoes, further minimizing their potential to transmit malaria. Predictive models suggest that gene-drive-mediated population-wide propagation of MM-CP could disrupt disease transmission across various settings, offering promise for malaria elimination even in scenarios in which resistance to the effector or the drive eventually emerge. Here we adapted this technology for an African context to evaluate its ability to suppress *P. falciparum* parasites naturally circulating among humans.

**Fig. 1:**
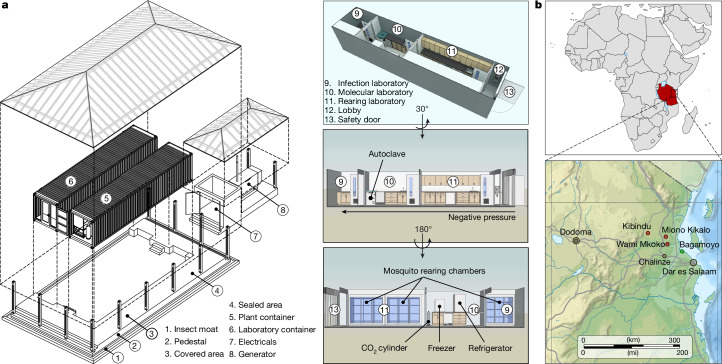
Infrastructure capacity building and malaria surveillance sites in northeastern Tanzania. **a**, Integrated MPL/CL3 facility. Architectural design plans for the (left) and a detailed view of the integrated laboratory and insectary container unit (right) are shown. The laboratory comprises a lobby, an incubator room for mosquito husbandry, a molecular biology laboratory and a dedicated space for *P. falciparum* DMFAs and housing of infected mosquitoes. The second container unit houses systems that regulate and maintain optimal environmental conditions, including a negative pressure system for biosecurity, water purification and waste treatment. An external electricity generator supports these operations. **b**, Field sites for parasitological surveys and gametocyte carrier recruitment. Locations of villages in the Pwani region where parasitological surveys were conducted in children are shown in relation to the IHI Bagamoyo campus (housing the MPL/CL3 facility), the capital Dodoma, the major port city Dar es Salaam and the town of Chalinze, where meteorological data were recorded. The map is modified to highlight sites mentioned in the paper. Tanzania road map in **b** adapted from OnTheWorldMap.com (https://ontheworldmap.com/tanzania/tanzania-road-map.html).

The implementation of gene drive technologies in malaria-endemic regions faces substantial challenges, including limited access to appropriate containment infrastructure, regulatory uncertainty, insufficient local capacity for genetic engineering and biosafety, and the imperative for community trust and public transparency. To enable our work, we developed an integrated Modular Portable Laboratory and Containment Level 3 (MPL/CL3) insectary facility, specifically designed for generating, housing and studying genetically modified mosquitoes within an African context (Fig. 1a). The MPL/CL3 was designed to address some of these constraints by offering a high-security and standardized facility tailored to local environmental and regulatory conditions. It incorporates climate and illumination control systems, rearing chambers, microbiologically safety cabinets, water management and waste disposal systems, an autoclaving unit and a fully equipped laboratory. The facility was constructed within two intermodal shipping containers in Spain and transported and installed at the Bagamoyo campus of the Ifakara Health Institute (IHI) in Tanzania (Fig. 1b). By embedding cutting-edge vector biology capacity within endemic settings, the MPL/CL3 supported local research leadership, regulatory readiness and public engagement, laying essential groundwork for responsible development and evaluation of gene drive technologies. Detailed specifications and technical plans are presented in the Methods and Supplementary Note. All protocols involving the generation and study of transgenic mosquitoes were reviewed and approved by the relevant institutional and national regulatory authorities in Tanzania.

The first *A. gambiae* transgenic line developed onsite within the MPL/CL3, named zpg-CC, was designed to streamline all transgenesis processes by expressing both Cre recombinase and Cas9 endonuclease under the control of the zero-population growth (*zpg*) gene promoter. This dual helper strain enables the efficient removal of sequences such as transgenesis markers flanked by loxP sites and establishment of transgene homozygosity through homing. The initial development and characterization of the zpg-CC line were conducted at Imperial, before the line was recreated in Tanzania.

**Extended Data Fig. 1:**
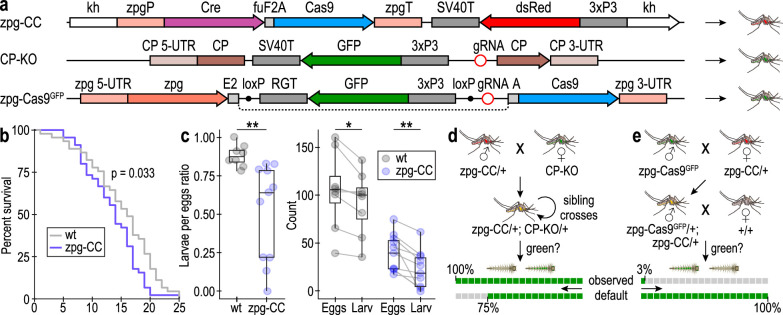
Design and characterisation of the *An. gambiae* zpg-CC transgenic line. **a**, Schematic representation of the molecular designs of transgenic lines used in this study (not to scale). zpg-CC: Inserted in the kh locus and contains a 3xP3-DsRed-SV40T marker, Cre recombinase and Cas9 under zpg promoter (zpgP)/terminator(zpgT) control, with Cre and Cas9 separated by a furin cleavage site and F2A ribosome skipping peptide (fuF2A). CP-KO: Includes a 3xP3-GFP-SV40T cassette and a gRNA under a U6 promoter (open red circle) inserted within the CP gene. zpg-Cas9^GFP^: Features Cas9 at the C-terminus of the zpg gene, separated by an E2A peptide split by the *An. gambiae gambicin* intron containing a 3xP3-GFP-RGT cassette flanked by loxP sites, and a U6-driven gRNA. RGT corresponds to the sequence of the rabbit global terminator cloned from the 3′UTR of the pTriEx7 expression system plasmid (Novagen). **b**, Kaplan-Meier survival analysis of zpg-CC and wt female mosquitoes (n = 15) maintained on sugar (Mantel-Cox/log-rank test) conducted in triplicates. **c**, Fecundity and fertility of individual zpg-CC females (n = 8) compared to wt controls (n = 11). Box plots show median (line), interquartile range (box), and full data range (whiskers); outliers are plotted individually. Statistical analysis was performed with two-sided Wilcoxon signed-rank tests. Left: wt vs zpg-CC, **p = 0.0017 (**). Right: wt, p = 0.022 (*); zpg-CC, p = 0.0038 (**). **d**, Mating scheme for testing the ability of zpg-CC mosquitoes to induce homing of the CP-KO transgenic cassette within the CP locus. The bar chart shows the observed and default (if Cas9 was not functional) percentage of green-fluorescent larvae. **e**, Mating scheme assessing the capacity of zpg-CC mosquitoes to cause Cre-mediated excision of the GFP expressing cassette in zpg-Cas9^GFP^ mosquitoes. The bar chart shows the observed and default (if Cre was not functional and assuming 100% homing catalysed by a trans source of Cas9 as observed in panel 2d) percentage of green-fluorescent larvae.

The zpg-CC construct includes a dominant DsRed transgenesis marker, integrated into the *kynurenine hydroxylase* (kh) gene locus (Extended Data Fig. 1a). Disruption of both copies of the gene results in white-eyed mosquitoes, serving as a recessive phenotypic marker. Although the zpg-CC helper line was robust and fertile, it showed reduced overall fitness, probably due to the disruption of the kh locus and/or the effects of germline-specific or leaky expression of both Cre and Cas9. Compared with wild-type (wt) females, sugar-fed transgenic homozygous zpg-CC females showed a small decline in survival over time (Extended Data Fig. 1b), consistent with previous observations in other mosquitoes^34,35^. They also laid significantly fewer eggs after blood feeding, with a lower proportion hatching, indicating a reduction in reproductive fitness (Extended Data Fig. 1c).

To assess the efficiency of the zpg-CC helper line in inducing homing when combined with a non-autonomously driveable transgene expressing guide RNA (gRNA), we crossed heterozygous zpg-CC males with females of a previously generated *CP* knockout (CP-KO) line that harbour a green fluorescent protein (GFP) expression cassette and a gRNA expression module inserted within and targeting the *CP* gene^36^ (Extended Data Fig. 1a). Heterozygous offspring expressing both GFP and DsRed were sib-mated, and the resulting larvae were screened for green fluorescence. All 623 larvae screened were GFP positive, compared with the 75% expected from a Mendelian intercross of hemizygotes. This indicates 100% Cas9-mediated homing, induced by Cas9 provided by the zpg-CC helper line (Extended Data Fig. 1d).

Next, we assessed the capacity of the zpg-CC helper line to excise a loxP-flanked GFP expression cassette through the expression of Cre recombinase. As a tester line we used the zpg-Cas9^GFP^ strain, in which a Cas9 coding sequence was inserted within the *zpg* gene to encode Cas9 linked to the zpg C terminus through an E2A ribosome-skipping peptide sequence. An intron harbouring the excisable GFP expression cassette flanked by loxP sites and a gRNA module is located within the E2A sequence (Extended Data Fig. 1a). We crossed zpg-Cas9^GFP^ males with heterozygous zpg-CC females, selecting GFP and DsRed positive males for subsequent crosses with wt females (Extended Data Fig. 1e). Among 417 offspring larvae, only 13 showed green fluorescence, indicating efficient Cre-mediated excision of the GFP cassette (97%).

**Fig. 2:**
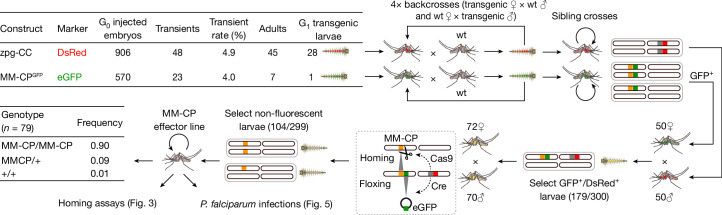
Schematic representation of the strategy for the generation of a markerless, homozygous MM-CP line. This approach involved a series of breeding and selection steps, detailed as follows in a clockwise progression. Top left, tabular summary of the processes for generating the zpg-CC and MM-CP^GFP^ transgenic lines. Top middle, each transgenic line was outcrossed with the wt Ifakara strain to enhance line vigour. Top right, strains were maintained through sibling mass crossing to preserve genetic stability. Bottom right, MM-CP^GFP^ females were crossed with zpg-CC males, and double-positive individuals (GFP and DsRed) were selected and mass-bred. Bottom middle, this process enabled Cre-mediated removal of the GFP expression cassette (floxing) and Cas9-driven homozygosity of the MM-CP transgene (homing). Bottom left, non-fluorescent individuals were selected for pupal case genotyping to identify homozygous MM-CP individuals. These homozygous mosquitoes were then mass-bred to establish the MM-CP line, subsequently used in homing and *P. falciparum* DMFA infection assays. eGFP, enhanced GFP.

These experiments confirmed efficient Cas9 and Cre expression by the zpg-CC helper strain. We therefore recreated the zpg-CC line in Tanzania, by microinjecting embryos of the *A. gambiae* Ifakara strain^37^ with the zpg-CC plasmid together with independent Cas9 and gRNA helper plasmids. G_0_ larvae showing transient fluorescence were allowed to mature into adults that were then crossed with wt mosquitoes in separate male and female crosses (Fig. 2). These crosses produced 28 G_1_ transgenic larvae expressing DsRed in the nervous tissue, of which 25 (16 males, 9 females) reached adulthood. After four rounds of backcrossing with wt mosquitoes, a pure and stable colony was established by continuously selecting larvae showing DsRed fluorescence and kh locus disruption, indicated by the absence of eye pigments. Despite Cas9 expression, these mosquitoes cannot autonomously propagate their modification through gene drive, as they lack genomic integration of a gRNA gene.

Next, we generated the MM-CP line by microinjecting *A. gambiae* wt Ifakara embryos with the MM^GFP^-CP plasmid, containing a gRNA for integration into the *CP* locus and a Cas9 source^38^. This resulted in a single G_1_ transgenic male expressing GFP in the eyes and ganglia, which was then backcrossed with wt mosquitoes for four generations to establish a stable MM^GFP^-CP precursor line (Fig. 2). To achieve transgene homozygosity and remove the GFP marker cassette, MM^GFP^-CP females were crossed with zpg-CC males and double fluorescent progeny were sib-mated. The resulting colony carried the antimalarial MM-CP transgene as a minimal, markerless modification. Molecular genotyping confirmed that most mosquitoes in the colony were homozygous for the transgene (Fig. 2).

**Fig. 3:**
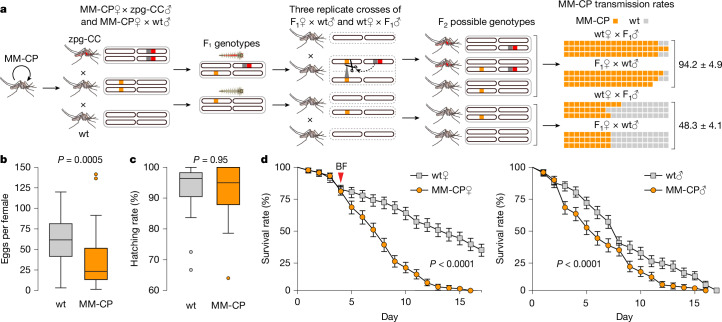
Transmission efficiency and life history traits of MM-CP Ifakara mosquitoes. **a**, Schematic of the non-autonomous homing assay used to evaluate transmission efficiency of the MM-CP transgene. Homozygous MM-CP females were crossed with either zpg-CC males (Cas9 source) or wt Ifakara males (control). F_1_ progeny were sexed and reciprocally crossed with wt Ifakara mosquitoes. Twenty F_2_ progeny per cross (in triplicate) were genotyped by PCR to detect the MM-CP transgene. The bar graph (far right) shows inheritance rates, with significantly higher MM-CP transmission in zpg-CC crosses, confirming efficient non-autonomous homing. Each row represents a biological replicate, and each box denotes one mosquito (5% rate). Note that only 17 mosquitoes were genotyped in 1 replicate. **b**, Fecundity of MM-CP (*n* = 23 and 25) and wt (*n *= 25 and 24) females, measured as the number of eggs laid per mosquito in two independent biological replicates. MM-CP females showed significantly reduced egg output compared with wt controls (*P* = 0.0005, two-sided Mann–Whitney *U*-test). Boxplots show median, interquartile range (25th to 75th percentiles) and full data range (whiskers) for each group; dots outside boxplots are outliers. Source data are provided in Source Data Sheet 1. **c**, Mean fertility of female mosquitoes used for the fecundity assays (**b**), measured as hatching rate (% of eggs developing into larvae), did not differ significantly between lines (*P* = 0.95, two-sided Mann–Whitney *U*-test). Error bars show the range of fertility rates across the two replicates. Source data are provided in Source Data Sheet 1. **d**, Survival curves postemergence. Left, females post-bloodmeal (BF); right, sugar-fed males. MM-CP females showed markedly reduced survival after blood feeding (*P* < 0.0001, log-rank test); males also showed reduced survival under sugar-only conditions (*P* < 0.0001), although to a lesser extent. Data points represent the mean of two independent biological replicates (MM-CP, *n* = 378 and 242; wt, *n* = 368 and 350), each comprising three cages of mosquitoes reared from separate aquatic trays. Error bars indicate the range between replicates. Source data are provided in Source Data Sheet 2.

To quantify MM-CP inheritance rates in the presence of Cas9, we crossed MM-CP females with zpg-CC males (which express Cas9) and vice versa (Fig. 3a). Equivalent crosses with wt mosquitoes served as controls. The resulting F_1_ zpg-CC;MM-CP and +;MM-CP heterozygotes from these experimental crosses were then separately crossed with wt mosquitoes, with male and female G_1_ individuals crossed independently. We conducted three replicates for each cross and genotyped 20 F_2_ progeny per replicate to assess the presence of the MM-CP transgene in a heterozygous state (Extended Data Table 1). Both male and female MM-CP;zpg-CC crosses with wt mosquitoes showed high inheritance rates of the MM-CP transgene to F_2_ progeny, averaging 94.2 ± 4.9%, compared with the control crosses (MM-CP females crossed to wt males), which showed near-Mendelian segregation at 48.3 ± 4.1% (Fig. 3a). These results indicate high rates of non-autonomous gene drive of the MM-CP transgene when combined with germline Cas9 expression.

MM-CP mosquitoes originally generated in a mixed KIL/G3 genetic background showed reduced fitness, including lower fecundity and decreased survival, particularly in females^38^. KIL and G3 are two genetically distinct *A. gambiae* laboratory strains colonized from northern Tanzania and The Gambia in the 1970s, respectively. Life history assays with the new MM-CP line in the *A. gambiae* Ifakara background yielded similar results, confirming that these phenotypes are consistent across genetic backgrounds, an important consideration for gene drive deployment. Specifically, MM-CP females laid significantly fewer eggs than controls (Fig. 3b), although hatching rates were comparable (Fig. 3c). Survival was reduced in both sexes, with the most pronounced effect observed in females following a bloodmeal (Fig. 3d). Although some survival effects may reflect inbreeding from the transgenesis process and are unlikely to persist under gene drive conditions involving continuous outcrossing, the sharp post-bloodmeal decline in female survival is probably driven by strong antimicrobial peptide expression at the bloodmeal-inducible *CP* locus or perturbations of CP expression due to the antimicrobial peptide integration. This phenotype is modelled to enhance the efficacy of the intervention by reducing the likelihood that infected females survive long enough to transmit the disease^38^. Despite these fitness costs, multi-generational cage experiments have demonstrated that MM-CP can still be driven efficiently to near-fixation when combined with a self-propagating Cas9 source, supporting the robustness of MM-CP under gene drive rconditions^39^.

**Fig. 4:**
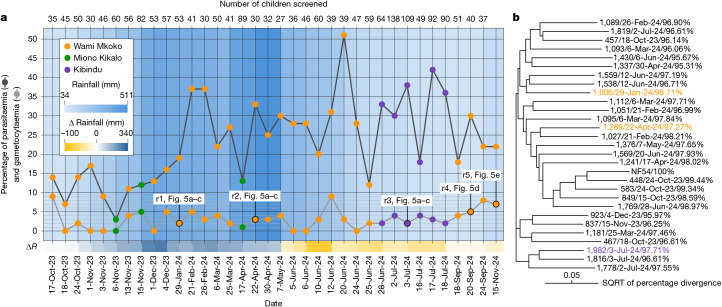
*P. falciparum* epidemiology and phylogenetic analysis. **a**, Epidemiological data on *P. falciparum* parasitaemia and gametocytaemia among children in three villages within the Pwani region. The bottom *x* axis indicates screening dates, the top *x* axis indicates the number of children screened each day, and the *y* axis shows the percentage of parasitaemic and percentage of gametocytaemic children among the total number of children screened on each date. The total number of children screened per date is shown above the graph. Dates when gametocytaemic blood samples (one per date) were used for successful mosquito infections are indicated, with corresponding results reported in referenced figure panels. The blue gradient in the background represents the 60-day cumulative rainfall (mm) before each survey, recorded at the Chalinze meteorological station, and the gradient below the graph (yellow to blue) shows the difference in rainfall (mm) compared with the same period 1 year earlier. Screening dates when gametocytaemic blood was collected for the mosquito infection replicates (r1–5) presented in Fig. 5 are indicated. Source data are provided in Source Data Sheet 3. **b**, Phylogenetic tree of *P. falciparum* isolates obtained from 1–2 gametocytaemic children on most screening days. Consensus sequences of the *CSP*, *AMA1*, *SERA2* and *TRAP* genes were concatenated and aligned to assess genetic relatedness. Each tip label shows the sample ID, collection date and percentage sequence identity to the *P. falciparum* NF54 reference genome. Coloured labels correspond to isolates used for mosquito infection experiments and are matched to their respective village of origin as shown in **a**. The scale bar represents sequence divergence, expressed as the square root of percentage sequence divergence. Raw sequencing data are available under BioProject accession PRJNA1299763 (NCBI SRA).

Next, we proceeded to assess the efficacy of the locally developed MM-CP strain in inhibiting parasites circulating among infected children. We conducted surveys in primary school pupils in Wami Mkoko and Miono Kikalo villages to determine the levels of parasitaemia and gametocytaemia (Fig. 2b). These surveys were later expanded to include children aged 6–14 in Kibindu village. Malaria infection was determined using rapid diagnostic testing (RDT), with parasites quantified through thick blood smear microscopy. Written informed consents were obtained from parents or guardians, and oral assent was secured from children. These activities were conducted alongside structured community engagement to promote transparency, address concerns and foster public trust. The results indicated year-round malaria transmission, with parasitaemia and gametocytaemia present in roughly 25–30% and 2–5% of screened children, respectively (Fig. 4a). The high malaria prevalence throughout the survey period may be linked to the 2023–2024 El Niño-Southern Oscillation that occurred between July 2023 and April 2024 and was associated with significantly more rainfall in this part of the country^40^. To ensure that a diverse array of parasite genotypes were circulating in these three villages, we sequenced four genes (*CSP*, *AMA1*, *SERA2* and *TRAP*) known to provide a robust measure of *P. falciparum* diversity^41^. The results confirmed that our sampling strategy captured a representative diversity of genotypes in these communities (Fig. 4b).

Children with high gametocyte densities were invited to provide blood samples for mosquito direct membrane feeding assays (DMFAs). Previous studies have shown that infection outcomes from DMFAs correlate closely with those from direct skin feeding, supporting their biological relevance^42,43^. Of numerous DMFAs conducted, five produced significant oocyst numbers and were processed further. The first three infections were used to assess oocyst counts and sizes at 9 days postfeeding, whereas the last two replicates served to quantify sporozoite loads in mosquito midgut and head and/or thorax tissues using real-time quantitative PCR (qPCR) at 13–15 days postfeeding. The parasite genotypic analysis confirmed that isolates used for the first three experiments diverge from the NF54 reference genome and from each other (Fig. 4b). Isolates used in the last two infection experiments were not sequenced.

**Fig. 5:**
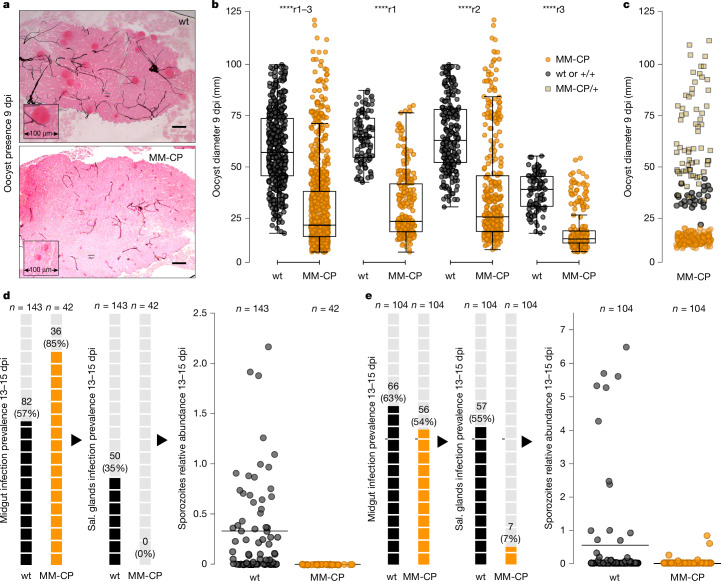
Effect of MM-CP transgenic mosquitoes on *P. falciparum* oocyst growth and sporozoite output. **a**, Representative images of mercurochrome-stained midguts from wt and MM-CP transgenic mosquito lines at 9 days post-infectious bloodmeal (dpi), showing marked differences in oocyst size. Insets show magnified regions of oocyst clusters. **b**, Quantification of oocyst diameters in midguts from wt (*n* = 19, *n* = 37 and *n* = 17, respectively) and MM-CP (*n *= 25, *n* = 29 and *n* = 21, respectively) mosquitoes from three independent infections experiments using *P. falciparum* gametocytes from infected children. The leftmost plot shows pooled data from all replicates (r1–3) and the remaining plots show data from each replicate individually. Boxplots show median, interquartile range and full data range (whiskers) for each group; dots outside the boxplots are outliers. Significance was tested using the Kruskal–Wallis *H-*test (****P < 0.0001; effect size *η*^2^ = 0.355). Note that some variability in oocyst size is also visible in wt midguts, reflecting natural variation commonly observed in wt infections. Source data are provided in Source Data Sheet 4. **c**, Oocyst diameters in midguts of the MM-CP line classified by genotype: homozygous MM-CP (orange circles), heterozygous MM-CP/+ (yellow squares) or wt^+/+^ (grey circles). Note that all oocysts in wt mosquitoes originate from only two midguts, cautioning against any interpretations of size differences relative to heterozygous mosquitoes. Source data are provided in Source Data Sheet 4. **d**, Sporozoite data in wt and MM-CP mosquitoes from the fourth infection replicate (r4) assayed at 13–15 dpi. Left, prevalence of midgut sporozoites. Middle, prevalence of sporozoites in head and/or thorax tissues (used as salivary (sal.) glands proxy). Each shaded box represents 5% prevalence. Total numbers assayed and positives are shown. Right, dot plot showing relative sporozoite abundance in head and/or thorax samples. Source data are provided in Source Data Sheet 5. **e**, Same analyses as in **d**, shown for the fifth replicate (r5). Note that between the third and fourth replicates, the colony was further cleaned using pupal case genotyping to enrich for MM-CP homozygotes. Source data are provided in Source Data Sheet 5. Scale bars, 100 µm.

Microscopy showed that most MM-CP mosquito midguts contained notably smaller oocysts (Fig. 5a), consistent with what was previously observed in KIL/G3 MM-CP mosquitoes infected with laboratory *P. falciparum*^38^. Quantitative measurements indicated a median oocyst diameter of 22.2 µm in MM-CP compared with 57.3 µm in wt midguts (Fig. 5b). However, some MM-CP mosquitoes also contained larger oocysts, similar in size to those in wt mosquitoes. Molecular genotyping of mosquito carcasses revealed that these midguts derived from heterozygous MM-CP or non-transgenic mosquitoes present in the MM-CP colony (Fig. 5c).

To improve the consistency of phenotypic analyses, we enriched the MM-CP transgene in the colony by implementing a pupal case genotyping strategy to identify and select homozygous individuals, thereby eliminating wt alleles at the *CP* locus and increasing the proportion of MM-CP homozygotes. In the fourth and fifth replicates, mosquitoes were dissected 13–15 days post-infective bloodmeal, and parasite detection was conducted through qPCR targeting the 18S ribosomal subunit in genomic DNA extracted from midguts and separately from head and/or thorax tissues for those mosquitoes that tested positive for midgut parasites.

Results from the fourth replicate indicated that although 36 (85%) of 42 MM-CP mosquitoes had detectable parasites in their midguts, none (0%) tested positive for parasites in their head and/or thorax, a proxy for salivary gland infection (Fig. 5d). By contrast, 82 (57%) of 143 wt control mosquitoes were midgut positive and 50 (35%) had head and/or thorax parasite presence, showing variable sporozoite loads. Similarly, in the fifth replicate, 56 (54%) of 104 MM-CP mosquitoes tested positive for midgut infection, but only 7 (7%) tested positive for salivary gland infection, all with very low infection levels (Fig. 5e). This contrasts with 104 wt mosquitoes of which 66 (63%) were midgut positive and 57 (55%) showed salivary gland infection.

These findings demonstrate that our earlier observations from a different *A. gambiae* MM-CP genetic background infected with the laboratory *P. falciparum* NF54 strain^38^, characterized by reduced and delayed sporozoite development, impaired oocyst maturation and limited salivary gland invasion, are recapitulated when MM-CP mosquitoes are challenged with genetically diverse *P. falciparum* isolates from malaria patients. This highlights the robustness of the MM-CP phenotype across parasite genotypes and reinforces its potential for impact under real-world transmission settings. Although we cannot fully exclude the possibility that low-level sporozoite development may eventually lead to transmission, the combination of delayed parasite maturation and reduced post-bloodmeal mosquito survival is projected to severely constrain the likelihood of onwards transmission under field conditions.

## Conclusion

Our study is breaking new ground towards the trialling and application of new genetic technologies to interrupt malaria transmission in Africa with the successful generation of genetically modified *A. gambiae* mosquitoes in Tanzania. By inhibiting *P. falciparum* oocyst growth, this engineered strain causes delayed sporozoite migration to the mosquito salivary glands, creating a barrier against malaria transmission. Its efficacy against field-derived parasites ensures that the findings are directly applicable to real-world transmission settings, providing a strong foundation for field testing. To ensure robustness across the continent, further validation is essential across a range of *A. gambiae* genetic backgrounds and environmental contexts. This must be accompanied by a comprehensive risk assessment framework that includes entomological and environmental evaluations to consider any potential or unintended affects. Monitoring for resistance in both mosquitoes and parasites is also key to ensure long-term effectiveness.

This research was executed within purpose-built, high-biosafety facilities in Tanzania, emphasizing the importance of African-led research infrastructure and expertise in advancing locally relevant innovation. Beyond enabling the present study, these facilities now serve as a scalable, sustainable platform for regional stakeholder engagement, training and future technology evaluation across the region. Our study illustrates the potential of gene drive technologies in malaria elimination and the critical need for continued investment in local capacity to ensure their safe, effective and equitable implementation.

## Methods

### Design and construction of the MPL/CL3 laboratory

Our design intent was to build a containerized insectary facility including a derogated BSL-3 laboratory with rearing areas for the culturing and manipulation of transgenic and infected mosquitoes completely within transportable ISO 668 series 1AAA (40 ft high cube) intermodal shipping containers, enabling cost-effective research in a disease endemic location. The project stages included the completion of the technical design, manufacture and/or construction, shipping to host site and finally the facilities in use. To enable reuse and modification for open malaria research the technical design plans are shared and accessible as part of this publication (Supplementary Note). Host facility provision of essential electrical and plumbing site service requirements and builders works to accept, connect to and operate the facility was incorporated into the design plan.

Characteristics of the MPL/CL3 facility include two self-contained modules comprising one laboratory module container and a second plant container (PCR) for the provision of all mechanical, electrical and general services. Notably all systems and equipment were shipped fully assembled and built in for delivery as a single package, to a concrete base prepared at the host site for connection to local services and infrastructure. Services connections between the laboratory container and plant container modules are specified in the technical plans (Supplementary Note).

The general layout of the laboratory module (drawing 2, layout) comprises fully sealed and insulated internal panels, partitions and doors to form four separate rooms making efficient use of space and functionality while ensuring biosafety and security. The laboratory module container layout incorporates (1) an entrance lobby; (2) a colony rearing room; (3) an infection, dissection and imaging room and (4) an infected mosquito room BSL-3 (drawing 10, sections laboratory module). All doors are self-closing and interlocked with access control on the main entry door. An emergency only exit panel is located to the rear. Laboratory benching, storage solutions and sinks are distributed within the four rooms. Supplied equipment include a glass washer, fridge freezer, autoclave and a Class 2 recirculating microbiological cabinet. Rooms (2) and (4) are equipped with self-contained incubators complete with lighting, temperature and humidity control. Room (3) is equipped with the Class 2 recirculating microbiological cabinet and an autoclave (drawing 3, equipment). The ventilation system serving the laboratory provides controlled airflow (drawing 12, HVAC (heating, ventilation and air conditioning) ducts) and a controlled air pressure regime (drawing 4, room pressures). Small power (drawing 20, power sockets), lighting (drawing 19, lighting) fire detection and alarm provision is incorporated throughout (drawing 8, fire protection). The PCR module consists of all essential mechanical, electrical and public health infrastructure to service the laboratory (drawing 11, sections PCR module). Ventilation systems include run and standby chillers, a dedicated air handling unit, general supply and extract air distribution including high efficiency particulate air-filtered extract (drawing 13, HVAC P&ID (piping and instrumentation diagram) schema; drawing 14, cooling and heating P&ID schema). Plumbing systems including hot and cold water services (drawing 15, cold and hot water P&ID schema) and purified water production and storage. A laboratory effluent treatment system comprising dosing tanks and pipework (drawing 16, waste effluent treatment P&ID). An electrical distribution cabinet serving the laboratory lighting, equipment and socket outlets; ventilation, plumbing and effluent systems and controls (drawing 24, modular laboratory general cabinet and HVAC). Uninterruptable power supply equipment provides back-up power to electrical systems including lighting, access control, fire alarm and controls (drawing 25, modular laboratory general cabinet uninterruptable power supply). The host facility assumed responsibility for the design and provision of essential site service requirements to support the containerized insectary facility including a 64-kW electricity supply (drawing 23, electrical installation general schema), plumbing comprising cold water and drainage services and a structural support or base. Host facility solutions for electricity included electrical supply cabling with connection to the national energy grid plus a back-up electrical generator and fuel storage. A purpose-built electrical installation building near to the plant container provides electrical supply cabling directly into the plant container. Piped cold water is provided into the plant container through a 7-metre elevated water tank with a soak away pit in place to receive piped treated wastewater from the plant container. In addition to the construction of a concrete base (drawing 29, MPL support; drawing 33, PCR module foundation), an insect moat, ramp, stairs and corrugated iron roof covering completed the site-specific building works.

### Mosquito husbandry

We used the *A. gambiae sensu stricto* Ifakara strain, derived from mosquitoes collected in Njage, Tanzania, in 1996 (ref. ^37^). Mosquitoes were maintained under optimized conditions: 27  ±  1 °C temperature, 70  ±  5% humidity and a 12 h/12 h dark/light cycle. Before floating, mosquito eggs were treated with a 1% bleach solution for 60 s. Larvae were fed with TetraMin fish flakes and reared at a density of 200 larvae per litre of deionized water from the L2 stage, ensuring a healthy mosquito population.

### Mosquito transgenesis

For the zpg-CC helper line, freshly laid embryos were microinjected with a mixture of donor plasmid pD-zpg-Cre-Cas9 (400 ng µl^−1^) and helper plasmid p165-KMO^44^ (200 ng µl^−1^). For the MM-CP^GFP^ line, embryos were injected with donor plasmid pD-Mag-Mel-CP (400 ng µl^−1^) and helper plasmid p155-vasa-Cas9 (200 ng µl^−1^). To establish a homozygous markerless MM-CP mosquito line, we developed a pupal case genotyping protocol. Freshly shed pupal cases were collected individually, and genomic DNA was extracted using 20 µl of dilution buffer from the Phire Tissue Direct PCR Master Mix kit (Thermo Scientific). Multiplex PCR was performed with primers: HA5′ CP (GGGTTAAGCTGGGCTCGTTG), Mag-R (AGTTCATGATCTCGCCCACG) and HA3′ CP (CTCCTTCGGATGCTCACTGG). The wt alleles yielded a 670-bp band, whereas MM-CP alleles produced a 357-bp band. Confirmed homozygotes were used to propagate the colony.

### Survival assays

For the zpg-CC *A. gambiae* line, triplicate groups of 15 female mosquitoes for each of the transgenic and wt lines were set up in standard insectary conditions. Cumulative mortality was recorded daily, and survival data were collected until all individuals had either died or were censored at the end of 25 days. Kaplan–Meier survival analysis was performed to generate survival curves, and differences between the transgenic and wt lines were evaluated using the log-rank test. For the MM-CP Ifakara line, 150 pupae from each of the MM-CP and wt controls were placed in separate BugDorm-4H3030 cages and allowed to emerge. Adults were maintained on a constant supply of 10% sugar solution. Mosquitoes were monitored daily, with dead individuals removed every 24 h, sexed and counted. Monitoring continued until all individuals in at least one cage had died. The assay was conducted in two biological replicates, each with a minimum of two technical replicates. Survival curves were compared between strains using the log-rank (Mantel–Cox) test in Prism v.10.

### Reproductive fitness assays

For the zpg-CC *A. gambiae* line, relative fecundity (number of eggs laid) and fertility (hatch rate) were assessed by placing single, mated and blood-fed female mosquitoes in individual cups 1 day after blood feeding. Each mosquito was allowed to lay eggs that were subsequently allowed to hatch. The total number of eggs and larvae were counted under a microscope to determine fecundity and fertility. For the MM-CP Ifakara line, 100 5-day-old females from each MM-CP and wt control lines were blood-fed through membrane feeders. Only fully engorged females were retained. Forty-eight hours post-bloodmeal, 25 females per strain were individually housed in oviposition cups lined with moist filter paper. After 72 h, eggs laid were counted. Cups were then topped with water to facilitate hatching. On day six, the number of larvae per cup was recorded. Data on the number of eggs per female and the hatch rate (larvae-to-egg ratio) were analysed using the Kolmogorov–Smirnov test in Prism v.10.

### Parasitological surveys

Participants were recruited from primary schools in Wami Mkoko and Miono villages during the school term, and children aged 6–14 were sampled in Kibindu village during the school closure period. Finger-prick blood samples were collected from children in good health who assented to the procedure and had written consent from a parent or guardian. A drop was used for a malaria RDT to identify infected participants, and another drop was used to prepare thick smears. Thick smears from participants who were RDT-positive were examined microscopically to detect and quantify gametocytes (per 500 white blood cells) by a certified microscopist using an OLYMPUS light microscope. Children with gametocyte density of ≥16 gametocytes per microlitre were candidates for blood drawing for DMFAs. All participants infected with malaria received treatment at the local clinic within 24 h, following the recommended protocol of oral Artemether-Lumefantrine.

### Informed consent

Parents of study participants or their legal guardians provided written informed consent before enrolment. The consent process was conducted in accordance with approved ethical guidelines and included a detailed explanation of the study aims, procedures (including blood sampling and mosquito feeding assays), potential risks (such as minor discomfort from blood draws) and expected benefits (including free malaria diagnosis and treatment for positive cases). Participants were informed of their right to withdraw at any time. Assent was also obtained from participating children, and a witness was present for consent procedures involving illiterate participants. The informed consent form was reviewed and approved by the IHI Institutional Review Board and the Tanzania National Institute for Medical Research.

### Phylogenetic profiling

Blood from malaria-infected patients was collected on QIAcard FTA Classic cards (Qiagen). *Plasmodium* genomic DNA was extracted from the dried blood spots using the DNeasy Blood & Tissue kit (Qiagen). Four genes were amplified from each sample using KAPA HiFi HotStart ReadyMix (Takara) as previously described^41^, and samples were library sequenced by use of Oxford Nanopore sequencing by Full Circle Laboratories (UK). Adaptor sequences were removed from raw read sequences with Cutadapt^45^ and mapped to the *P. falciparum* 3D7 genome (PlasmoDB v68) with BWA-MEM. Genetic variants were called and consensus sequences for each of the four genes assayed in each sample were generated with BCFtools^46^. Consensus sequences of each gene were concatenated for each sample, aligned against each other using the ‘msa’ package^47^ and were incorporated into a phylogenetic tree using the ‘ape’ package^48^ in RStudio (RStudio Team). Pairwise percentage identity values were calculated by aligning each concatenated sequence to the NF54 reference genome using pwalign in Bioconductor, applying the formula: 100 × (number of identical positions)/(aligned positions + internal gap positions). Sequence data have been deposited in the National Center for Biotechnology Information (NCBI) Sequence Read Archive (SRA) under BioProject accession number PRJNA1299763.

### Blood processing and mosquito DMFAs

Study participants with high gametocyte counts were invited, along with at least one parent or guardian, to the MPL/CL3 laboratory for blood donation, which was then used in mosquito DMFAs. Blood collected in lithium-heparin coated vacutainers were processed by centrifugation to separate cellular components from the serum that was then replaced with commercially available AB human serum at half the original volume. The prepared blood mixture was transferred to membrane feeders, and mosquitoes were allowed to feed on blood for 15 min. Cages were then moved to an incubator set at 27 °C and 75% relative humidity. After 48 h, mosquitoes were provided with a 10% sucrose solution changed daily, and dead, unfed females were removed.

### Mosquito dissection and oocyst detection

Parasite development, infection intensity and prevalence were analysed through systematic procedures across three independent infections. Midguts were dissected on day 9 post-blood feeding and stained with a 0.1% mercurochrome to facilitate oocyst identification, followed by microscopic examination. The diameter of oocysts was measured using ImageJ software. Dissections on day 9 assessed oocyst development, whereas dissections between days 13 and 15 evaluated sporozoite development in the salivary glands.

### qPCR for sporozoite detection

Genomic DNA was used in 20-μl qPCR with reverse transcription reactions with the Fast SYBR Green Master Mix kit (ThermoFisher) to quantify the *P. falciparum* 18S ribosomal RNA (rRNA) gene fragment, using primers and methods as described in ref. ^49^. Standard curves for both the Pf18S rRNA and the *A. gambiae* S7 reference gene were created through serial dilution of nucleic acid templates. Cycle threshold (*C*_t_) values were converted using these standard curves, and Pf18S rRNA *C*_t_ values were normalized to those of S7.

### Inclusion and ethics

This study was conducted with a commitment to inclusion, ethics and local engagement. Local researchers played a leading role throughout the process, including study design, implementation and data ownership, with roles and responsibilities agreed on in advance. The epidemiological and parasitological study was reviewed for posing no risks of stigmatization, discrimination or harm to participants. Written informed consent was obtained from parents or guardians, and oral assent was secured from children. No biological materials, cultural artefacts or traditional knowledge were transferred out of the country, unless specific transfer agreements were obtained. All research adhered to ethical guidelines and regulations, including the Declaration of Helsinki, ensuring that the rights of participants to withdrawal and privacy were protected throughout the study. Environmental and biorisk-related standards were carefully considered in constructing the infrastructure and carrying out the research. Approvals to carry out the research were obtained by IHI Institutional Review Board and the Tanzania Commission of Science and Technology. The study protocol was reviewed and approved by the IHI Institutional Biosafety Committee and the Tanzania National Institute for Medical Research.

### Reporting summary

Further information on research design is available in the Nature Portfolio Reporting Summary linked to this article.

## Online content

Any methods, additional references, Nature Portfolio reporting summaries, source data, extended data, supplementary information, acknowledgements, peer review information; details of author contributions and competing interests; and statements of data and code availability are available at 10.1038/s41586-025-09685-6.

## Supplementary information

Supplementary Information MPL/CL3 specifications and technical plans.

Reporting Summary

Supplementary Data Source Data Sheet 1. Raw data on fecundity and fertility of MM-CP and wt female mosquitoes. Individual-level data from two independent replicates assessing the reproductive output of MM-CP and wt females. Each row corresponds to a single female monitored for oviposition (fecundity) and larval hatching (fertility). For each sample, the total number of eggs laid and the number of larvae that successfully hatched were recorded. Zero values indicate females that did not lay eggs or whose eggs failed to hatch. This data sheet contains the raw data used in Fig. 3b,c. Source Data Sheet 2. Daily survival data of MM-CP and wt mosquitoes over 17 days. Raw data from two biological replicates (replicates 1 and 2), each containing three technical replicates per strain, to assess mosquito survival under insectary conditions. For each day, the number of dead female and male mosquitoes was recorded separately for each strain. Each entry indicates a single observed death event on the corresponding day and replicate. This data sheet contains the raw data used in Fig. 3d. Source Data Sheet 3. Prevalence of parasitaemia and gametocytaemia in children from three Tanzanian villages. Data from malaria surveys conducted in schoolchildren across Wami Mkoko, Miono Kikalo and Kibindu villages in coastal Tanzania. Surveys involved RDTs to identify malaria infections and thick blood smear microscopy to quantify parasitaemia and gametocytaemia. Data are disaggregated by village and survey round. This data sheet contains the raw data used in Fig. 4a. Source Data Sheet 4. Quantification and genotyping of oocysts in MM-CP and wt mosquitoes infected with patient-derived *P. falciparum* isolates. Detailed measurements of oocyst size and corresponding mosquito genotypes from infection experiments using field-derived *P. falciparum*. Midguts were dissected at day 9 post-infection, stained and imaged to quantify oocyst area, diameter and radius. Each midgut was individually linked to a mosquito carcass, from which selective genotyping was performed to determine the presence and zygosity of the MM-CP transgene. The experimental strain (condition), biological replicate, mosquito/gut ID, oocyst ID within a given gut, the area of the oocyst in micrometres square, the estimated radius and diameter of the oocyst in micrometres, and the gut genotype are identified. ND, not determined. This data sheet contains the raw data used in Fig. 5b,c. Source Data Sheet 5. Quantification of parasite dissemination to mosquito head and/or thorax tissues in MM-CP and wt mosquitoes. qPCR results for *P. falciparum* 18S rRNA gene quantification in head and/or thorax samples of MM-CP and wt mosquitoes at 13–15 days post-blood feeding during two independent transmission-blocking experiments (replicates 4 and 5). A value of 0.00 indicates no detectable parasite signal. Non-zero values reflect varying degrees of parasite load in the salivary glands. This data sheet contains the raw data used in Fig. 5d,e.

## Figures and Tables

**Extended Data Table 1:**
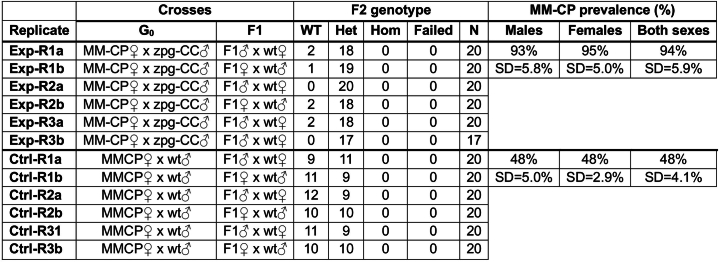
Inheritance rates of the MM-CP transgene in the presence or absence of Cas9

The table presents data from three biological replicates of inheritance assays to quantify the transmission rate of the MM-CP transgene under non-autonomous gene drive conditions. G_0_ MM-CP females were crossed to either zpg-CC males (expressing germline Cas9) or wt males. The resulting F_1_ progeny (zpg-CC;MM-CP or +;MM-CP) were then crossed to wt mosquitoes, and 20G_2_ progeny per replicate were genotyped to determine inheritance of the MM-CP transgene. Data are presented separately for male and female G_1_ crosses. Note that in replicate Exp-R3b, only 17 mosquitoes could be genotyped.

## Data Availability

The datasets generated and analysed during this study are available in the paper, Extended Data Fig. 1, Extended Data Table 1 and the Supplementary Information. Raw sequencing data are available under BioProject accession PRJNA1299763 (NCBI SRA).
