# Supplementary material for: Gene-drive-capable mosquitoes suppress patient-derived malaria in Tanzania
Source: Nature. 2025 Dec 10;649(8096):442–8. doi: 10.1038/s41586-025-09685-6 (PMC12779567; doi:10.1038/s41586-025-09685-6)
Supplement: Supplementary file 1 — MPL/CL3 specifications and technical plans. [file 41586_2025_9685_MOESM1_ESM.pdf]

nature portfolio

https://doi.org/10.1038/s41586-025-09685-6

Supplementary information

Gene-drive-capable mosquitoes suppress patient-derived malaria in Tanzania

In the format provided by the authors and unedited

transmissionzero

IMPERIAL

Project: **MODULAR PORTABLE LABORATORY - IMPERIAL COLLEGE**

AS BUILT

Nº	DOCUMENT	DRAWING CODE
1	OVERVIEW	N/A
2	LAYOUT	15549-PB-LY-00
3	EQUIPMENT	15549-PB-LY-01
4	ROOM PRESSURES	15549-PB-LY-02
5	BIOSAFETY LEVELS	15549-PB-LY-03
6	PEOPLE, MATERIALS AND WASTE FLOW	15549-PB-LY-04
7	MAIN EQUIPMENT LOCATION	15549-PB-LY-05
8	FIRE PROTECTION	15549-PB-LY-06
9	CEILING ELEMENTS ARRANGEMENT	15549-PB-LY-07
10	SECTIONS LAB MODULE	15549-PB-LY-08
11	SECTIONS PCR MODULE	15549-PB-LY-09
12	HVAC DUCTS	15549-PB-CL-00
13	HVAC P&ID SCHEMA	15549-CJ-EP-00
14	COOLING-HEATING P&ID SCHEMA	15549-CJ-EP-01
15	COLD AND HOT WATER P&ID SCHEMA	15549-CJ-EP-02
16	WASTE EFFLUENT TREATMENT P&ID	15549-CJ-EP-03
17	GENERAL SERVICES	15549-PB-TB-00
18	PIPES DISTRIBUTION AND DRAINAGE	15549-PB-TB-01
19	LIGHTING	15549-PB-EC-00
20	POWER SOCKETS	15549-PB-EC-01
21	ELECTRICAL CHANNELS	15549-PB-EC-02
22	POWER INCOME CONNECTION BOX	15549-PB-EC-03
23	ELECTRICAL INSTALLATION GENERAL SCHEMA	15549-CJ-EE-00
24	MODULAR LABORATORY GENERAL CABINET (& HVAC)	15549-CJ-EE-01
25	MODULAR LABORATORY GENERAL CABINET (U.P.S.)	15549-CJ-EE-02
26	MODULAR LABORATORY LIGHTING, EQUIPMENT AND SOCKETS CABINET	15549-CJ-EE-03
27	LIGHTNING PROTECTION DETAILS	15549-CJ-EE-04
28	MAGNEHELIC PLATE	15549-DE-00
29	MPL SUPPORT	15549-DE-01
30	PRESSURE PLATES	15549-DE-02
31	RECEPTION TANK & PUMP	15549-DE-03
32	CONNECTION HOLES & REGISTRY CASES LOCATION	15549-DE-04
33	PRC MODULE FOUNDATION	15549-DE-05
34	PCR MODULE DOORS DETAIL	15549-DE-06
35	EMERGENCY DOOR CLOSURE DETAIL	15549-DE-07
36	GRID DETAIL	15549-DE-08
37	EMERGENCY DOOR SIZE	15549-DE-09
38	LABORATORY CONTAINER. HOLES	15549-DE-10
39	SECONDARY INSTALATIONS CONTAINER. HOLES	15549-DE-11

02-Layout

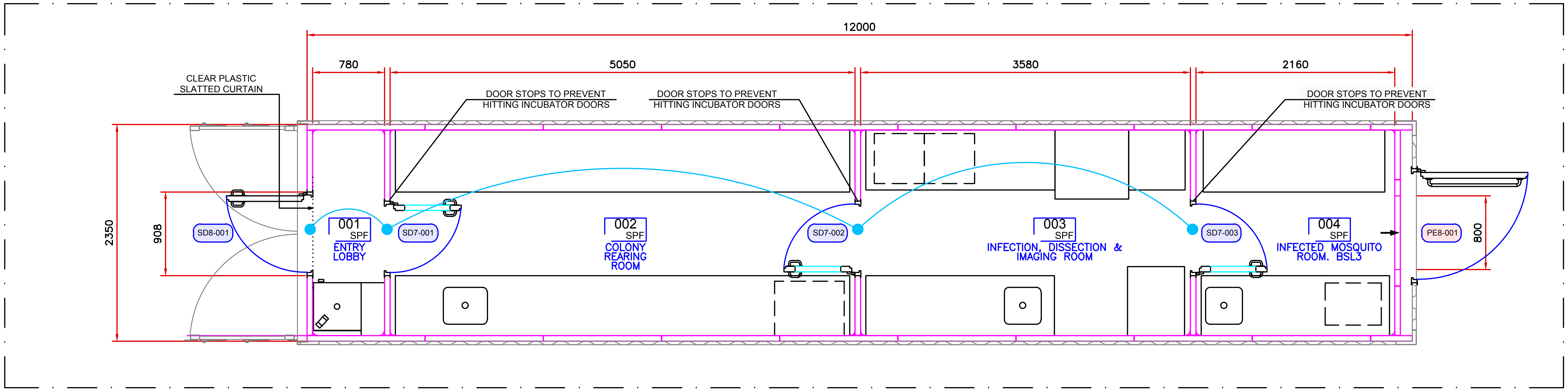

Lay-out
scale 1/50

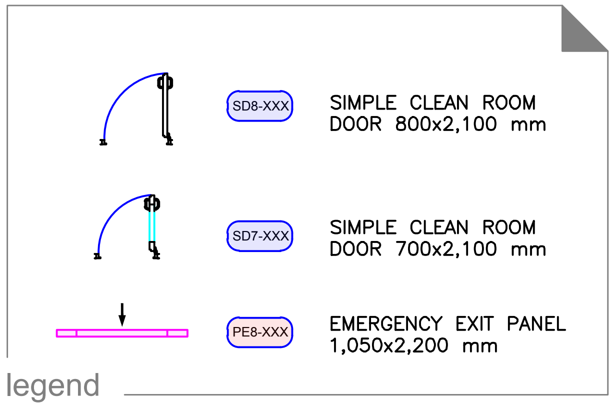

ROOM DESCRIPTION

Nº	NAME	AREA (m²)	CLAS. (ISO)	HEIGHT (m)
001	ENTRY LOBBY	1,74	SPF	2.30
002	COLONY REARING ROOM	11,26	SPF	2.30
003	INFECTION, DISSECTION & IMAGING ROOM	7,98	SPF	2.30
004	INFECTED MOSQUITO ROOM	4,82	SPF	2.30
	TOTAL	25.80		

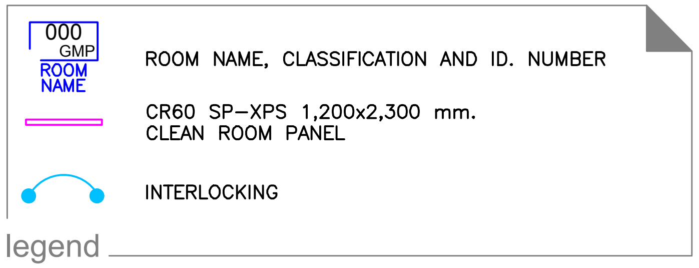

drawing scale 1:50

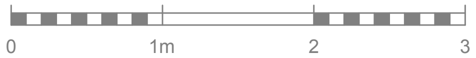

A3
420x297

transmission zero

IMPERIAL

project	title		scale / drawing
Modular Portable Laboratory	TECHNICAL DESIGN PROJECT LAYOUT	2	scale 1/50; drawing 15549-PB-LY-00

03-Equipment

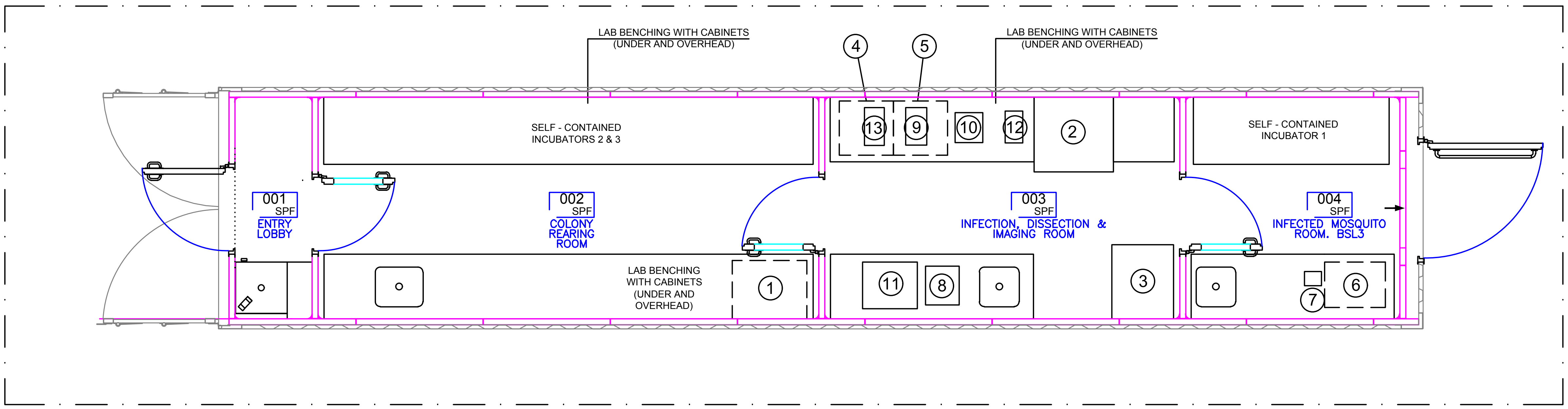

Lay-out
scale 1/50

DESCRIPTION OF EQUIPMENT

Nº	NAME	WEIGHT (kg)	DIMENSIONS (mm)	HEIGHT (mm)	POWER (kW)
1	DISHWASHER MIELE PG 8504 [AD]	74	600x600	835	9.20 kW
2	CLASS II MICROBIOLOGICAL SAFETY CABINET GUARDIAN MSC800	135	800x750	1321	0.15 kW
3	TOUCHCLAVE TCR/40/H1-SPECIAL AUTOCLAVE	120	580x720	710	2.30 kW
4	FRIDGE LIEBHERR TP 1410	35.7	554x623	850	0.19 kW
5	FREEZER LIEBHERR GP 1376	39.3	553x624	851	0.30 kW
6	BENCHTOP GLOVE BOX ELECTRO-TECH 5503 SERIES	31	610x457	381	
7	CIRCULATING BATHS GRANT TC120	2.3	172x141	333	1.40 kW
8	FLUORESCENT STEREO MICROSCOPE LEICA BIOSCIENCE, M205 FA		340x390	547	
9	STANDARD LIGHT MICROSCOPE OLYMPUS CX43	7.3	211x376	393	
10	DISSECTION/ STEREO MICROSCOPE LEICA BIOSYSTEMS M80		278x301	395	
11	T CONTROLLED CENTRIFUGE: EPPENDORF CENTRIFUGE 5804	55	470x550	340	0.90 kW
12	LABORATORY LIQUID SUCTION SYSTEM INTEGRA VACUSAFE	3.4	180x320	530	
13	HEATING BLOCK GRANT QBD4		380x200	100	0.60 kW

legend

000 GMP ROOM NAME — ROOM NAME, CLASSIFICATION AND ID. NUMBER
CR60 SP–XPS 1,200x2,300 mm. CLEAN ROOM PANEL
CLEAN ROOM WINDOW

transmissionzero

IMPERIAL

drawing scale 1:50
0 1m 2 3

A3 420x297

project: Modular Portable Laboratory

TECHNICAL DESIGN PROJECT

title: EQUIPMENT

3

scale 1/50

drawing 15549-PB-LY-01

04-Room Pressures

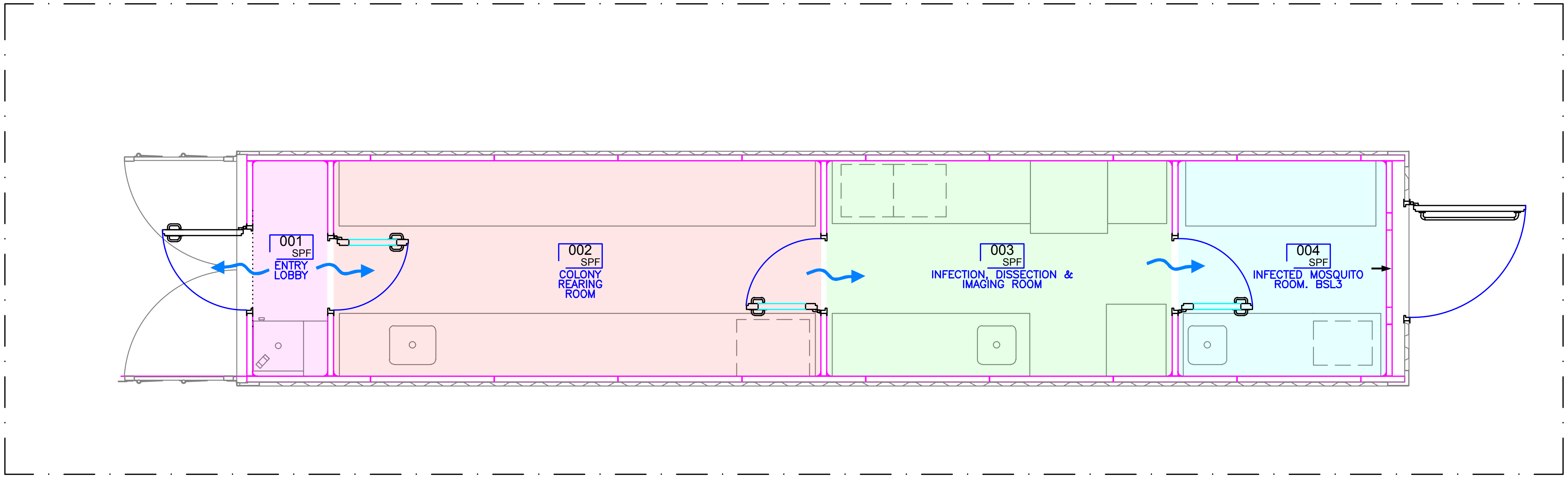

Lay-out

scale 1/50

000 GMP ROOM NAME	ROOM NAME, CLASSIFICATION AND ID. NUMBER
(blue wavy arrow)	AIR FLOW
(pink)	PRESSURE +15 (Pa)
(light red)	PRESSURE 0 (Pa)
(green)	PRESSURE −15 (Pa)
(cyan)	PRESSURE −30 (Pa)

legend

drawing scale 1:50

0 1m 2 3

A3
420x297

transmissionzero

IMPERIAL

project Modular Portable Laboratory

TECHNICAL DESIGN PROJECT

title ROOM PRESSURES

4

scale 1/50

drawing 15549-PB-LY-02

05-Biosafety Levels

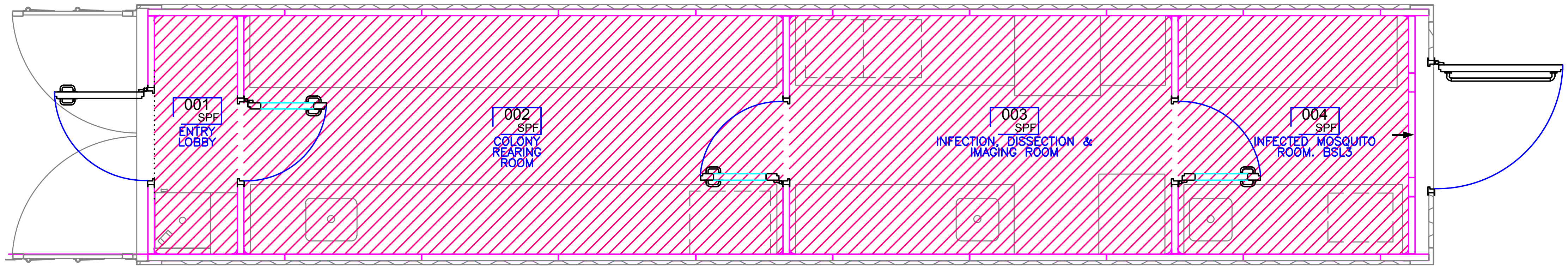

Lay-out
scale 1/50

000
GMP
ROOM
NAME
ROOM NAME, CLASSIFICATION AND ID. NUMBER

NC – NOT CLASSIFIED

BSL3 – BIOSAFETY LEVEL 3

legend

transmission zero

IMPERIAL

drawing scale 1:50
0 1m 2 3

A3
420x297

project	title		
Modular Portable Laboratory	TECHNICAL DESIGN PROJECT<br>BIOSAFETY LEVELS	5	scale 1/50<br>drawing 15549-PB-LY-03

06-People, Materials and Waste Flow

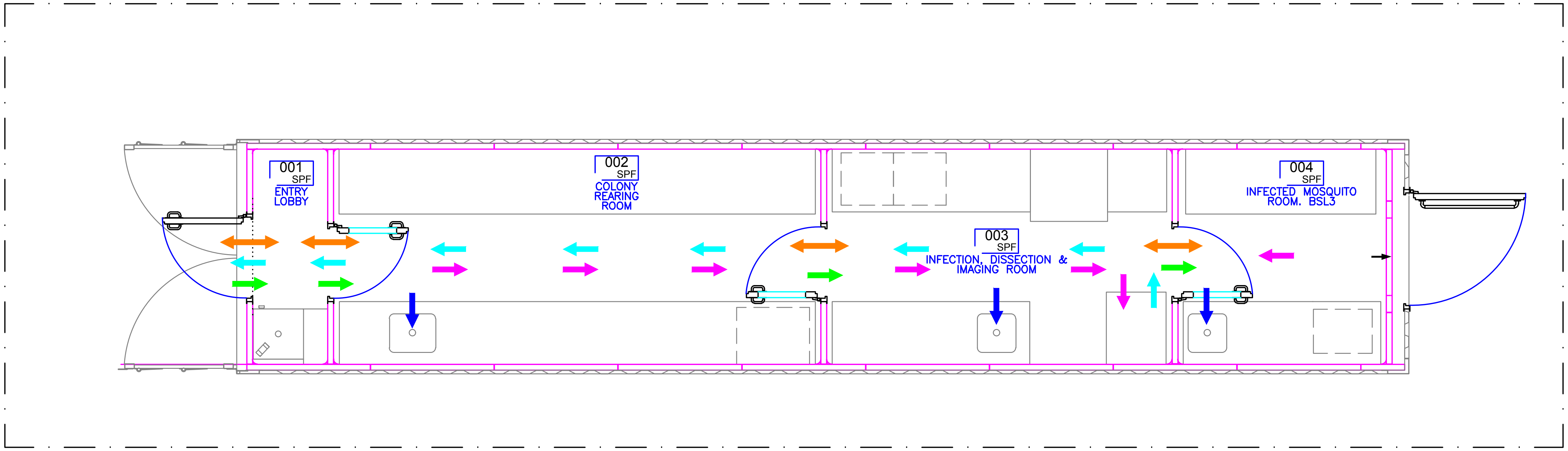

Lay-out
scale 1/50

legend

- 000 GMP ROOM NAME — ROOM NAME, CLASSIFICATION AND ID. NUMBER
- LIQUID WASTE FLOW
- SOLID WASTE FLOW
- STERILISED SOLID WASTE FLOW
- PEOPLE FLOW
- MATERIALS FLOW

drawing scale 1:50
0 1m 2 3

transmission zero
IMPERIAL

project	title		scale / drawing
Modular Portable Laboratory	TECHNICAL DESIGN PROJECT PEOPLE, MATERIALS AND WASTE FLOW	6	scale 1/50; drawing 15549-PB-LY-04

A3 420x297

07-Main Equipment Location

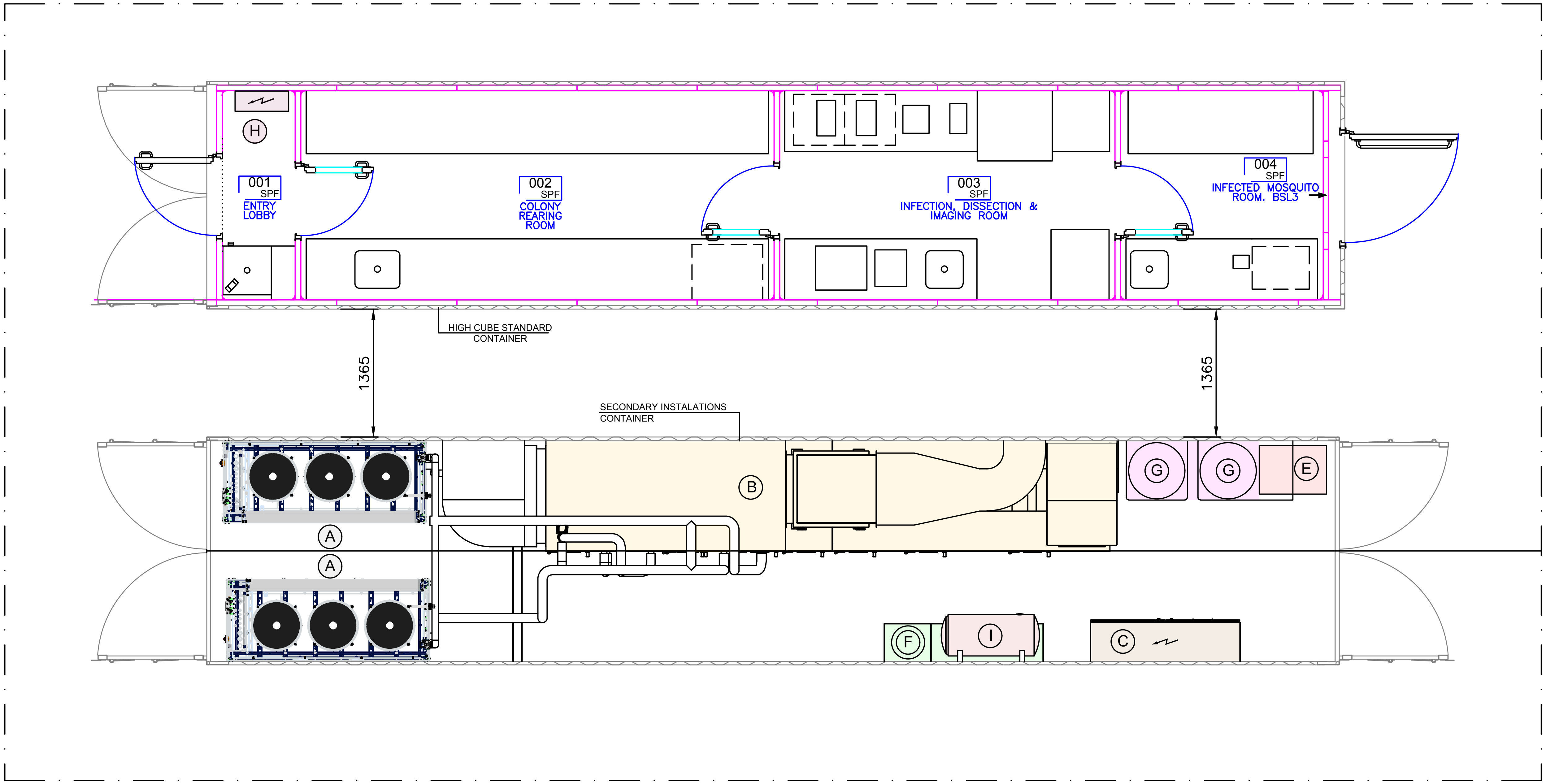

Lay-out
scale 1/50

A	RUN & STANDBY CHILLERS	F	CWS TANK AND BOOSTER SET
B	AIR HANDLING UNIT	G	EFFLUENT TREATMENT PLANT
C	ELECTRICAL GENERAL & HVAC CABINET	H	LIGHTING, EQUIPMENT AND SOCKETS CABINET
E	RO WATER PRODUCTION & STORAGE SYSTEM	I	HOT WATER TANK

legend

drawing scale 1:50
0 1m 2 3

A3
420x297

transmission zero
IMPERIAL

project: Modular Portable Laboratory
title: TECHNICAL DESIGN PROJECT
MAIN EQUIPMENT LOCATION
7
scale 1/50
drawing 15549-PB-LY-05

08-Fire Protection

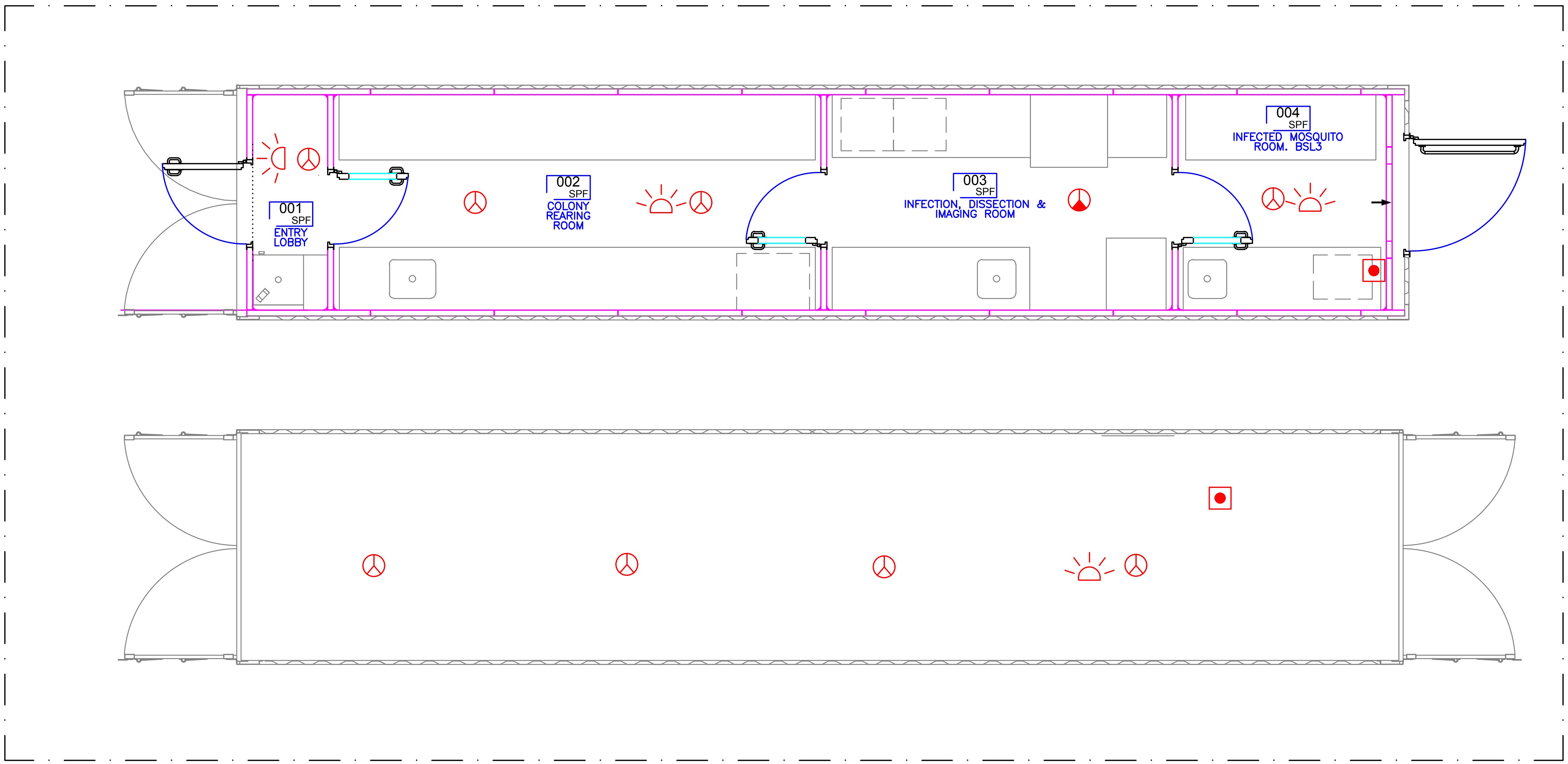

Lay-out
scale 1/50

legend

- 000 GMP ROOM NAME — ROOM NAME, CLASSIFICATION AND ID. NUMBER
- CALL POINT
- OPTICAL DETECTOR
- HEAT DETECTOR
- SOUNDER BEACON

drawing scale 1:50

0 1m 2 3

transmissionzero

IMPERIAL

A3
420x297

project	title		scale / drawing
Modular Portable Laboratory	TECHNICAL DESIGN PROJECT<br>FIRE PROTECTION	8	scale 1/50<br>drawing 15549-PB-LY-06

09-Ceiling Elements Arrangement

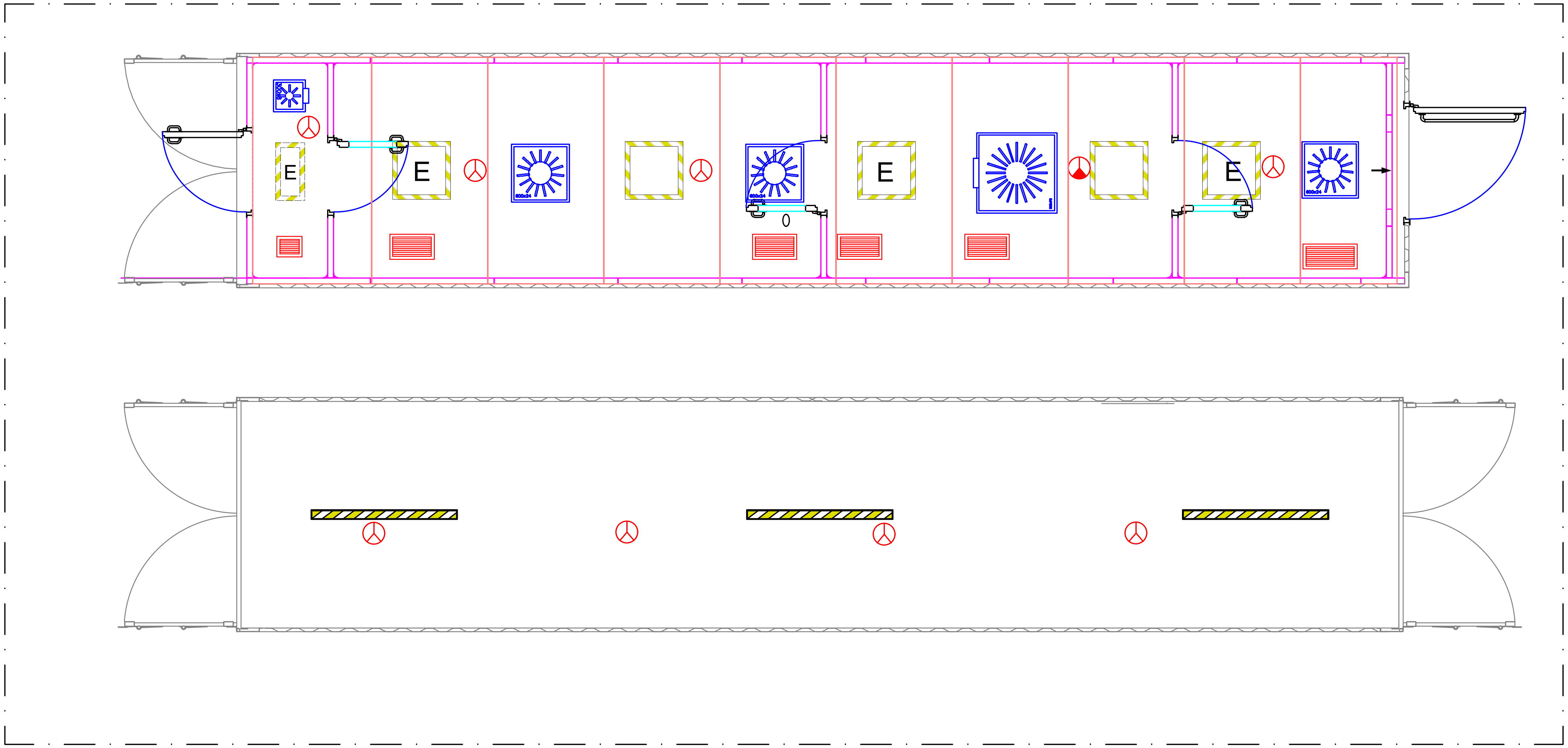

Lay-out

scale 1/50

	LED AIRTIGHT LAMP IP65<br>46x83x1,500mm, 60W, 5,400 Lm.
	LED LAMP 600x600 mm ACCESSIBLE FROM BELOW
E	LED LAMP 600x600 mm ACCESSIBLE FROM BELOW<br>WITH EMERGENCY KIT (3 HOURS)
E	LED LAMP 300x600 mm ACCESSIBLE FROM BELOW<br>WITH EMERGENCY KIT (3 HOURS)
	CR30 SP–XPS 1,200x2,350 mm.<br>CEILING PANEL
	CEILING GRILL
	SWIRL DIFFUSER
	OPTICAL DETECTOR
	HEAT DETECTOR

legend

drawing scale 1:50

0 1m 2 3

A3
420x297

transmissionzero

IMPERIAL

project: Modular Portable Laboratory	title: TECHNICAL DESIGN PROJECT<br>CEILING ELEMENTS ARRANGEMENT	9	scale 1/50<br>drawing 15549-PB-LY-07

SECTION A-A

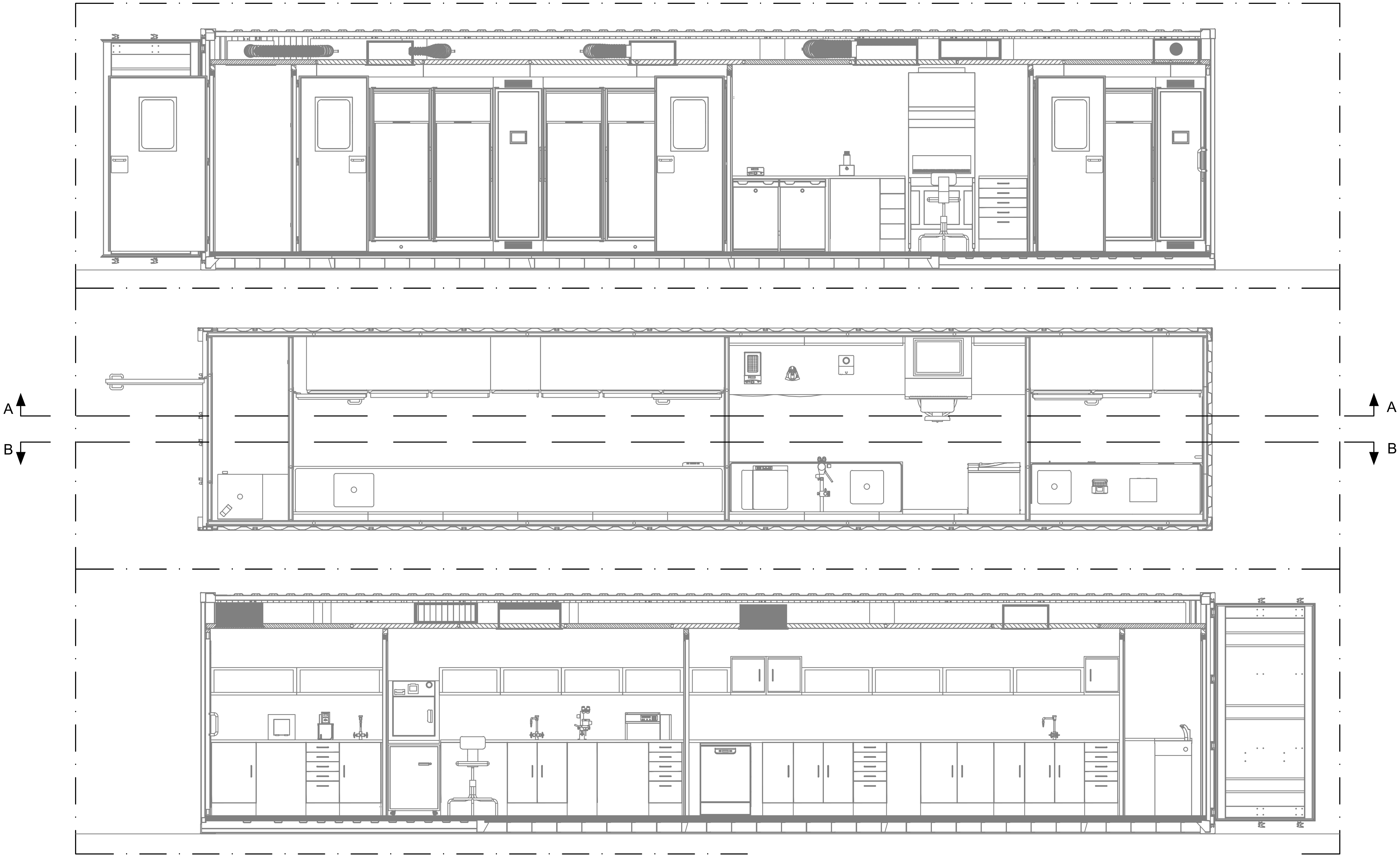

SECTION B-B

10-Sections Lab Module

drawing scale 1:50

0 1m 2 3

transmissionzero

IMPERIAL

A3
420x297

project: Modular Portable Laboratory	title: TECHNICAL DESIGN PROJECT — SECTIONS LAB MODULE	10	scale: 1/50
			drawing: 15549-PB-LY-08

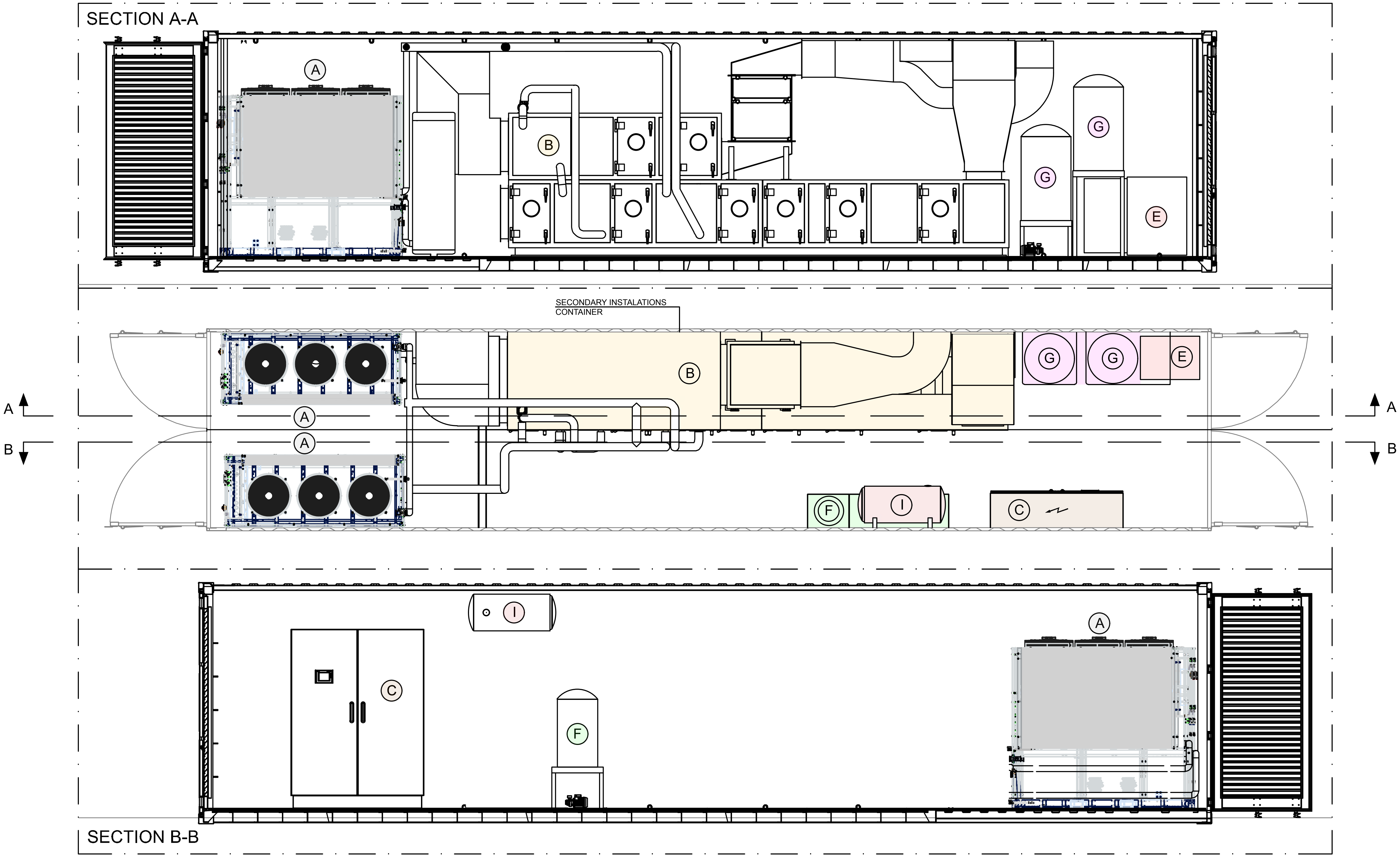

11-Sections PCR Module

legend

A	RUN & STANDBY CHILLERS	F	CWS TANK AND BOOSTER SET
B	AIR HANDLING UNIT	G	EFFLUENT TREATMENT PLANT
C	ELECTRICAL GENERAL & HVAC CABINET	I	HOT WATER TANK
E	RO WATER PRODUCTION & STORAGE SYSTEM		

drawing scale 1:50

0 1m 2 3

transmissionzero

IMPERIAL

project: Modular Portable Laboratory	title: TECHNICAL DESIGN PROJECT<br>SECTIONS PRC MODULE	11	scale: 1/50<br>drawing: 15549-PB-LY-09

A3
420x297

12-HVAC Ducts

EXHAUST AIR GRILLES

MODEL	SIZE (mm)	CODE
TROX AR-AG	225X125	EAG-01
TROX AR-AG	425x225	EAG-02
TROX AR-AG	425x225	EAG-03
TROX AR-AG	425x225	EAG-04
TROX AR-AG	425x225	EAG-05
TROX AR-AG	525x225	EAG-06

DIFFUSERS

MODEL	SIZE (mm)	CODE
TROX VDW-Q-Z-H-M	300x8	DF-01
TROX VDW-Q-Z-H-M	600x24	DF-02
TROX VDW-Q-Z-H-M	600x24	DF-03
TROX VDW-Q-Z-H-M	825x72	DF-04
TROX VDW-Q-Z-H-M	600x24	DF-05

AIR DAMPERS

OPERATION	SIZE (mm)	CODE
MOTORIZED	200x210	MAD-01
MOTORIZED	300x210	MAD-02
MOTORIZED	300x210	MAD-03
MOTORIZED	200x210	MAD-04
MANUAL	200x210	AD-01
MANUAL	400x210	AD-02
MANUAL	400x210	AD-03

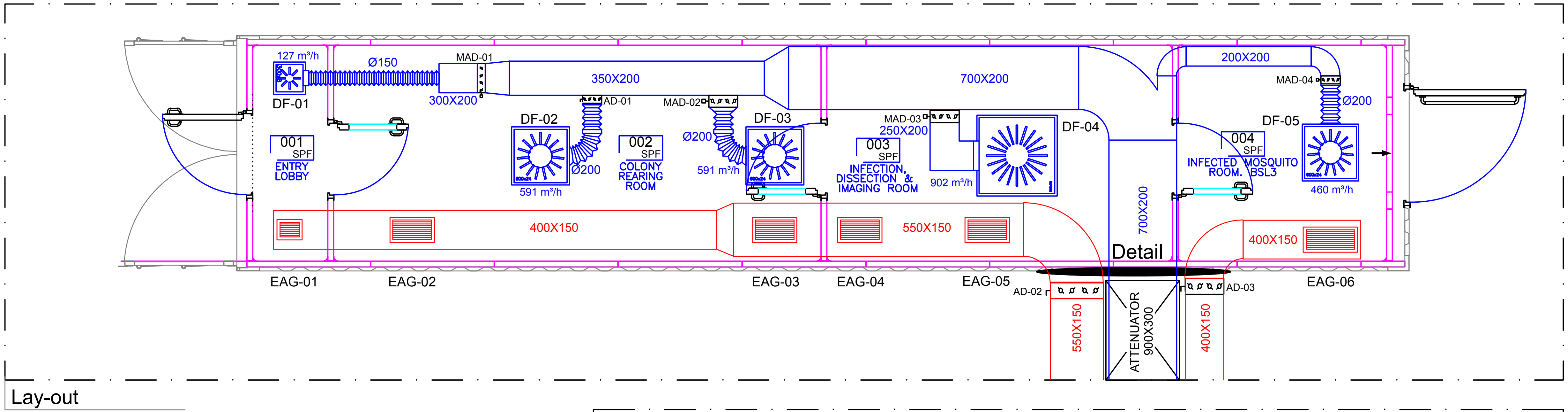

Lay-out
scale 1/50

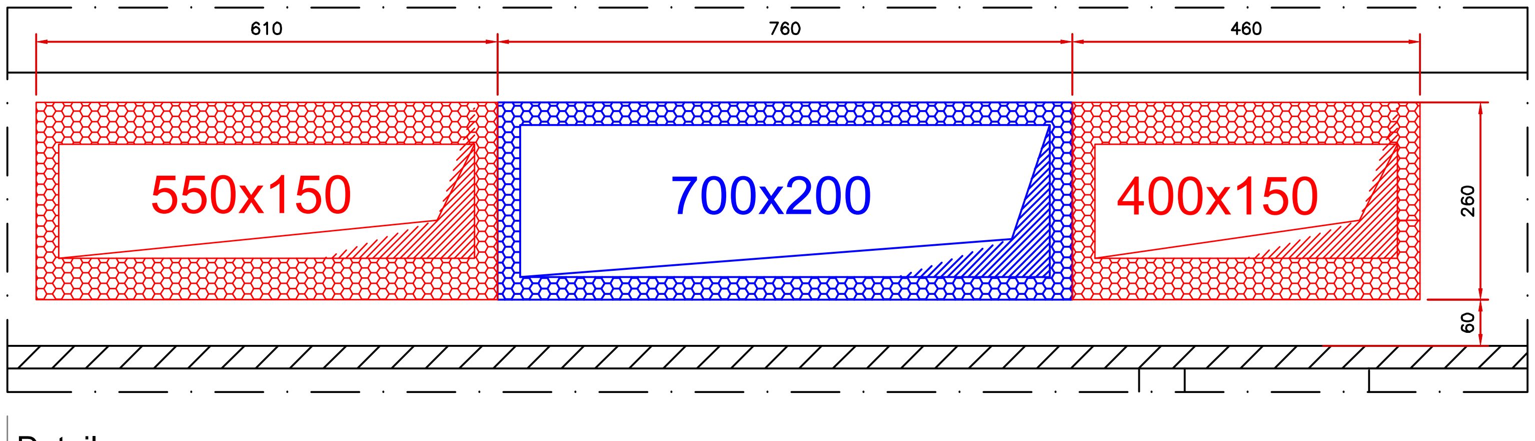

Detail
scale 1/10

000 ISO 8 ROOM NAME	ROOM NAME, CLASSIFICATION & ID. NUMBER
	SUPPLY AIR DUCT (GALVANISED STEEL)
	EXHAUST AIR DUCT (GALVANISED STEEL)
	FRESH AIR DUCT
	PVC FLEXIBLE CONNECTION
	SWIRL DIFFUSER
	MOTORIZED AIR DAMPER
	EXHAUST AIR GRILLE (CEILING)

legend

drawing scale 1:50
0 1m 2 3

transmission zero

IMPERIAL

A3
420x297

project: Modular Portable Laboratory

title: TECHNICAL DESIGN PROJECT
HVAC AIR DUCTS

12

scale 1/50

drawing 15549-PB-CL-00

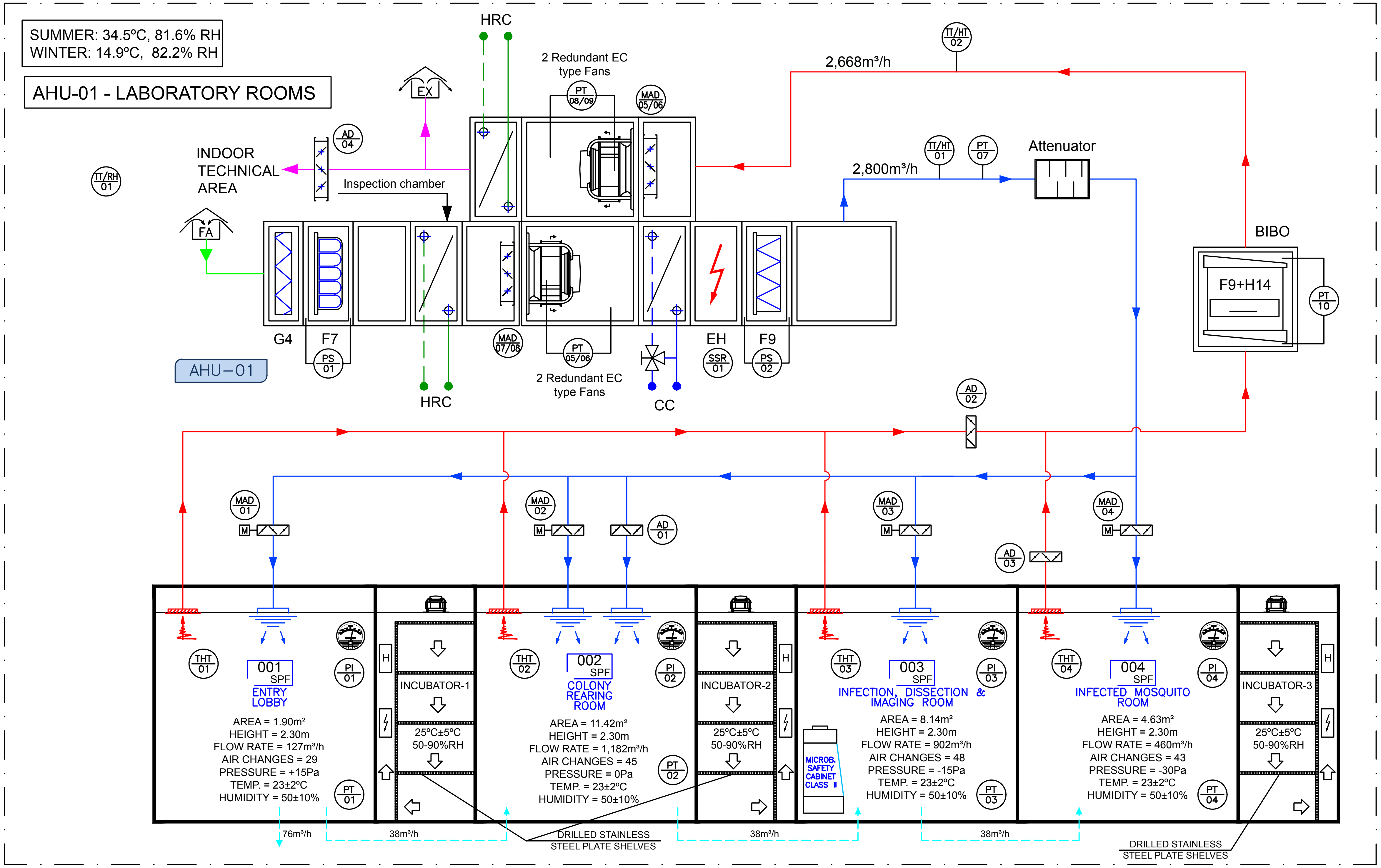

LEGEND

G4	MAT FILTER (PRE FILTER)
F7	HIGH EFFICIENCY FILTER
F9	HIGH EFFICIENCY FILTER
H14	HEPA FILTER
BIBO	BAG IN BAG OUT
CC	COOLING COIL (WATER)
HRC	EXHAUST AIR HEAT RECOVERY COIL
EH	ELECTRIC HEATER

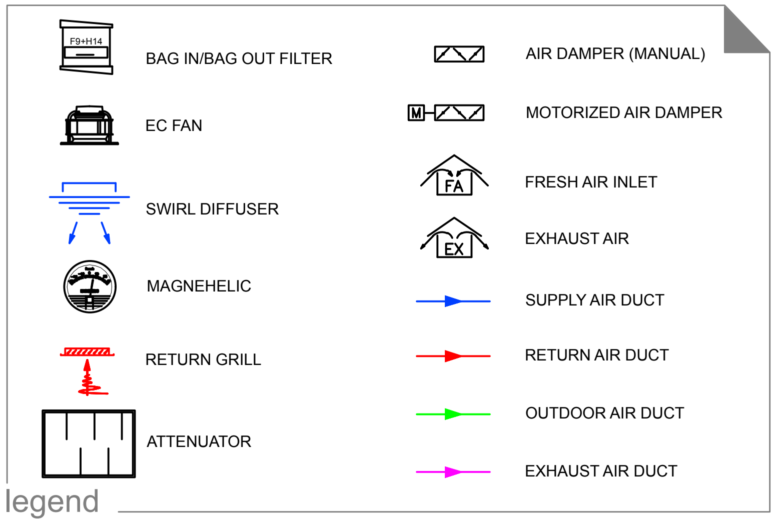

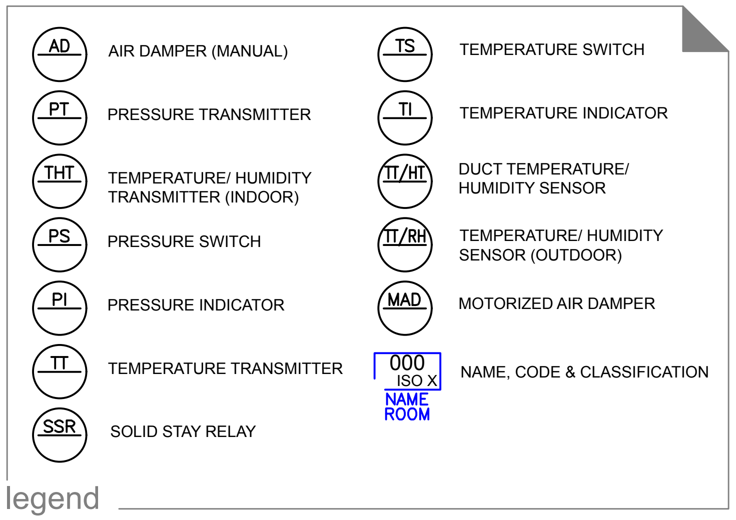

13-HVAC P&ID Schema

transmissionzero

IMPERIAL

NOTES:
1. THE AIR HANDLING UNIT HAS INTERNAL LIGHTS IN ALL INSPECTION CHAMBERS

A3 420x297

project: Modular Portable Laboratory	TECHNICAL DESIGN PROJECT — title: HVAC P&ID SCHEMA	13	scale: N/S	drawing: 15549-CJ-EP-00

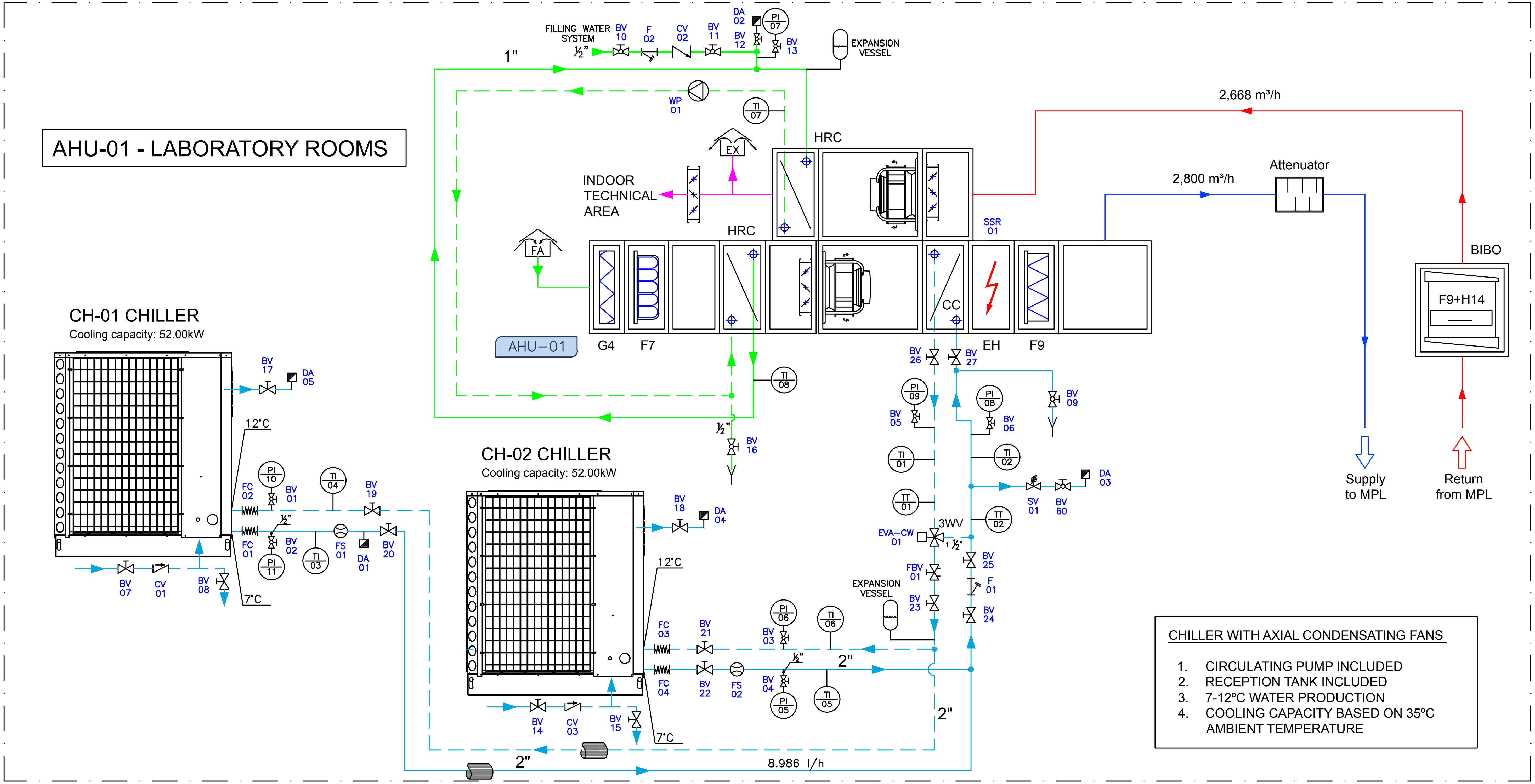

CHILLER WITH AXIAL CONDENSATING FANS

1. CIRCULATING PUMP INCLUDED
2. RECEPTION TANK INCLUDED
3. 7-12ºC WATER PRODUCTION
4. COOLING CAPACITY BASED ON 35ºC AMBIENT TEMPERATURE

LEGEND	
3WV	3 WAY VALVE (MOTORIZED)
EVA-CW	3 WAY VALVE ACTUATOR
BV	SHUT OFF VALVE (MANUAL)
FBV	FLOW BALANCING VALVE
F	FILTER
FC	FLEXIBLE CONNECTOR
TT	TEMPERATURE TRANSMITTER
TI	TEMPERATURE INDIC. (TEMP. GAUGE)
PI	PRESSURE INDIC. (PRESSURE GAUGE)
FS	FLOW SWITCH
DA	DEAERATOR
FC	FLEXIBLE CONNECTOR

SHUT-OFF VALVE	TEMPERATURE INDICATOR (TEMP. GAUGE)
FLOW BALANCING VALVE	PRESSURE INDICATOR (PRESSURE GAUGE)
3 WAY CONTROL VALVE	TEMPERATURE TRANSMITTER
CHECK VALVE	PIPE ISOLATION
SAFETY VALVE	WATER PUMP (hydraulic module included)
FILTER	FLOW SWITCH
FLEXIBLE CONNECTOR	
DEAERATOR	

symbols

NOTES:

1. FLEXIBLE CONNECTORS TO PREVENT THE VIBRATION OF THE PUMPS AND CHILLERS FROM RUNNING ALONG THE PIPES (THEY ALSO ALLOW THE PIPES TO EXPAND AND CONTRACT SLIGHTY WICH TAKES THE PRESSURE OFF THE CONNECTION)

14-Cooling-Heating P&ID Schema

transmission zero

IMPERIAL

project	title		scale / drawing
Modular Portable Laboratory	TECHNICAL DESIGN PROJECT<br>COOLING/HEATING P&ID SCHEMA	14	scale N/S<br>drawing 15549-CJ-EP-01

A3 420x297

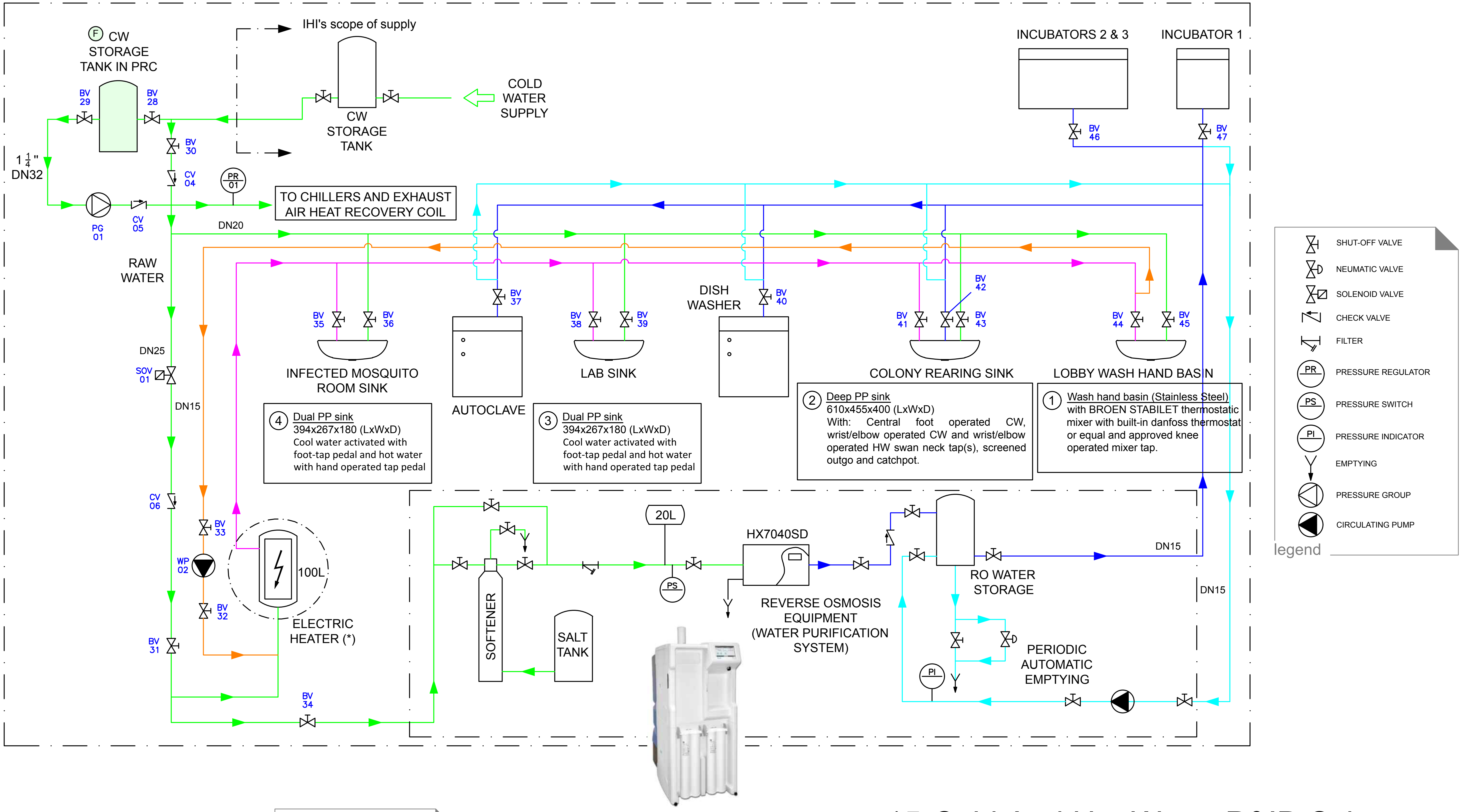

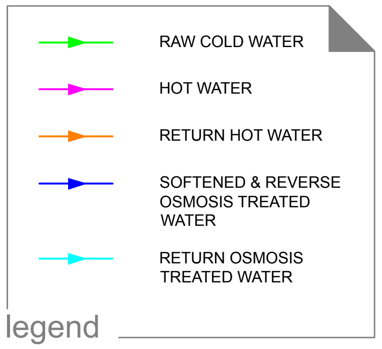

15-Cold And Hot Water P&ID Schema

transmission zero

IMPERIAL

NOTES:

1. THE MILIPORE PW PRODUCTION PLANT INCLUDES PRE–TREATMENT, PW STORAGE AND CIRCULATING PUMP
2. (*) CHECK VALVE INCLUDED IN THE ELECTRIC HEATER

A3 420x297

project: Modular Portable Laboratory	title: TECHNICAL DESIGN PROJECT COLD AND HOT WATER P&ID SCHEMA	15	scale: N/S; drawing: 15549-CJ-EP-02

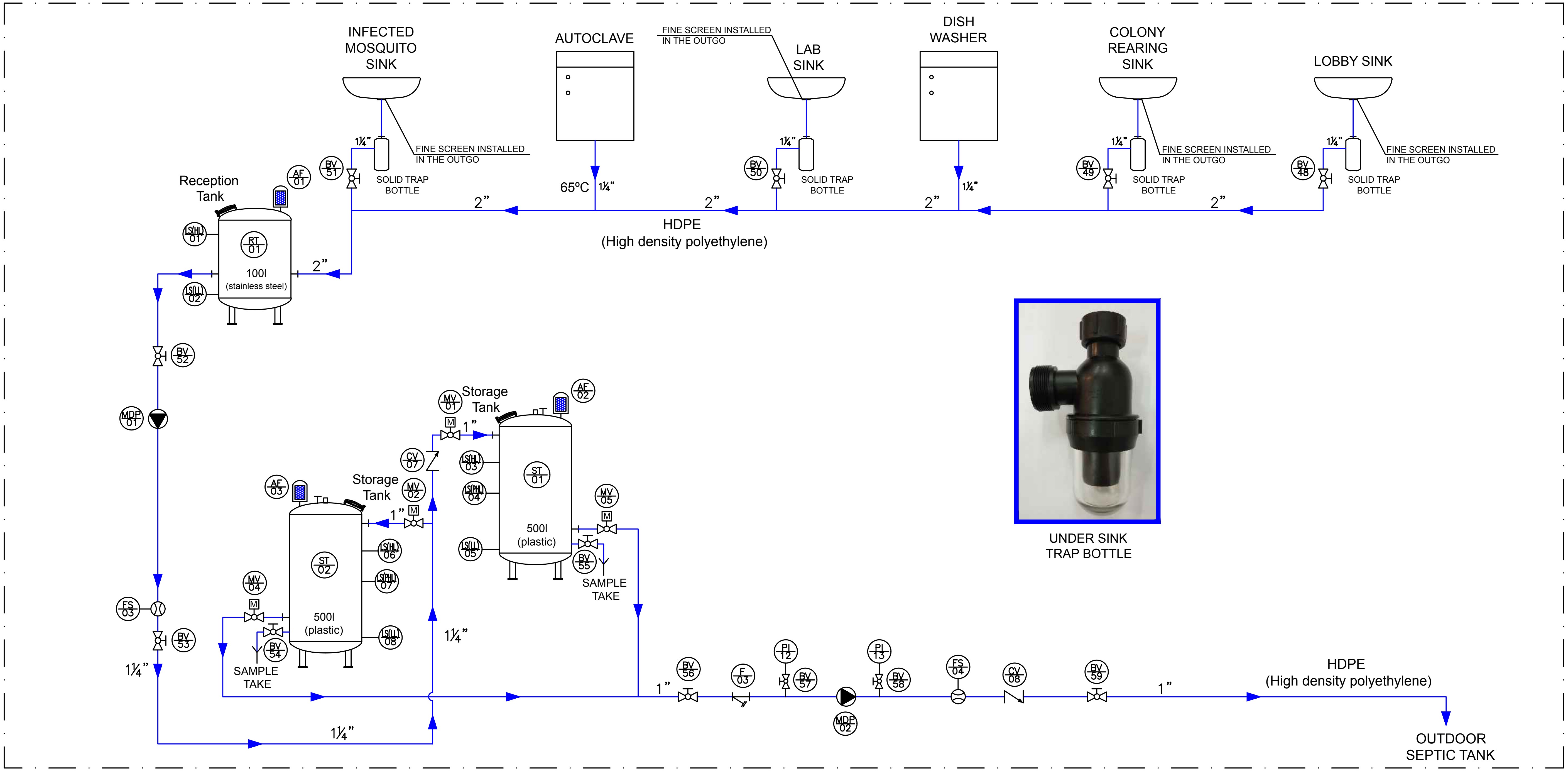

LEGEND	
MDP	WATER MAGNETIC DRIVE PUMP
AF / F	AIR FILTER / WATER FILTER
FS	FLOW SWITCH
CV	CHECK VALVE
BV	BALL VALVE
MV	MOTORIZED SHUT-OFF VALVE
ST	STORAGE TANK
LS	LEVEL SWITCH
PS	PRESSURE SWITCH
PI	PRESSURE INDIC. (PRESSURE GAUGE)

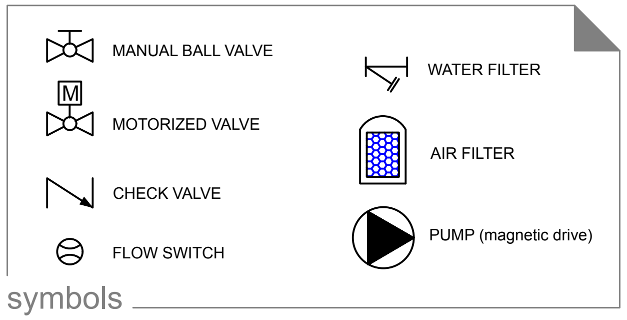

16-Waste Effluent Treatment P&ID

transmission zero

IMPERIAL

NOTES:

1. EFFLUENT TREATMENT SYSTEM CONTROLLED BY A PLC

A3
420x297

project	title		scale / drawing
Modular Portable Laboratory	TECHNICAL DESIGN PROJECT<br>WASTE EFFLUENT TREATMENT P&ID	16	scale N/S<br>drawing 15549-CJ-EP-03

17-General Services

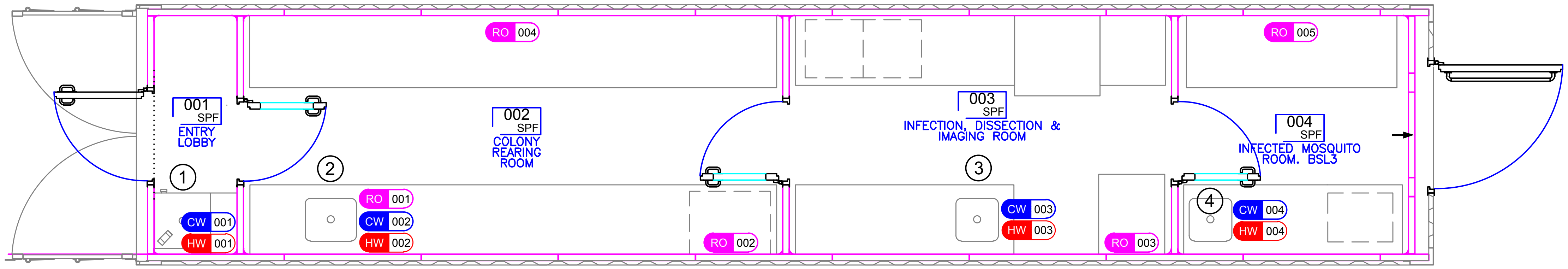

1. Wash hand basin (Stainless Steel) with BROEN STABILET thermostatic mixer with built-in danfoss thermostat or equal and approved knee operated mixer tap.

2. Deep PP sink 610x455x400 (LxWxD) With: Central foot operated CW, wrist/elbow operated CW and wrist/elbow operated HW swan neck tap(s), screened outgo and catchpot.

3. Dual PP sink 394x267x180 (LxWxD) Cool water activated with foot-tap pedal and hot water with hand operated tap pedal

4. Dual PP sink 394x267x180 (LxWxD) Cool water activated with foot-tap pedal and hot water with hand operated tap pedal

Lay-out
scale 1/50

legend

000 GMP ROOM NAME	ROOM NAME, CLASSIFICATION AND ID. NUMBER
CW 000	COLD WATER
HW 000	HOT WATER
RO 000	REVERSE OSMOSIS WATER (TYPE II)

drawing scale 1:50
0 1m 2 3

A3
420x297

transmission zero

IMPERIAL

project: Modular Portable Laboratory	TECHNICAL DESIGN PROJECT<br>title: GENERAL SERVICES	17	scale 1/50<br>drawing 15549-PB-TB-00

18-Pipes Distribution and Drainage

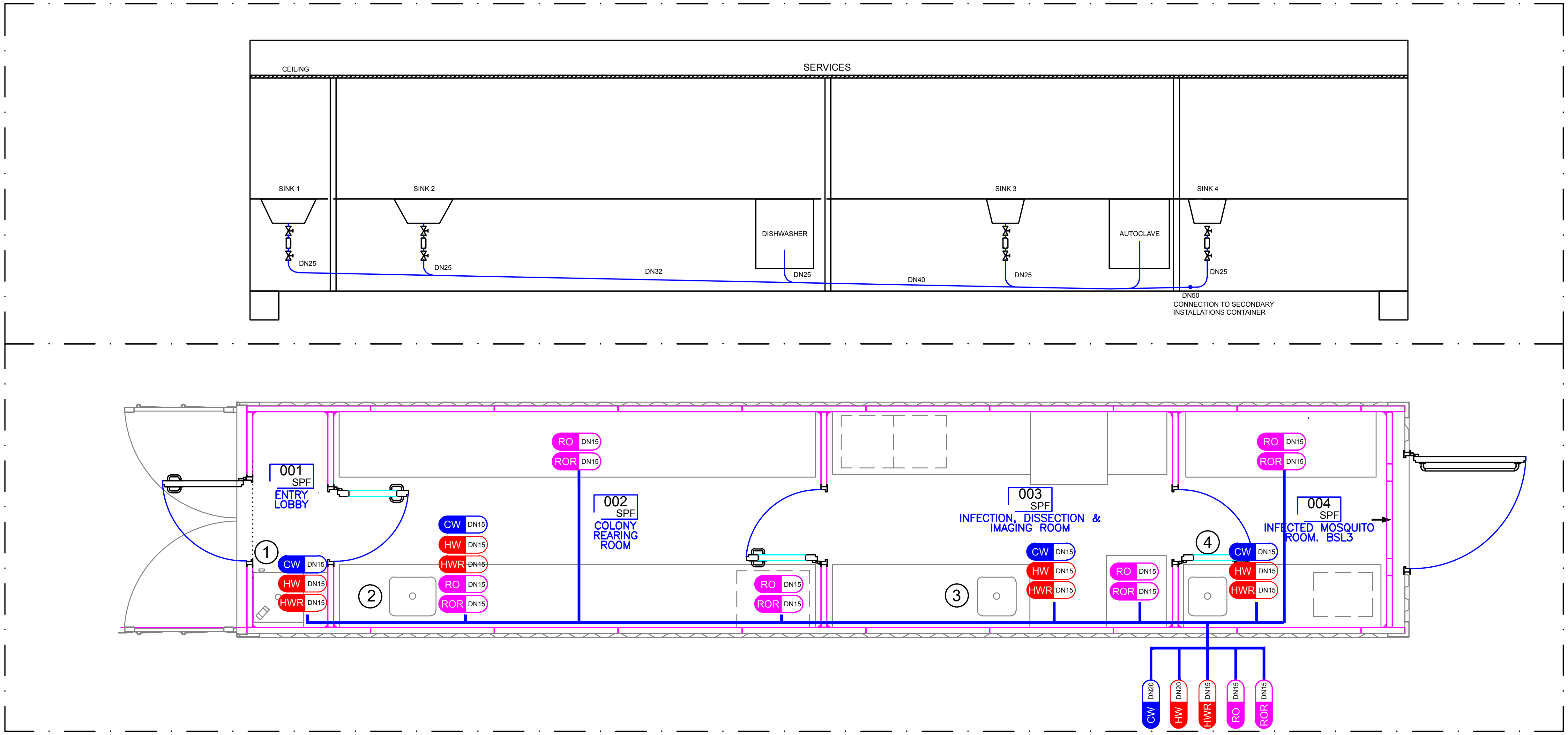

Lay-out
scale 1/50

CODE	PIPE DESCRIPTION	MATERIAL
CW DN20	COLD WATER	Polyethylene HDPE
HW DN20	HOT WATER	Polyethylene HDPE
HWR DN15	RETURN OF HOT WATER	Polyethylene HDPE
RO DN15	SOFTENED & REVERSE OSMOSIS TREATED WATER	Polypropylene PP-H
ROR DN15	RETURN OF SOFTENED & REVERSE OSMOSIS TREATED WATER	Polypropylene PP-H

transmission zero

IMPERIAL

drawing scale 1:50
0 1m 2 3

A3
420x297

project: Modular Portable Laboratory	title: TECHNICAL DESIGN PROJECT PIPES DISTRIBUTION AND DRAINAGE	18	scale 1/50; drawing 15549-PB-TB-01

19-Lighting

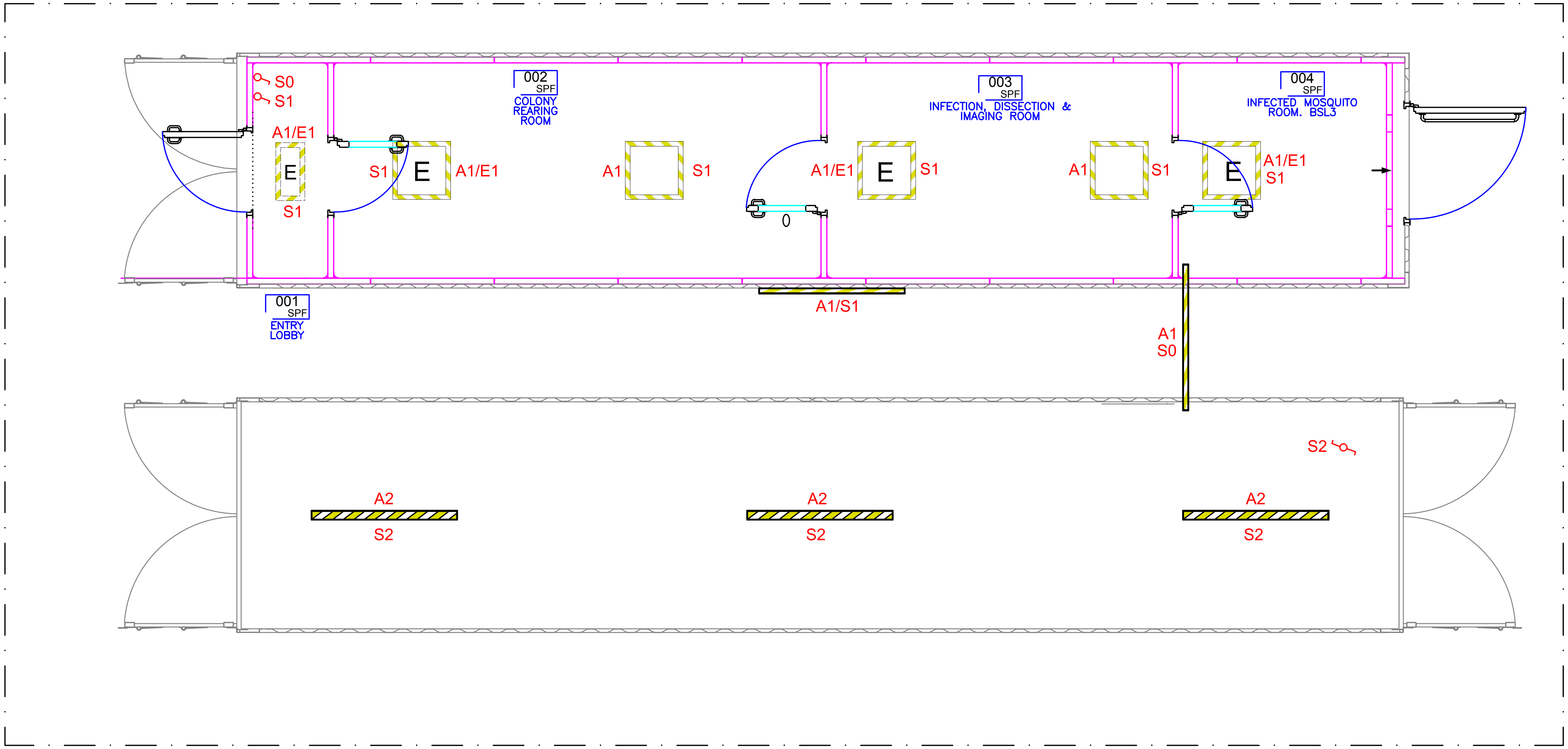

Lay-out

scale 1/50

AX/EX ... SX	AX: LIGHTING CIRCUIT<br>EX: EMERGENCY CIRCUIT<br>SX: ON/OFF SWITCH

note

(airtight lamp symbol)	LED AIRTIGHT LAMP IP65<br>46x83x1,500mm, 60W, 5,400 Lm.
(square lamp symbol)	LED LAMP 600x600 mm ACCESSIBLE FROM BELOW
E	LED LAMP 600x600 mm ACCESSIBLE FROM BELOW<br>WITH EMERGENCY KIT (3 HOURS)
E	LED LAMP 300x600 mm ACCESSIBLE FROM BELOW<br>WITH EMERGENCY KIT (3 HOURS)
(switch symbol)	SIMPLE SWITCH ON/OFF

legend

transmissionzero

IMPERIAL

drawing scale 1:50

0 1m 2 3

A3 420x297

project: Modular Portable Laboratory

TECHNICAL DESIGN PROJECT

title: LIGHTING

19

scale 1/50

drawing 15549-PB-EC-00

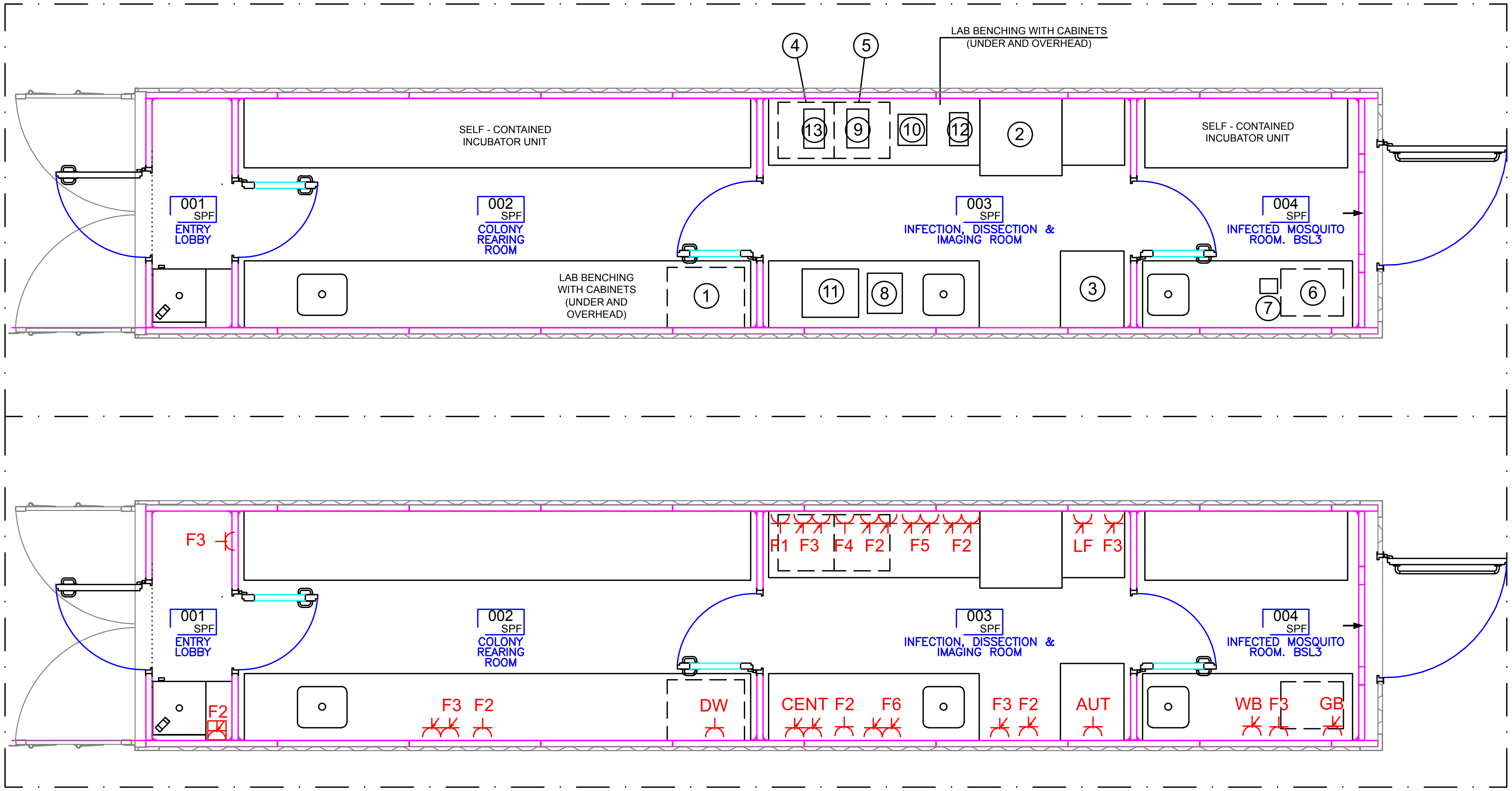

Lay-out

scale 1/50

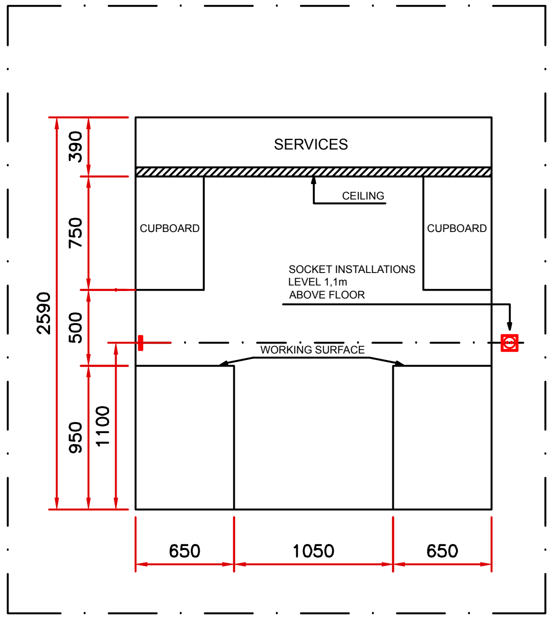

Section

scale 1/50

000 GMP ROOM NAME — ROOM NAME, CLASSIFICATION AND ID. NUMBER

POWER SOCKET TYPE G. LEVEL 1.5m ABOVE FLOOR (hand held air blower)

POWER SOCKET TYPE G. LEVEL 1.1m ABOVE FLOOR

POWER SOCKET TYPE G. LEVEL 0.3m ABOVE FLOOR

legend

DESCRIPTION OF EQUIPMENT

Nº	NAME	WEIGHT (kg)	DIMENSIONS (mm)	HEIGHT (mm)	POWER (kW)
1	DISHWASHER MIELE PG 8504 [AD]	74	600x600	835	9.20 kW
2	CLASS II MICROBIOLOGICAL SAFETY CABINET GUARDIAN MSC800	135	800x750	152	0.15 kW
3	TOUCHCLAVE TCR/40/H1-SPECIAL AUTOCLAVE	120	580x720	710	2.50 kW
4	FRIDGE LIEBHERR TP 1410	35.7	554x623	850	0.19 kW
5	FREEZER LIEBHERR GP 1376	39.3	553x624	851	0.30 kW
6	BENCHTOP GLOVE BOX ELECTRO-TECH 5503 SERIES	31	610x457	381	

Nº	NAME	WEIGHT (kg)	DIMENSIONS (mm)	HEIGHT (mm)	POWER (kW)
7	CIRCULATING BATHS GRANT TC120	2.3	172x141	333	1.40 kW
8	FLUORESCENT STEREO MICROSCOPE LEICA BIOSCIENCE, M205 FA		340x390	547	
9	STANDARD LIGHT MICROSCOPE OLYMPUS CX43	7.3	211x376	393	
10	DISSECTION/ STEREO MICROSCOPE LEICA BIOSYSTEMS M80		278x301	395	
11	T CONTROLLED CENTRIFUGE: EPPENDORF CENTRIFUGE 5804	55	470x550	340	0.90 kW
12	LABORATORY LIQUID SUCTION SYSTEM INTEGRA VACUSAFE	3.4	180x320	530	
13	HEATING BLOCK GRANT QBD4		380x200	100	0.60 kW

20-Power Sockets

transmission zero

IMPERIAL

drawing scale 1:50

0 1m 2 3

A3
420x297

project: Modular Portable Laboratory

TECHNICAL DESIGN PROJECT

title: POWER SOCKETS

20

scale 1/50

drawing 15549-PB-EC-01

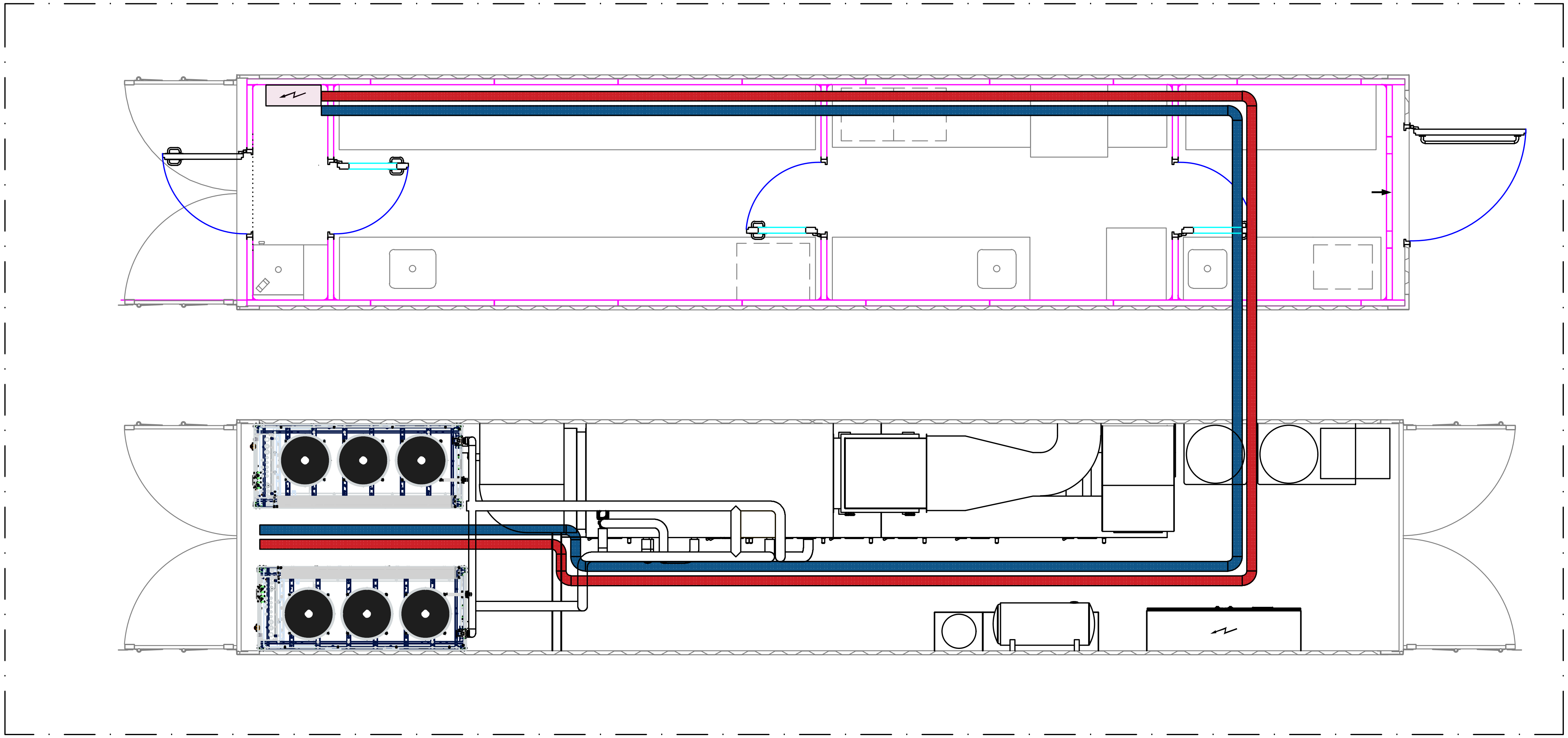

Lay-out
scale 1/50

21-Electrical Channels

transmission zero

IMPERIAL

LIGHTING AND SOCKETS TRAY.
TRAY MESH (REJIBAND TYPE)

CONTROL TRAY.
TRAY MESH (REJIBAND TYPE)

legend

drawing scale 1:50
0 1m 2 3

A3
420x297

project: Modular Portable Laboratory

TECHNICAL DESIGN PROJECT

title: ELECTRICAL CHANNELS

21

scale: 1/50

drawing: 15549-PB-EC-02

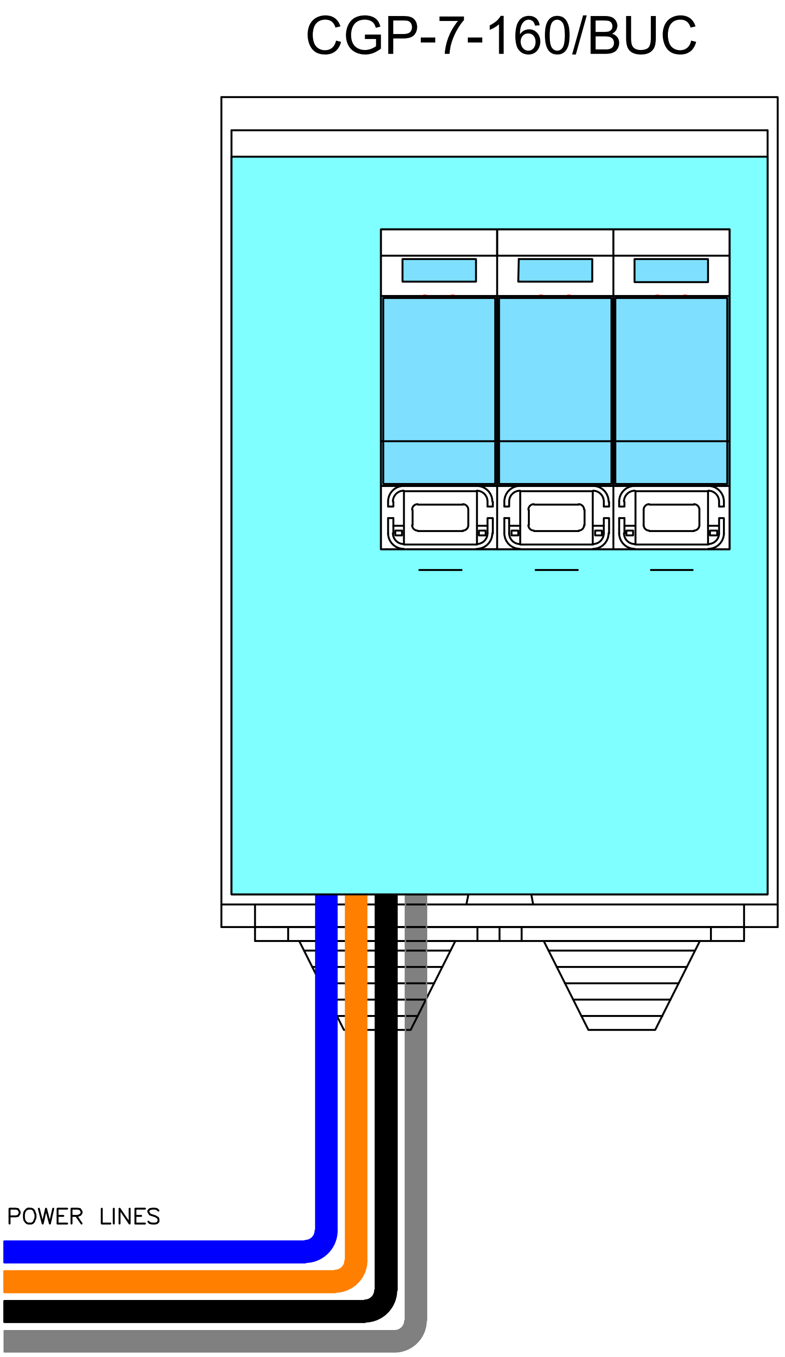

22-Power Income Connection Box

transmission zero

IMPERIAL

Modular Portable Laboratory<br>project	TECHNICAL DESIGN PROJECT<br>POWER INCOME CONNECTION BOX<br>title	22	scale AS INDICATED<br>drawing 15549-PB-EC-03

A4 210x297

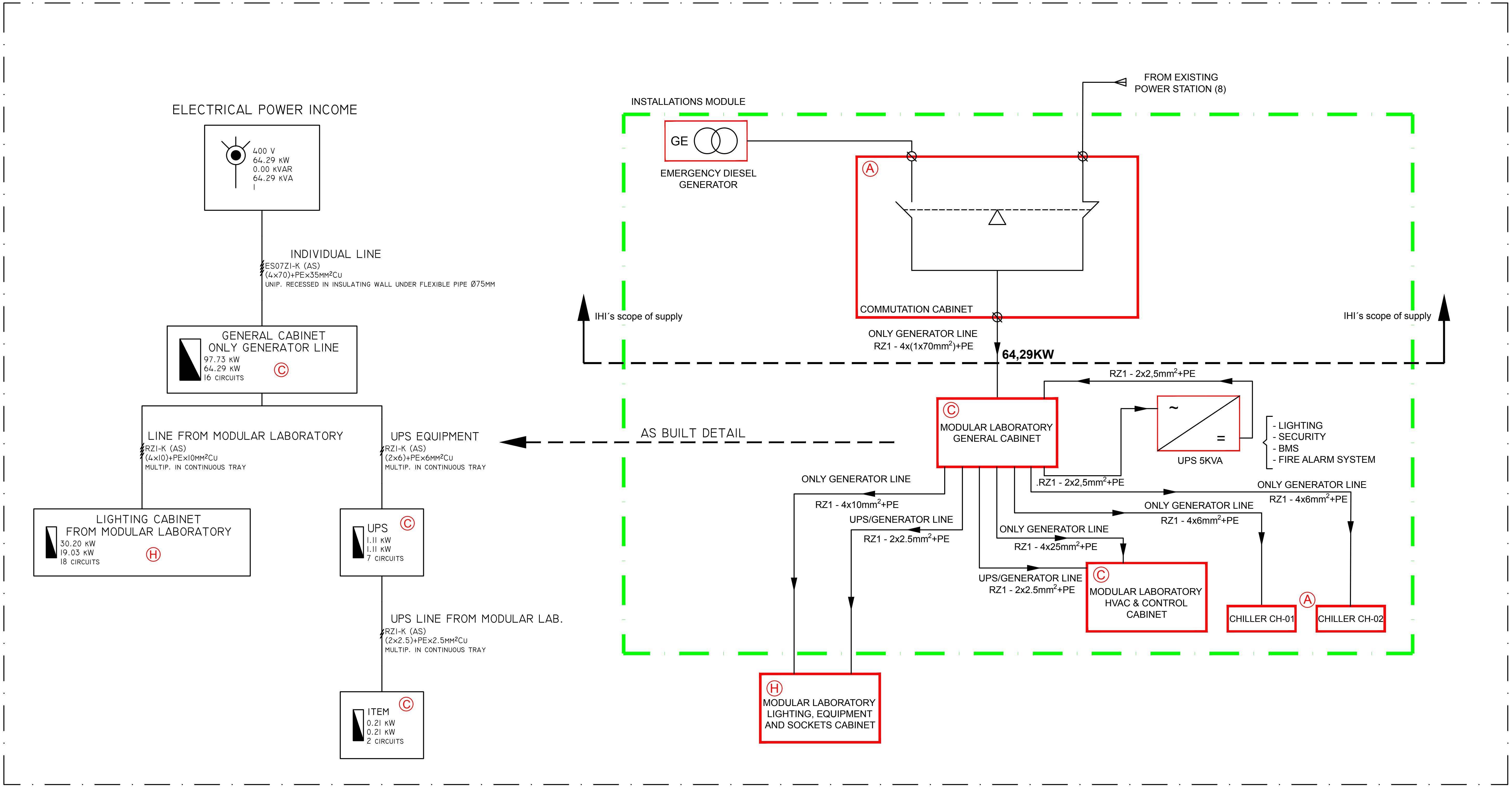

NOTES:

1. UPS POWERED CABINET

23-Electrical Installation General Schema

transmissionzero

IMPERIAL

Modular Portable Laboratory<br>project	TECHNICAL DESIGN PROJECT<br>ELECTRICAL INSTALLATION GENERAL SCHEMA<br>title	23	scale N/S<br>drawing 15549-CJ-EE-00

A3
420x297

24-Modular Laboratory General Cabinet (& HVAC)

ELECTRICAL POWER INCOME
$(4\times70)+PE\times35mm^2Cu$
ES07Z1-K (AS)
tube=75mm

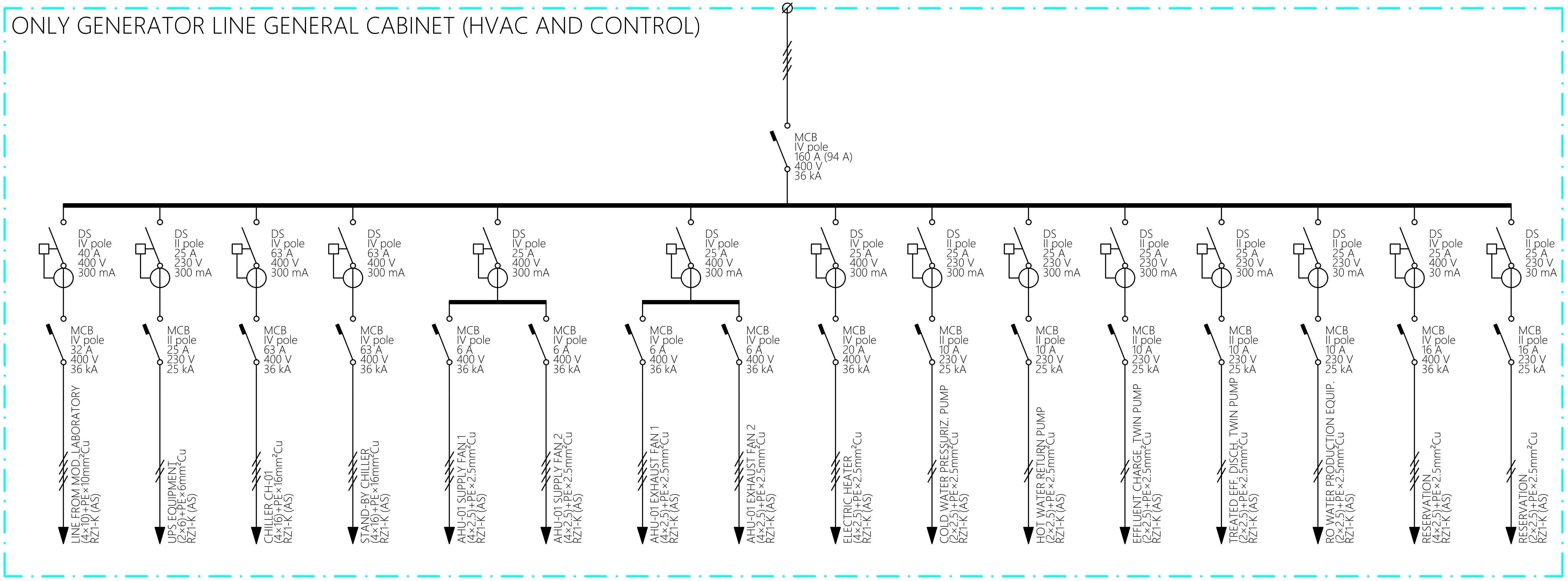

CONSUMPTION	FROM MOD. LAB.	UPS	CHILLER CH-01	STAND-BY CHILLER	SUPPLY FAN 1	SUPPLY FAN 2	EXHAUST FAN 1	EXHAUST FAN 2	ELECTRIC HEATER	COLD WATER PUMP	HOT WATER PUMP	EFFLUENT PUMP 1	EFFLUENT PUMP 2	RO WATER PROD.	RESERVATION	RESERVATION
Pcal (W)	19.026	1.11	22.000	22.000	1.230	1.230	1.230	1.230	9.000	250	250	1.000	1.000	1.000	3.000	2.000
Un (V)	400	230	400	400	400	400	400	400	400	230	230	230	230	230	400	230
Ib (A)	27,46	4,83	31,75	31,75	1,78	1,78	1,78	1,78	12,99	1,09	1,09	4,35	4,35	4,35	4,33	8,70
Iz (A)	64,61	52,78	87,36	87,36	27,30	27,30	27,30	27,30	27,30	30,03	30,03	30,03	30,03	30,03	27,30	30,03
Sf (mm^2)	10	6	16	16	2,5	2,5	2,5	2,5	2,5	2,5	2,5	2,5	2,5	2,5	2,5	2,5
Ltot (m)	10,00	10,00	10,00	10,00	10,00	10,00	10,00	10,00	10,00	10,00	10,00	10,00	10,00	10,00	10,00	10,00
Lvd (m)	10,00	10,00	10,00	10,00	10,00	10,00	10,00	10,00	10,00	10,00	10,00	10,00	10,00	10,00	10,00	10,00
VD,circ. (%)	0,2283	0,1304	0,1636	0,1636	0,0572	0,0572	0,0572	0,0572	0,4356	0,0703	0,0703	0,2822	0,2822	0,2822	0,1401	0,5709
VD,tot. (%)	0,2853	0,1873	0,2206	0,2206	0,1142	0,1142	0,1142	0,1142	0,4925	0,1273	0,1273	0,3392	0,3392	0,3392	0,1970	0,6279

NOTES:

1. DS: DIFFERENTIAL SWITCH; MCB: THERMAL–MAGNETIC (AUTOMATIC) CIRCUIT BREAKER

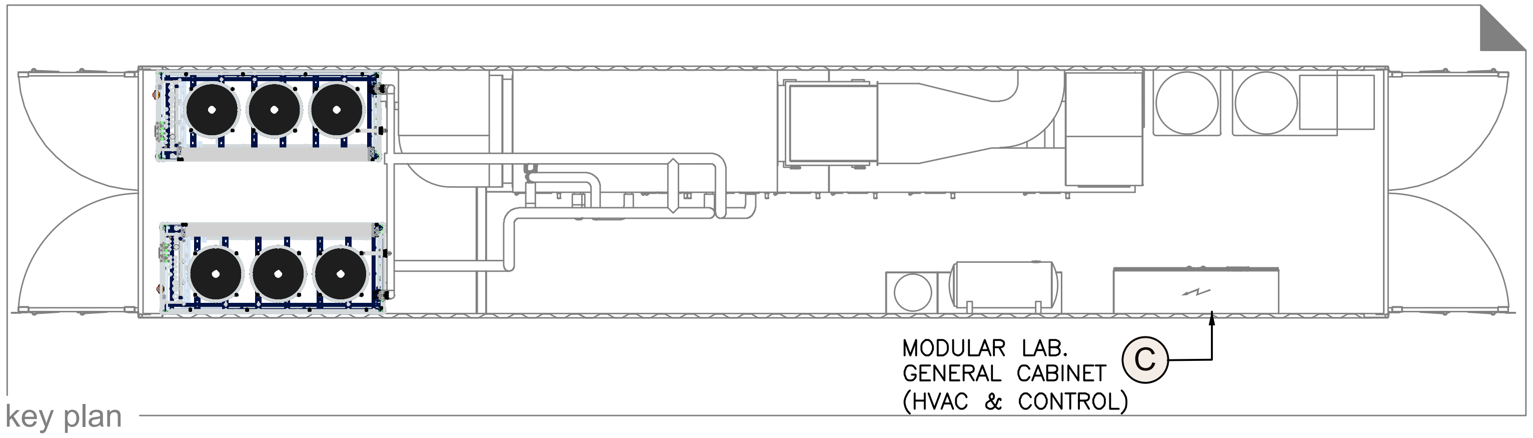

key plan

transmission zero

IMPERIAL

project: Modular Portable Laboratory

title: TECHNICAL DESIGN PROJECT
MODULAR LABORATORY GENERAL CABINET (& HVAC) C 24

scale N/S

drawing 15549-CJ-EE-01

A3 420x297

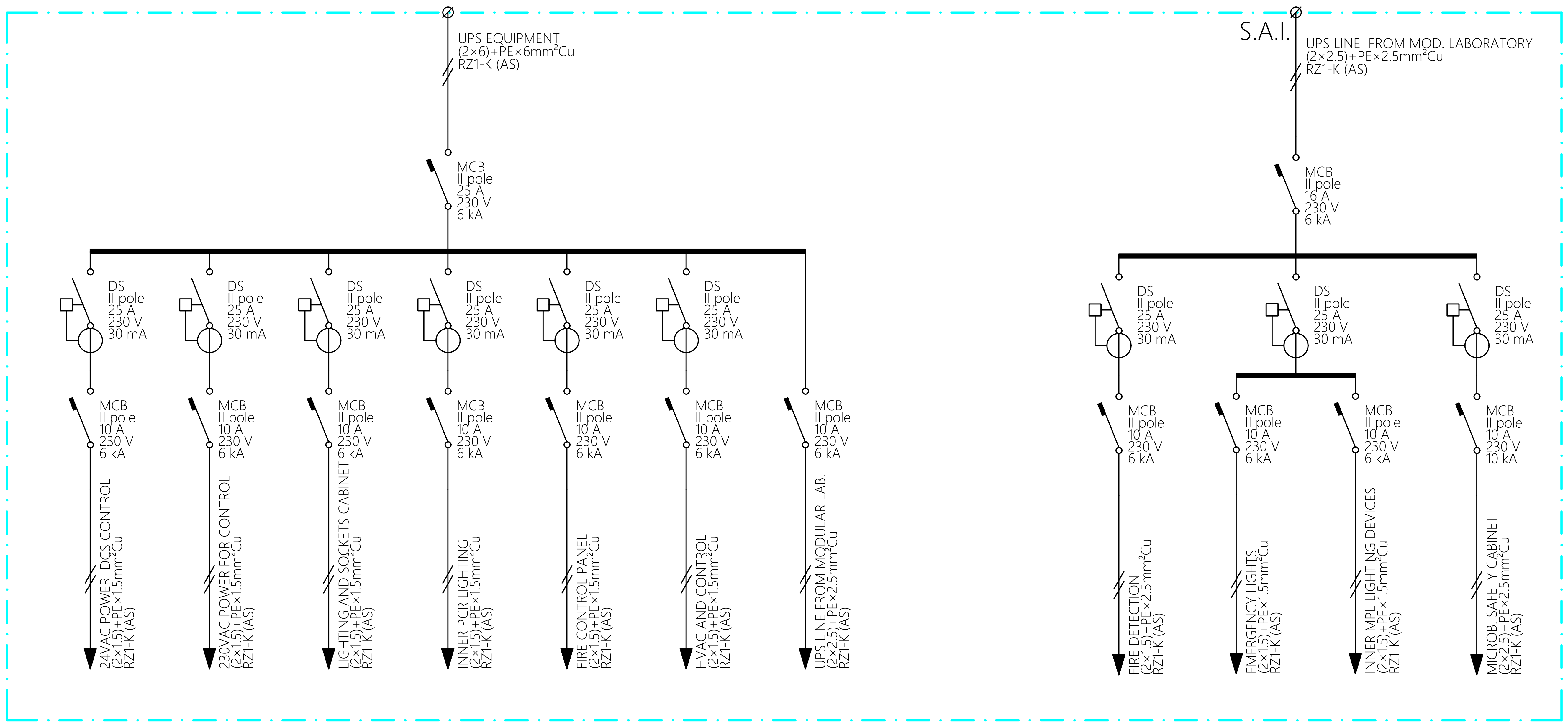

CONSUMPTION	24VAC DCS CONT.	230VAC CONTROL	LIGHTING CABINET	INNER PRC LIGHT.	FIRE PANEL	HVAC & CONTROL	UPS LINE
Pcal (W)	250	150	100	100	150	150	211
Un (V)	230	230	230	230	230	230	230
Ib (A)	1,09	0,65	0,43	0,43	0,65	0,65	0,92
Iz (A)	21,84	21,84	21,84	21,84	21,84	21,84	30,03
Sf (mm²)	1,5	1,5	1,5	1,5	1,5	1,5	2,5
Ltot (m)	10,00	10,00	10,00	10,00	10,00	10,00	10,00
Lvd (m)	10,00	10,00	10,00	10,00	10,00	10,00	10,00
VD,circ. (%)	0,1172	0,0703	0,0469	0,0469	0,0703	0,0703	0,0593
VD,acum. (%)	0,3045	0,2576	0,2342	0,2342	0,2576	0,2576	0,2466

CONSUMPTION	FIRE DETECTION	EMERG. LIGHTS	INNER MPL LIGHT.	MICROB. CABINET
Pcal (W)	483	11	200	100
Un (V)	230	230	230	230
Ib (A)	0,35	0,05	0,87	0,43
Iz (A)	2,1	21,84	21,84	30,03
Sf (mm²)	1,5	1,5	1,5	2,5
Ltot (m)	3,00	8,00	8,00	10,00
Lvd (m)	3,00	8,00	8,00	10,00
VD,circ. (%)	0,820	0,0041	0,0750	0,0281
VD,acum. (%)	0,4318	0,2508	0,3216	0,3134

NOTES:

1. DS: DIFFERENTIAL SWITCH; MCB: THERMAL—MAGNETIC (AUTOMATIC) CIRCUIT BREAKER
2. UPS POWERED CABINET

25-Modular Laboratory General Cabinet (U.P.S.)

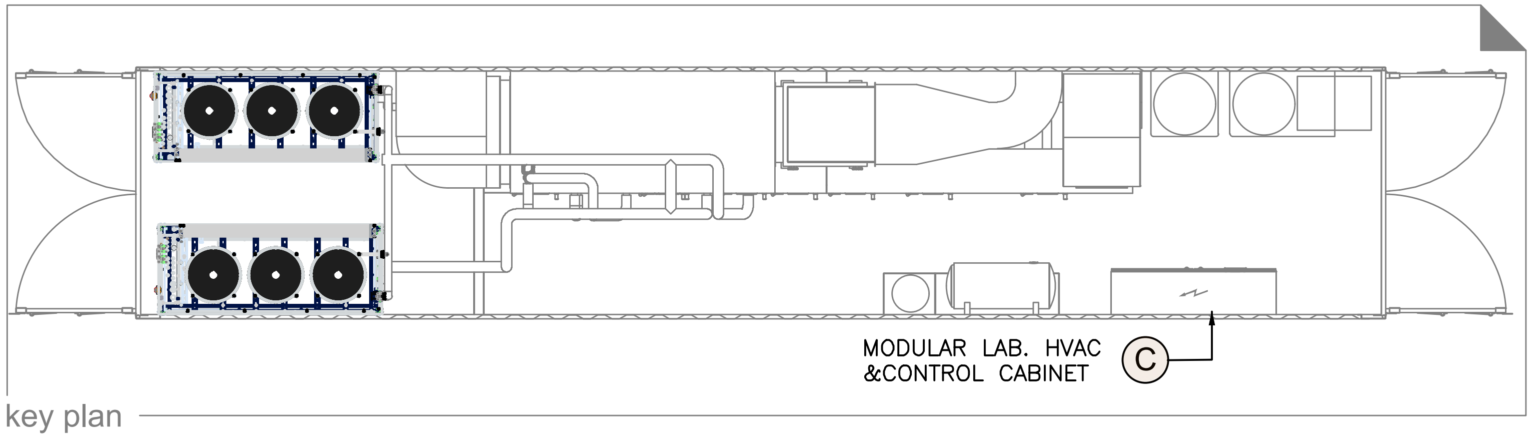

key plan

transmission zero

IMPERIAL

project	title		scale / drawing
Modular Portable Laboratory	TECHNICAL DESIGN PROJECT<br>MODULAR LABORATORY<br>GENERAL CABINET (U.P.S.) Ⓒ	25	scale N/S<br>drawing 15549-CJ-EE-02

A3
420x297

26-Modular Laboratory Lighting, Equipment and Sockets Cabinet

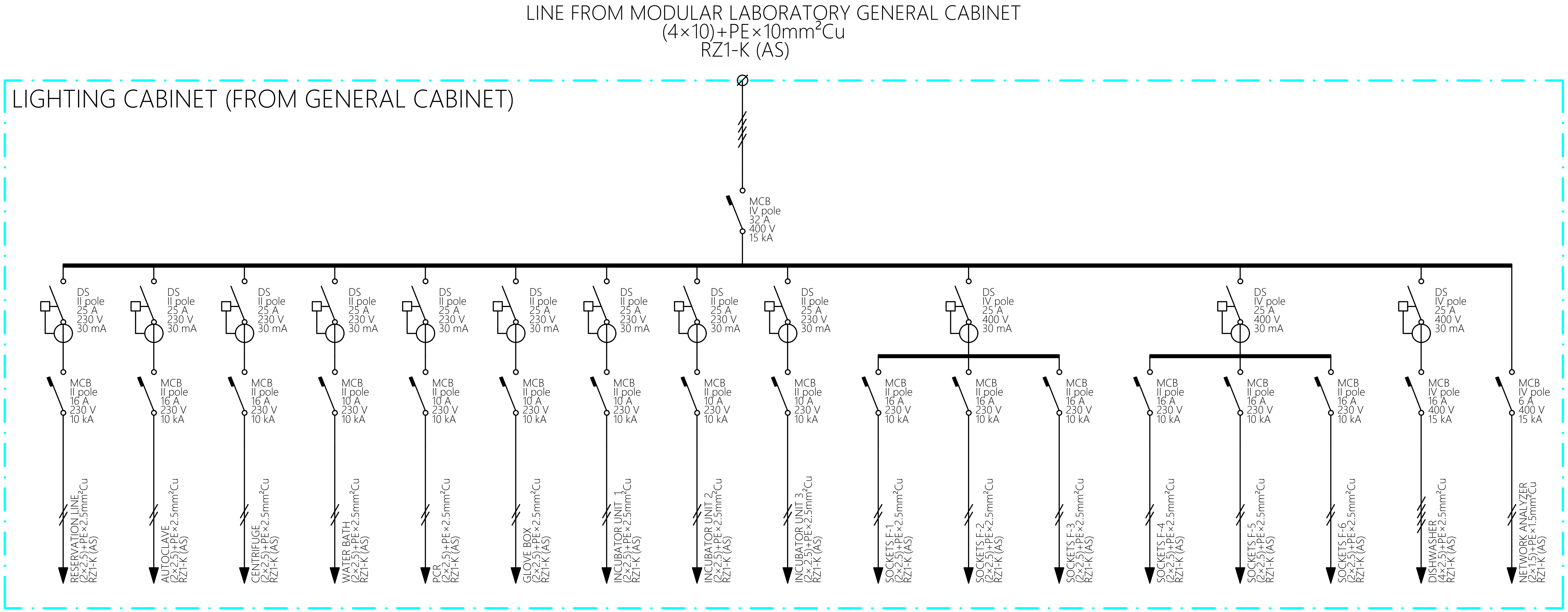

CONSUMPTION	RESERVATION	AUTOCLAVE	CENTRIFUGE	WATER BATH	PCR MACHINE	GLOVE BOX	INCUBATOR 1	INCUBATOR 2	INCUBATOR 3
Pcal (W)	1.200	2.500	900	1.400	600	1.000	2.000	2.000	2.000
Un (V)	230	230	230	230	230	230	230	230	230
Ib (A)	5,22	10,87	3,91	6,09	2,61	4,35	8,70	8,70	8,70
Iz (A)	30,03	30,03	30,03	30,03	30,03	30,03	30,03	30,03	30,03
Sf (mm²)	2,5	2,5	2,5	2,5	2,5	2,5	2,5	2,5	2,5
Ltot (m)	10,00	10,00	10,00	10,00	10,00	10,00	10,00	10,00	10,00
Lvd (m)	10,00	10,00	10,00	10,00	10,00	10,00	10,00	10,00	10,00
VD,circ. (%)	0,3393	0,7197	0,2538	0,3966	0,1689	0,2822	0,5709	0,5709	0,5709
VD,tot. (%)	0,6246	1,0050	0,5391	0,6819	0,4542	0,5675	0,8562	0,8562	0,8562

CONSUMPTION	SOCKETS F-1	SOCKETS F-2	SOCKETS F-3	SOCKETS F-4	SOCKETS F-5	SOCKETS F-6	DISHWASHER	NET. ANALYZER
Pcal (W)	1.200	1.200	1.200	1.200	1.200	1.200	9.200	100
Un (V)	230	230	230	230	230	230	400	230
Ib (A)	5,22	5,22	5,22	5,22	5,22	5,22	13,28	0,43
Iz (A)	30,03	30,03	30,03	30,03	30,03	30,03	27,30	21,84
Sf (mm²)	2,5	2,5	2,5	2,5	2,5	2,5	2,5	1,5
Ltot (m)	10,00	10,00	10,00	10,00	10,00	10,00	10,00	10,00
Lvd (m)	10,00	10,00	10,00	10,00	10,00	10,00	10,00	10,00
VD,circ. (%)	0,3393	0,3393	0,3393	0,3393	0,3393	0,3393	0,4460	0,0469
VD,tot. (%)	0,6246	0,6246	0,6246	0,6246	0,6246	0,6246	0,7313	0,3322

NOTES:

1. DS: DIFFERENTIAL SWITCH; MCB: THERMAL–MAGNETIC (AUTOMATIC) CIRCUIT BREAKER
2. UPS POWERED CABINET

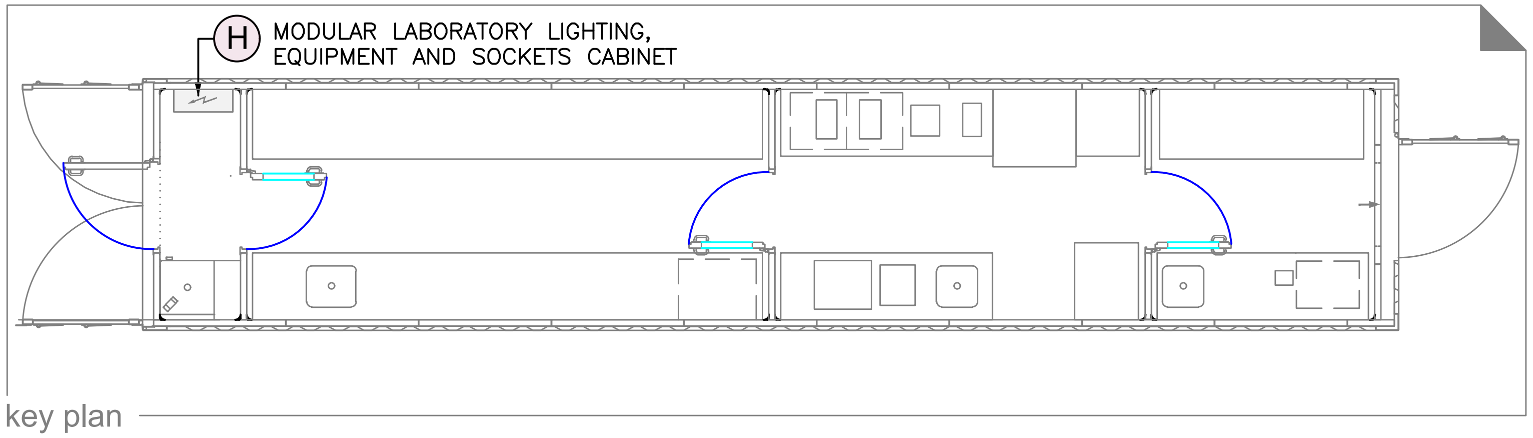

key plan

transmissionzero

IMPERIAL

project	Modular Portable Laboratory
title	TECHNICAL DESIGN PROJECT<br>MODULAR LABORATORY LIGHTING, EQUIPMENT AND SOCKETS CABINET (H) 26
scale	N/S
drawing	15549-CJ-EE-03

A3
420x297

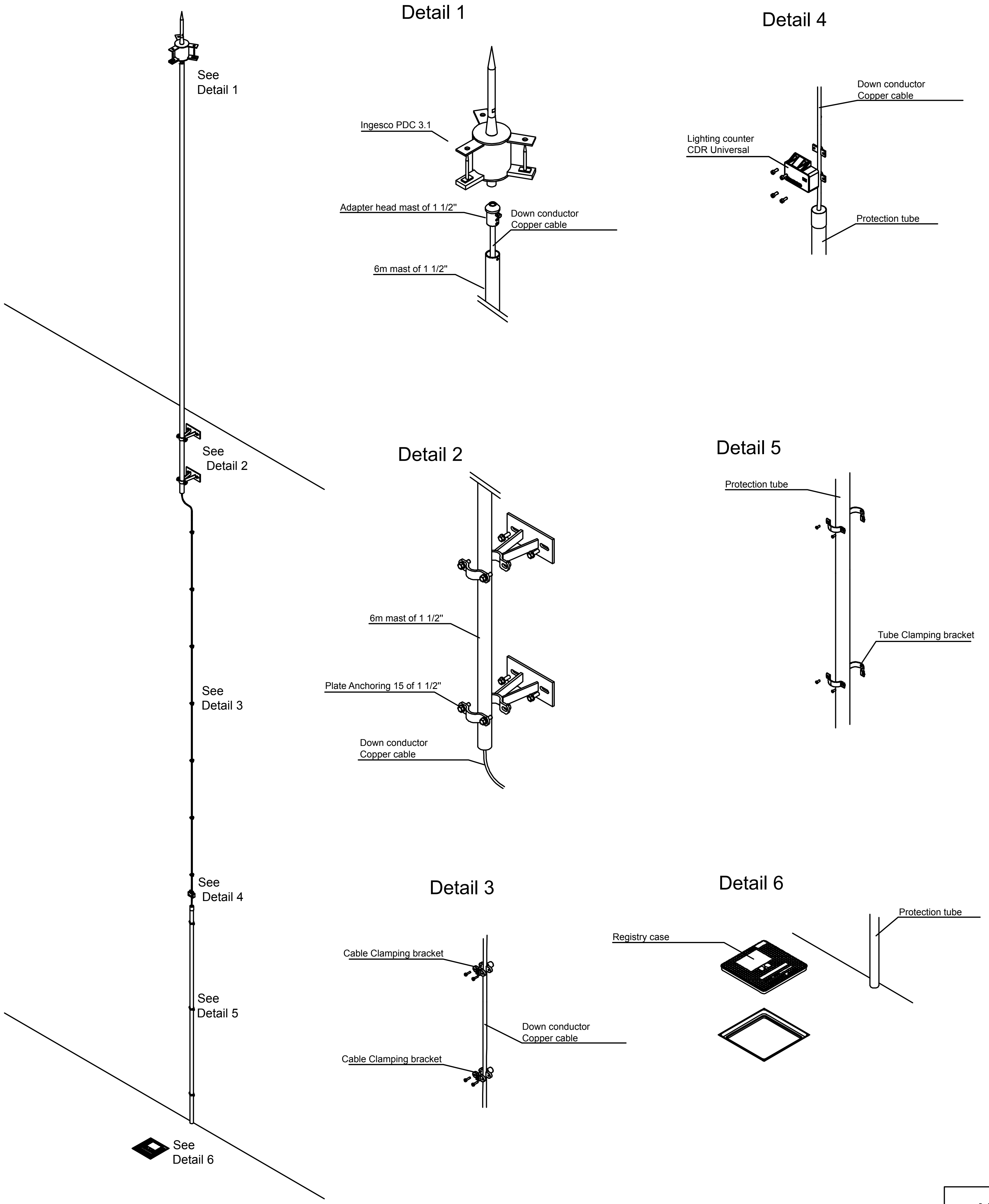

27-Lightning Protection Details

transmissionzero

IMPERIAL

project: Modular Portable Laboratory	title: TECHNICAL DESIGN PROJECT<br>LIGHTNING PROTECTION DETAILS	27	scale: N/S<br>drawing: 15549-CJ-EE-04

A3
420x297

All rights reserved. The use or the total or partial reproduction of this document or its dissemination to third parties without express written permission is prohibited.

28-Magnehelic Plate

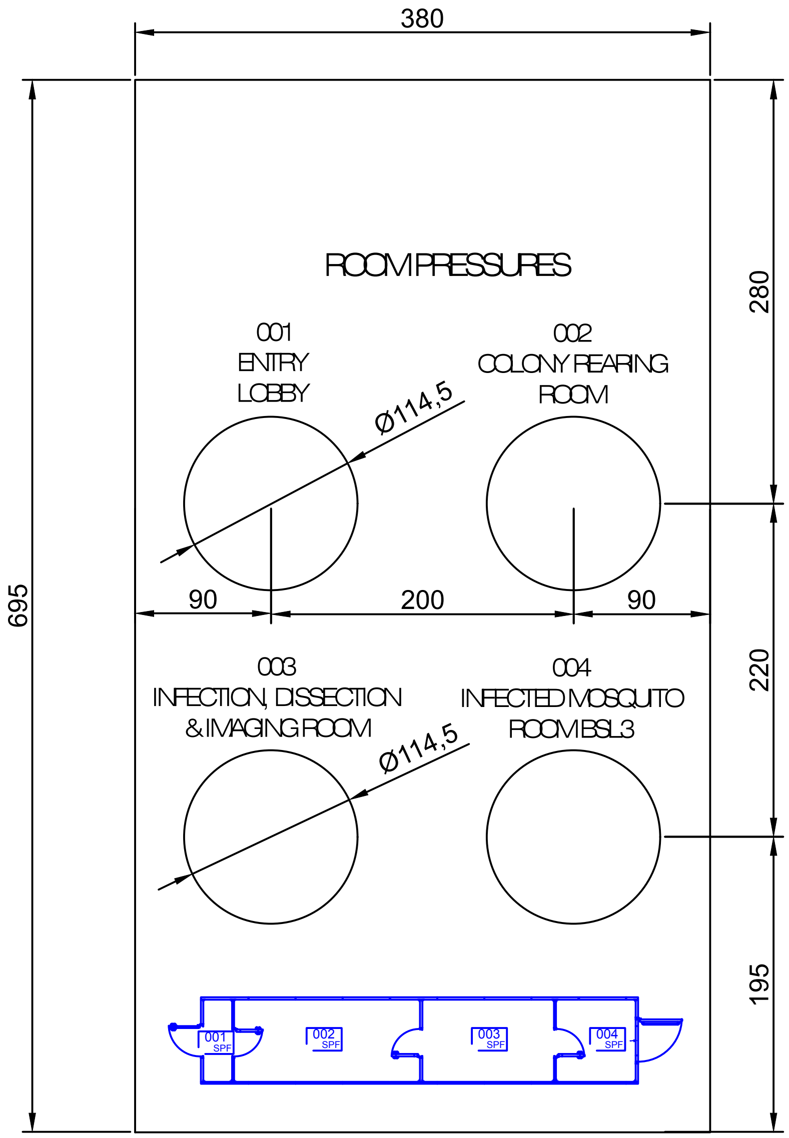

NOTES:

1. REMOVABLE PANEL WITH SCREWS AND RUBBER GASKET.

transmission zero

IMPERIAL

project: Modular Portable Laboratory	title: TECHNICAL DESIGN PROJECT MAGNEHELIC PLATE	28	scale 1:5<br>drawing 15549-DE-00

A4 210x297

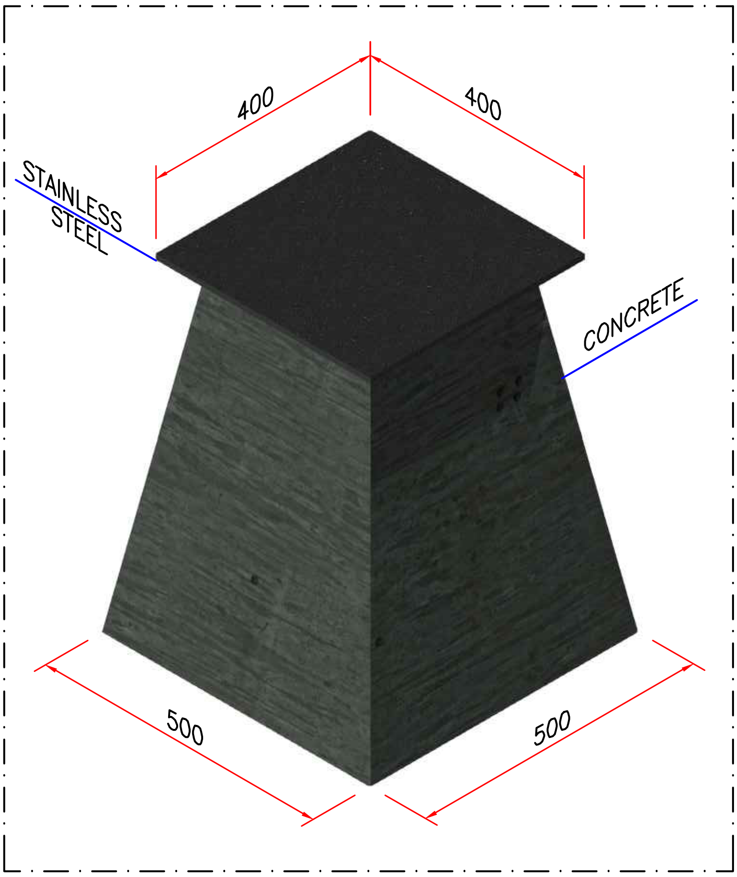

Isometric view
scale 1/10

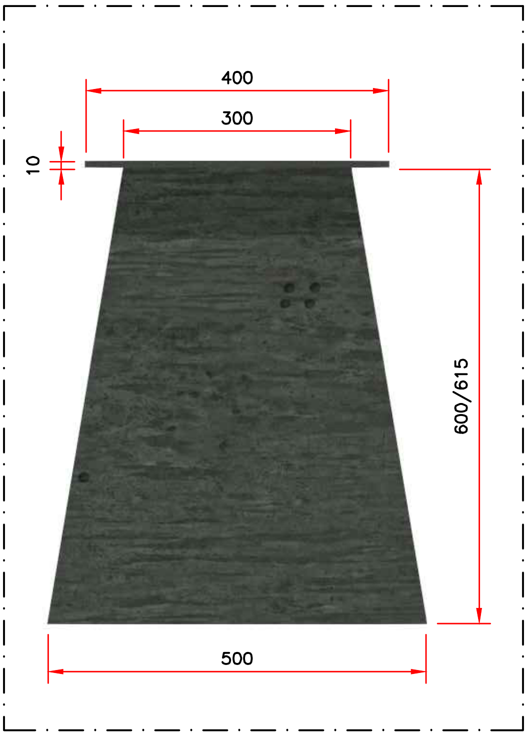

Front view
scale 1/10

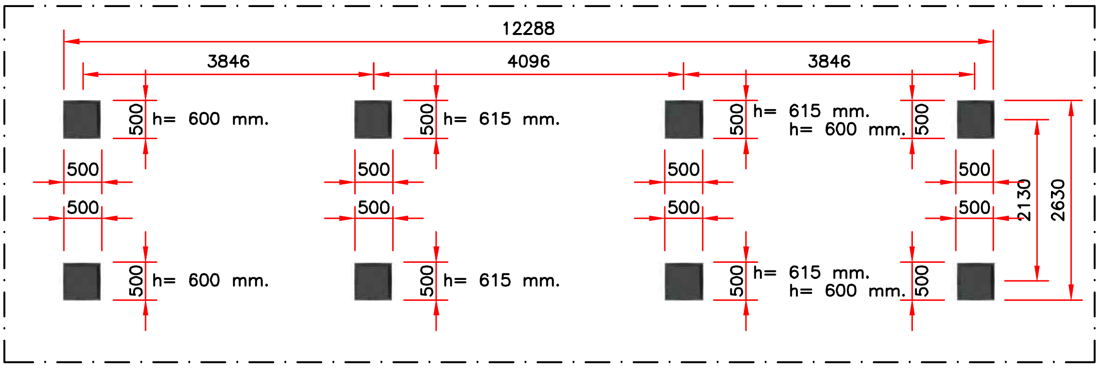

Top view
scale 1/100

1. EIGHT UNITS.

notes

29-MPL Support

transmission zero

IMPERIAL

project	title		scale / drawing
Modular Portable Laboratory	TECHNICAL DESIGN PROJECT<br>MPL SUPPORT	29	scale AS INDICATED<br>drawing 15549-DE-01

A4
210x297

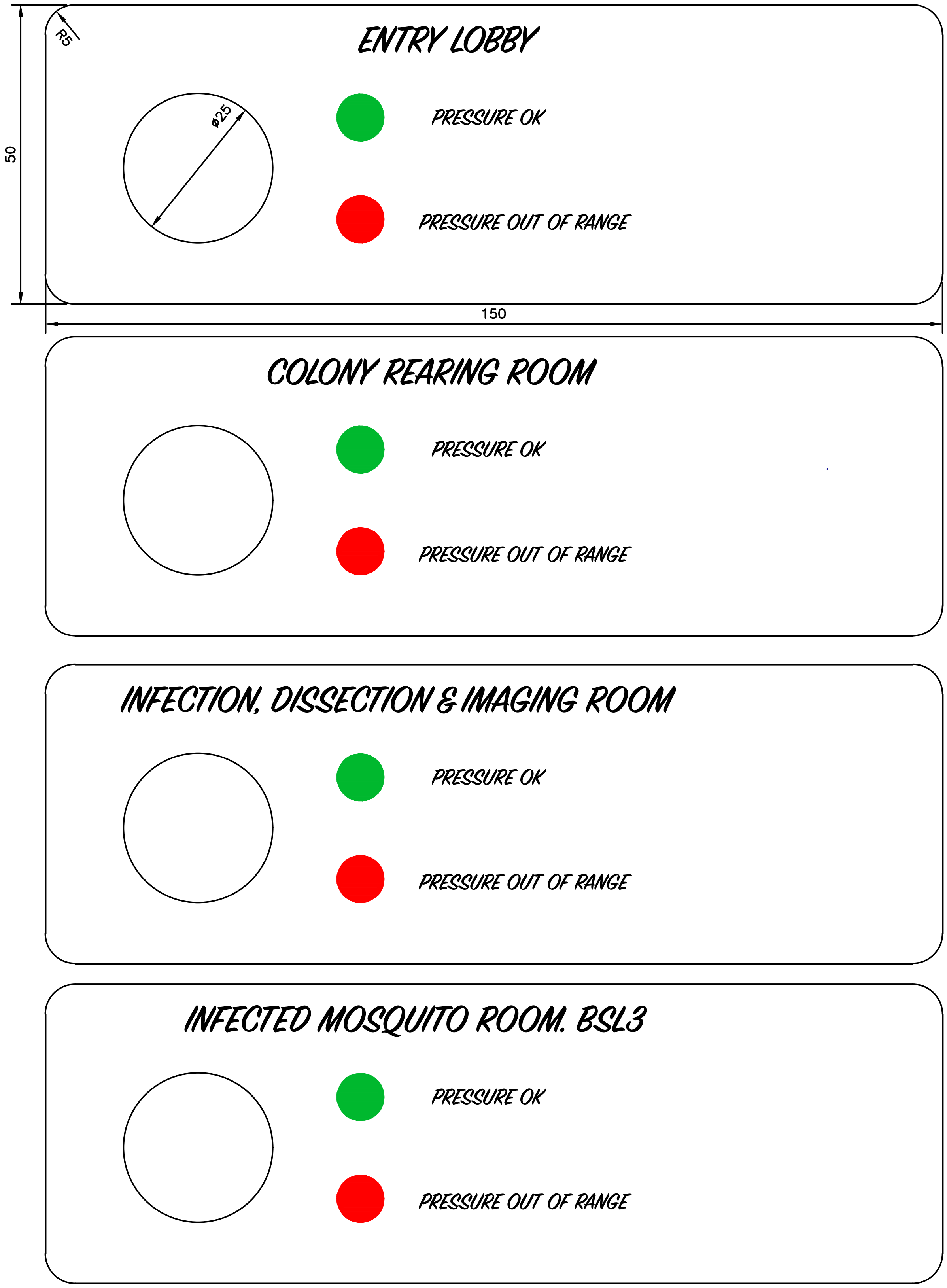

30-Pressure Plates

transmission zero

IMPERIAL

project	title		scale / drawing
Modular Portable Laboratory	TECHNICAL DESIGN PROJECT<br>PRESSURE PLATES	30	scale 1:1<br>drawing 15549-DE-02

A4 210x297

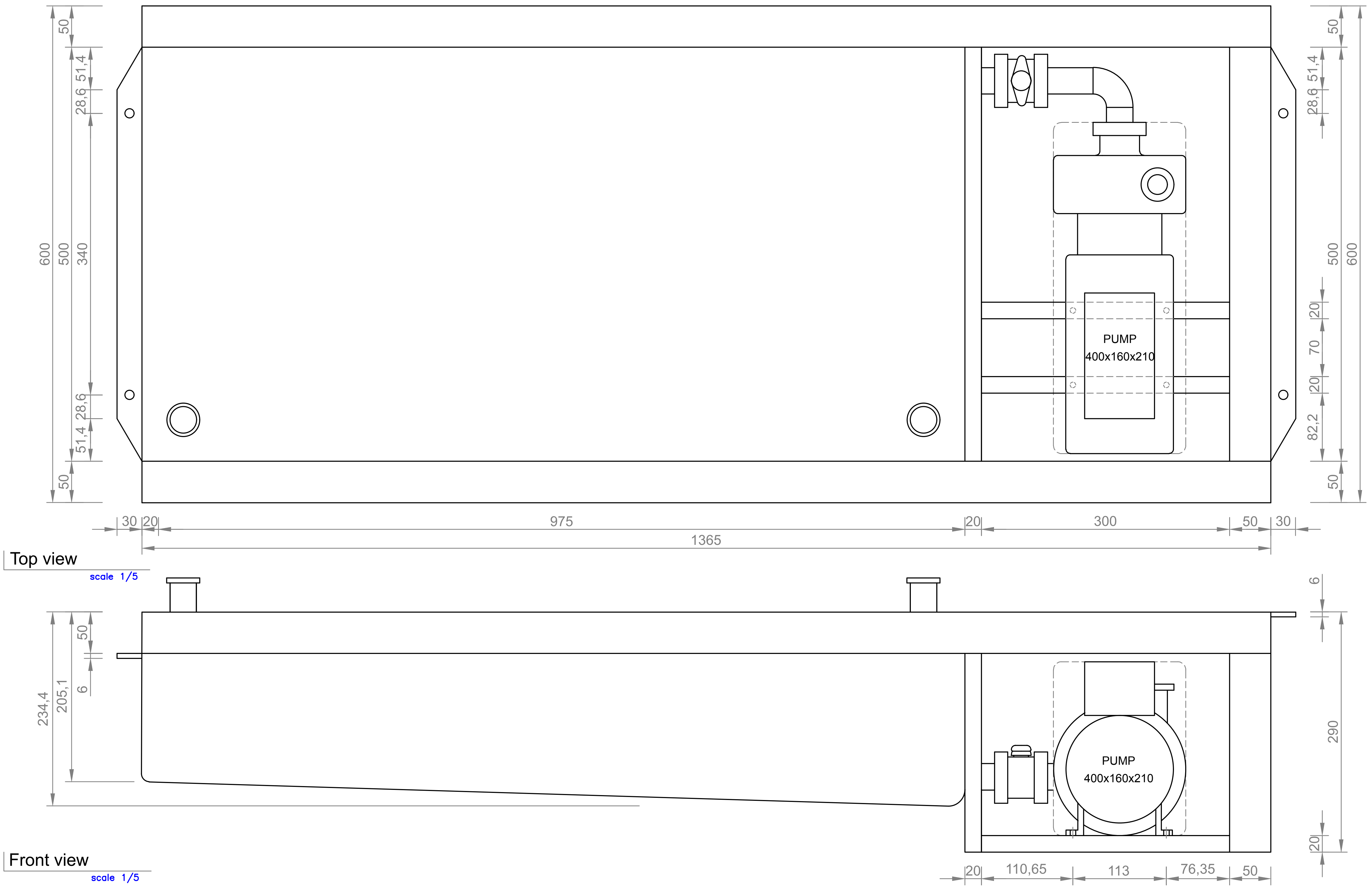

Units in milimeters

31-Reception Tank & Pump

Modular Portable Laboratory<br>project	TECHNICAL DESIGN PROJECT<br>RECEPTION TANK & PUMP<br>title	31	scale 1:5<br>drawing 15549-DE-03

transmission zero

IMPERIAL

A3
420x297

32-Connection Holes & Registry Cases Location

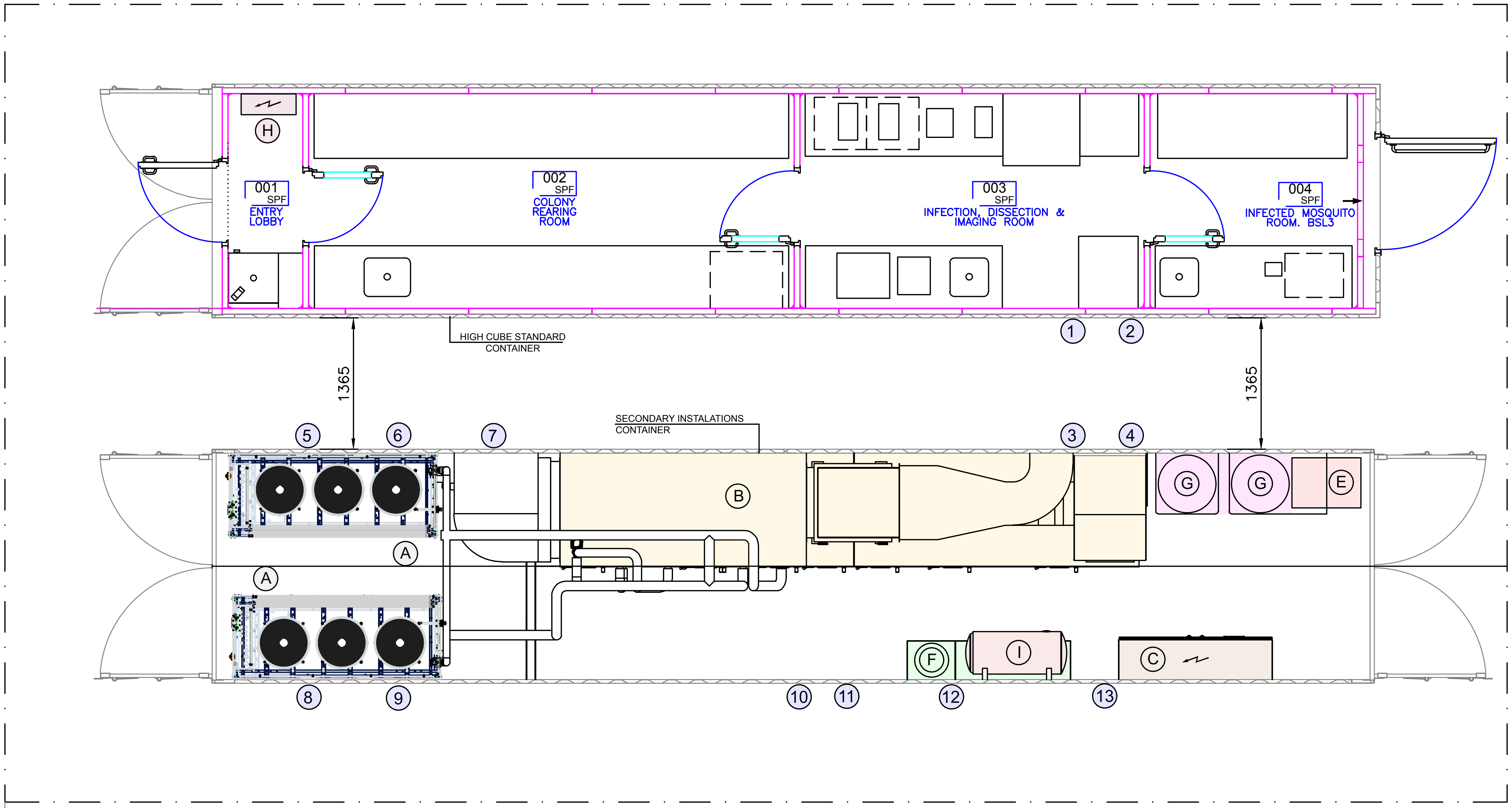

Lay-out

scale 1/50

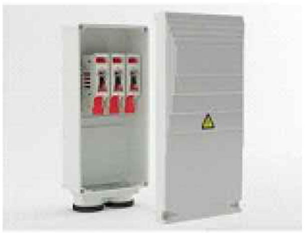

IMAGE 1 - GCP 160 A

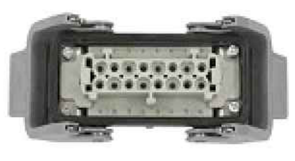

IMAGE 2 - FEMALE HARTING

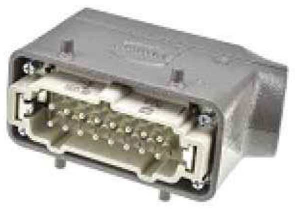

IMAGE 3 - MALE HARTING

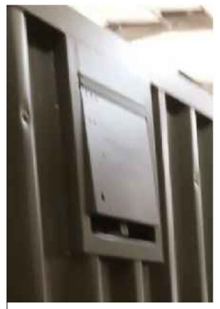

IMAGE 4 - CONNECT. HOLE (REGISTRY CASE)

legend

A	RUN & STANDBY CHILLERS	F	CWS TANK AND BOOSTER SET
B	AIR HANDLING UNIT	G	EFFLUENT TREATMENT PLANT
C	ELECTRICAL GENERAL & HVAC CABINET	H	LIGHTING, EQUIPMENT AND SOCKETS CABINET
E	RO WATER PRODUCTION & STORAGE SYSTEM	I	HOT WATER TANK
		X	HOLE NUMBER

legend

1	DUCT	8	CHILLER
2	ELECTRIC	9	CHILLER
3	DUCT	10	DRAINAGE
4	ELECTRIC	11	DRAINAGE
5	CHILLER	12	WATER
6	CHILLER	13	ELECTRIC
7	AHU		

transmission zero

IMPERIAL

NOTES: SEE DRAWINGS 38 AND 39

drawing scale 1:50

0 1m 2 3

project: Modular Portable Laboratory

TECHNICAL DESIGN PROJECT

title: CONNECTION HOLES & REGISTRY CASES LOCATION

32

scale 1/50

drawing 15549-DE-04

A3 420x297

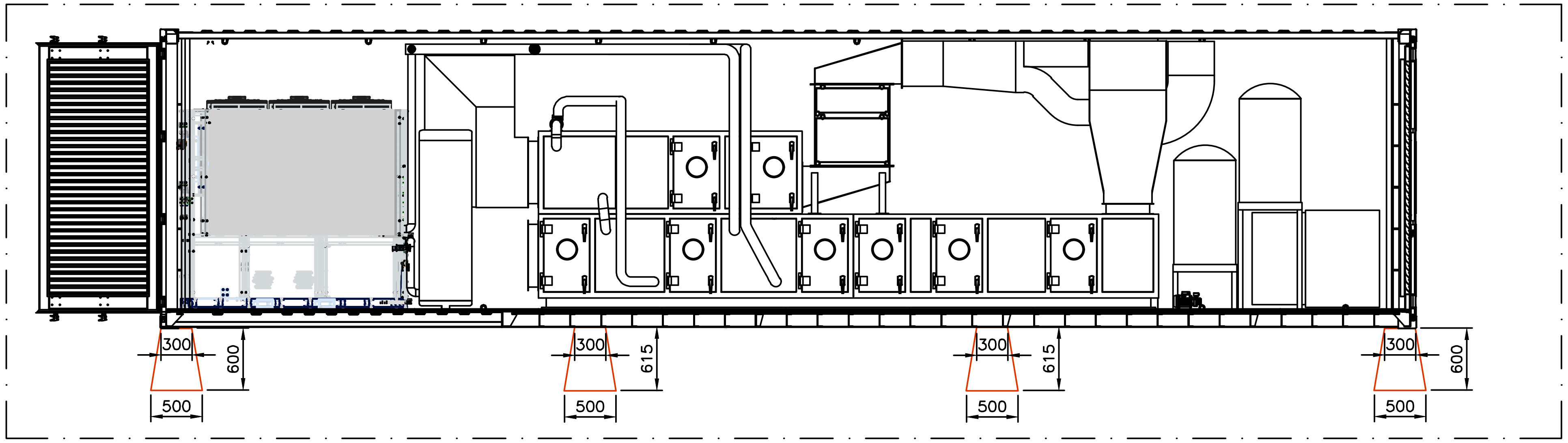

SECTION A-A

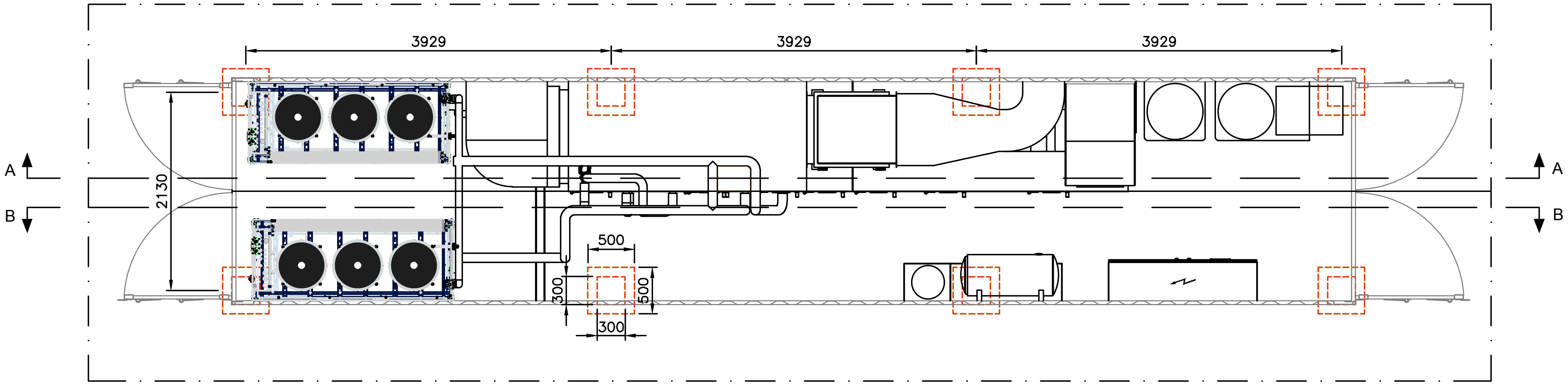

TOP VIEW

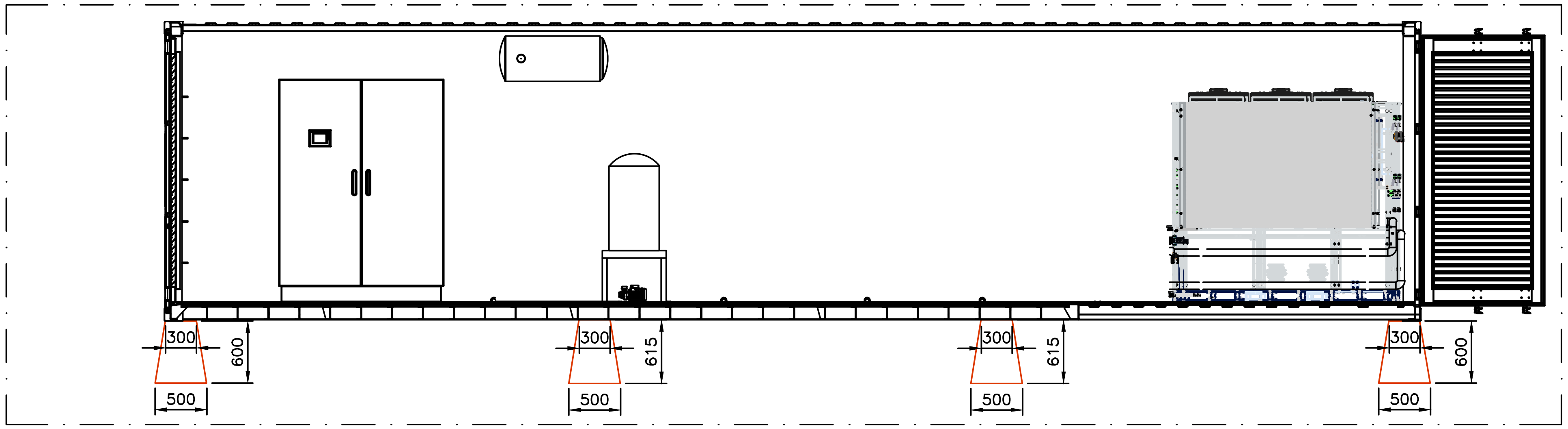

SECTION B-B

transmissionzero

IMPERIAL

33-PCR Module Foundation

drawing scale 1:50

0 1m 2 3

B3
500x353

project	title		scale / drawing
Modular Portable Laboratory	TECHNICAL DESIGN PROJECT<br>PRC MODULE FOUNDATION	33	scale 1/50<br>drawing 15549-DE-05

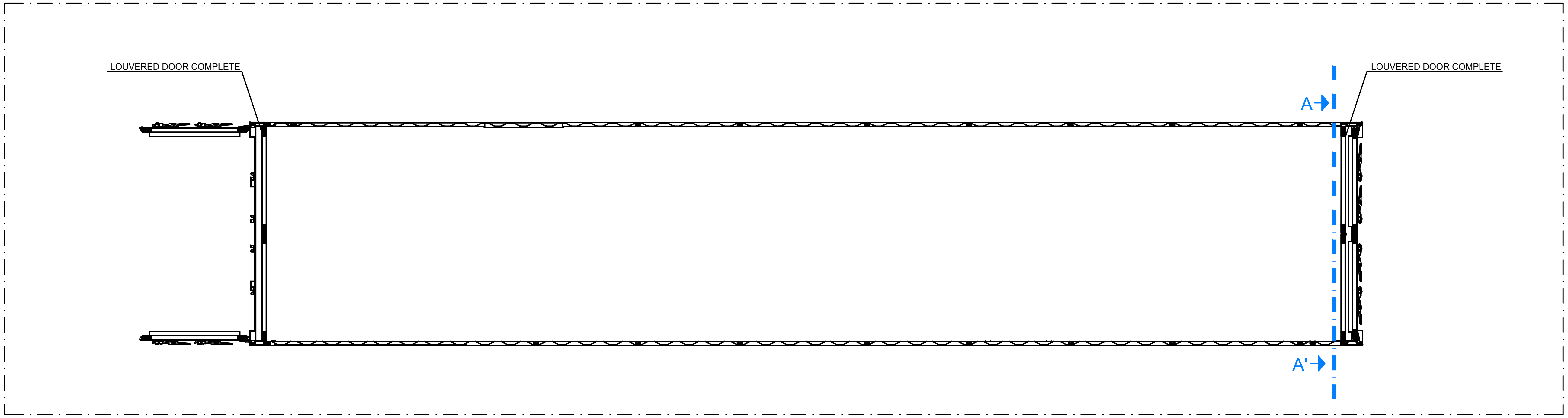

Top view
scale 1/50

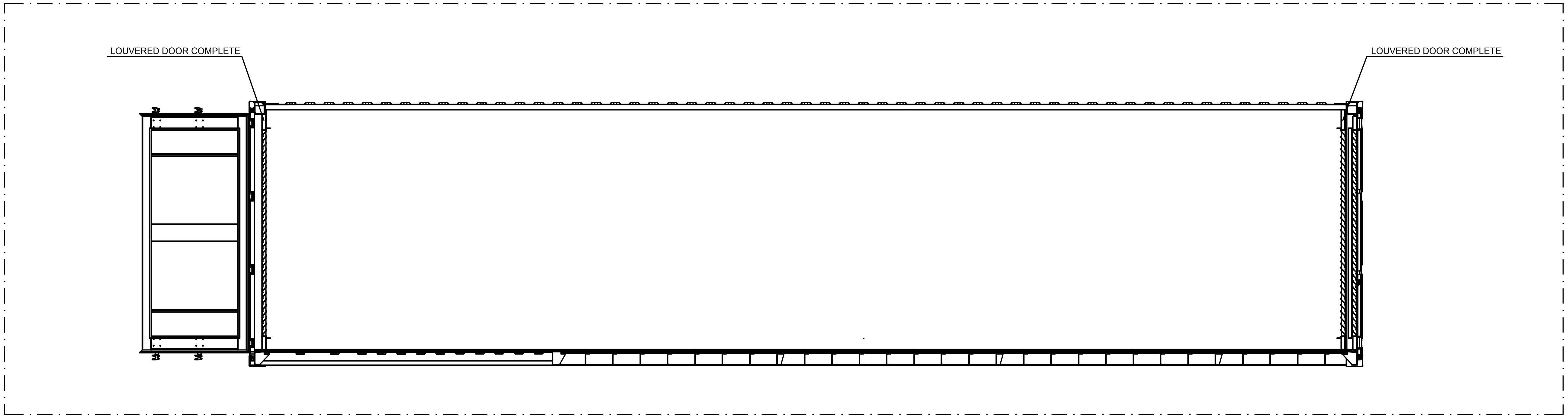

Front view
scale 1/50

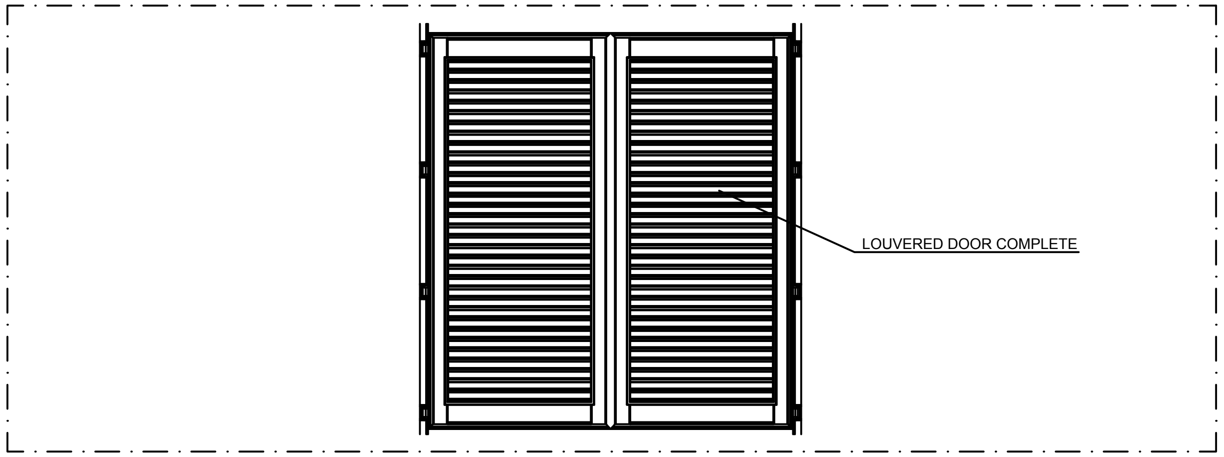

A-A' view-louvered door complete
scale 1/50

34-PCR Module Doors Details

transmissionzero

IMPERIAL

project	title		scale / drawing
Modular Portable Laboratory	TECHNICAL DESIGN PROJECT<br>PCR MODULE<br>DOORS DETAIL	34	scale 1:50<br>drawing 15549-DE-06

A3
420x297

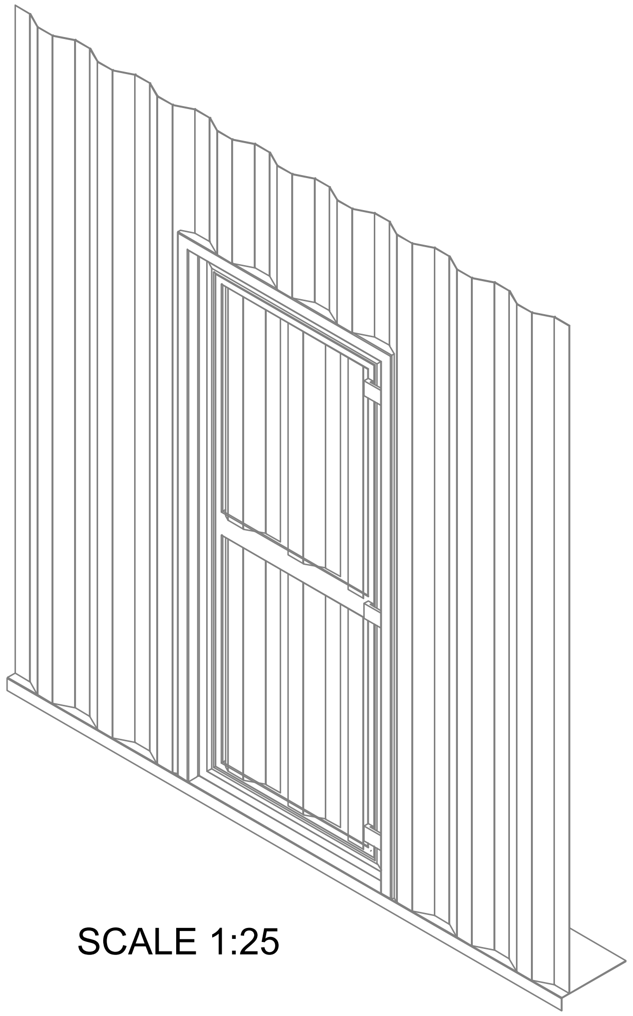

SCALE 1:25

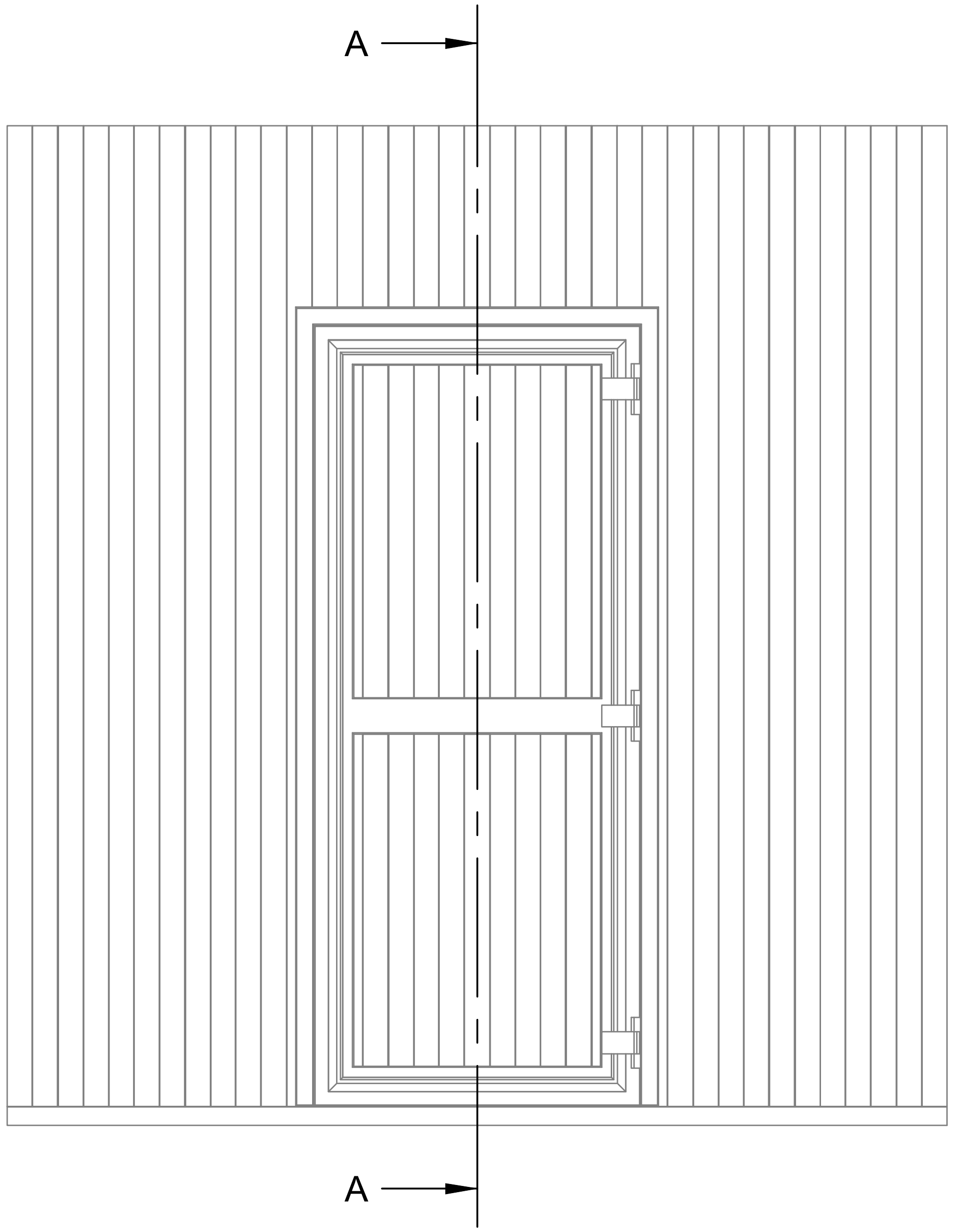

SCALE 1:25

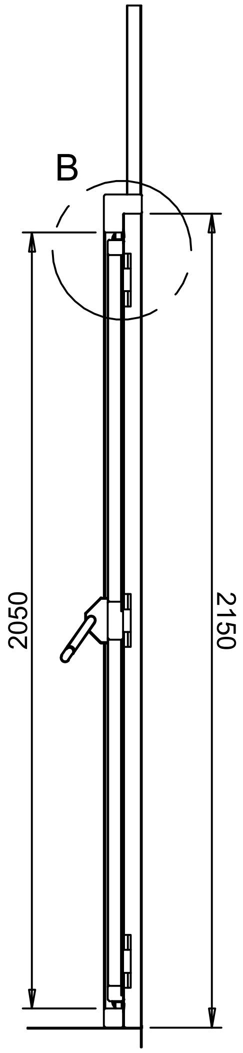

CUT A-A
SCALE 1:25

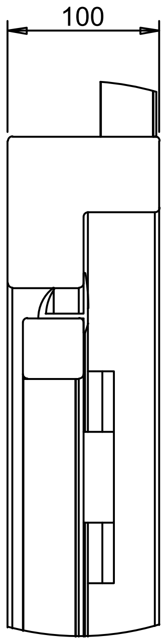

DETAIL B
SCALE 1:5

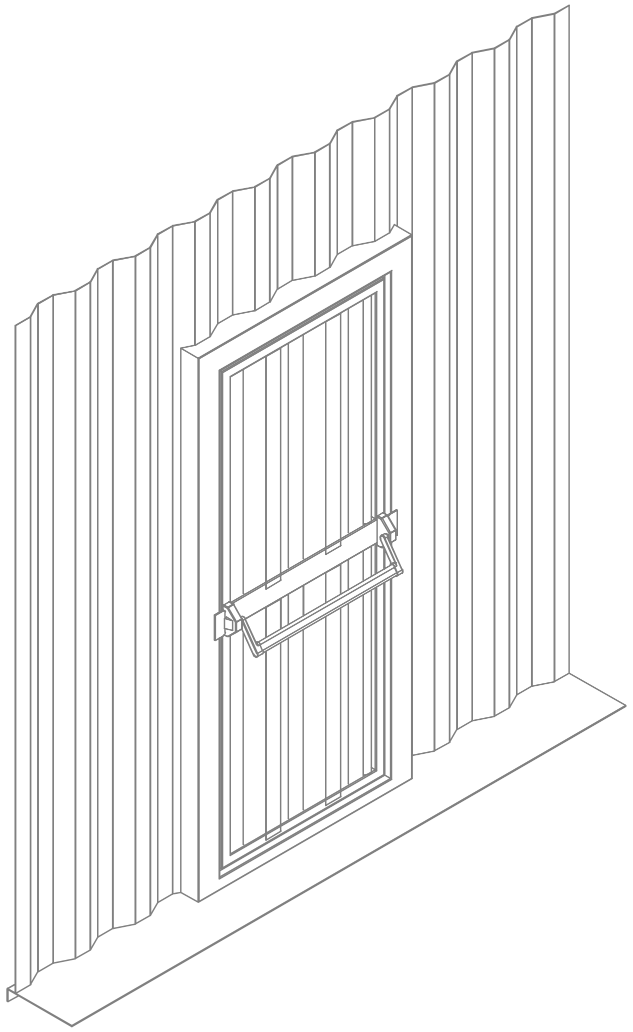

SCALE 1:25

35-Emergency Door Closure Detail

transmission zero

IMPERIAL

Modular Portable Laboratory<br>project	TECHNICAL DESIGN PROJECT<br>EMERGENCY DOOR CLOSURE DETAIL<br>title	35	scale AS INDICATED	drawing 15549-DE-07

A3
420x297

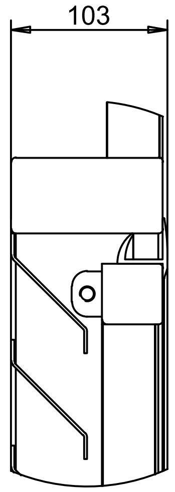

DETAIL B
SCALE 1:5

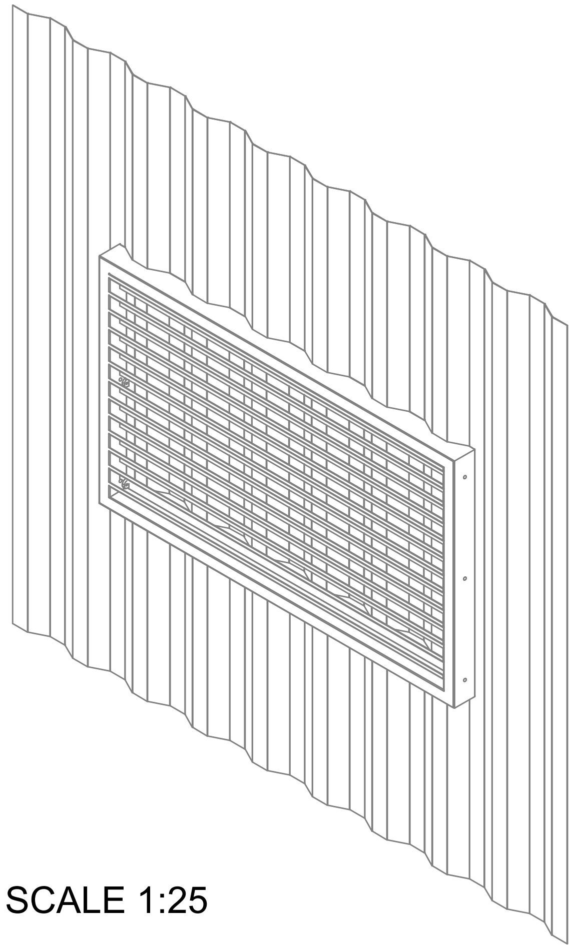

SCALE 1:25

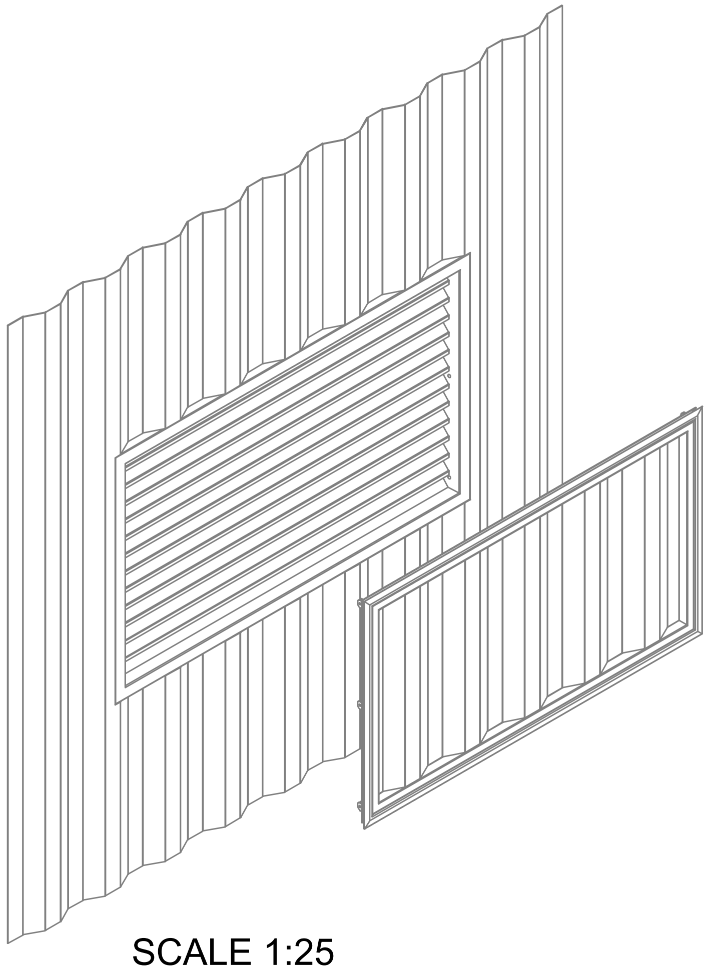

SCALE 1:25

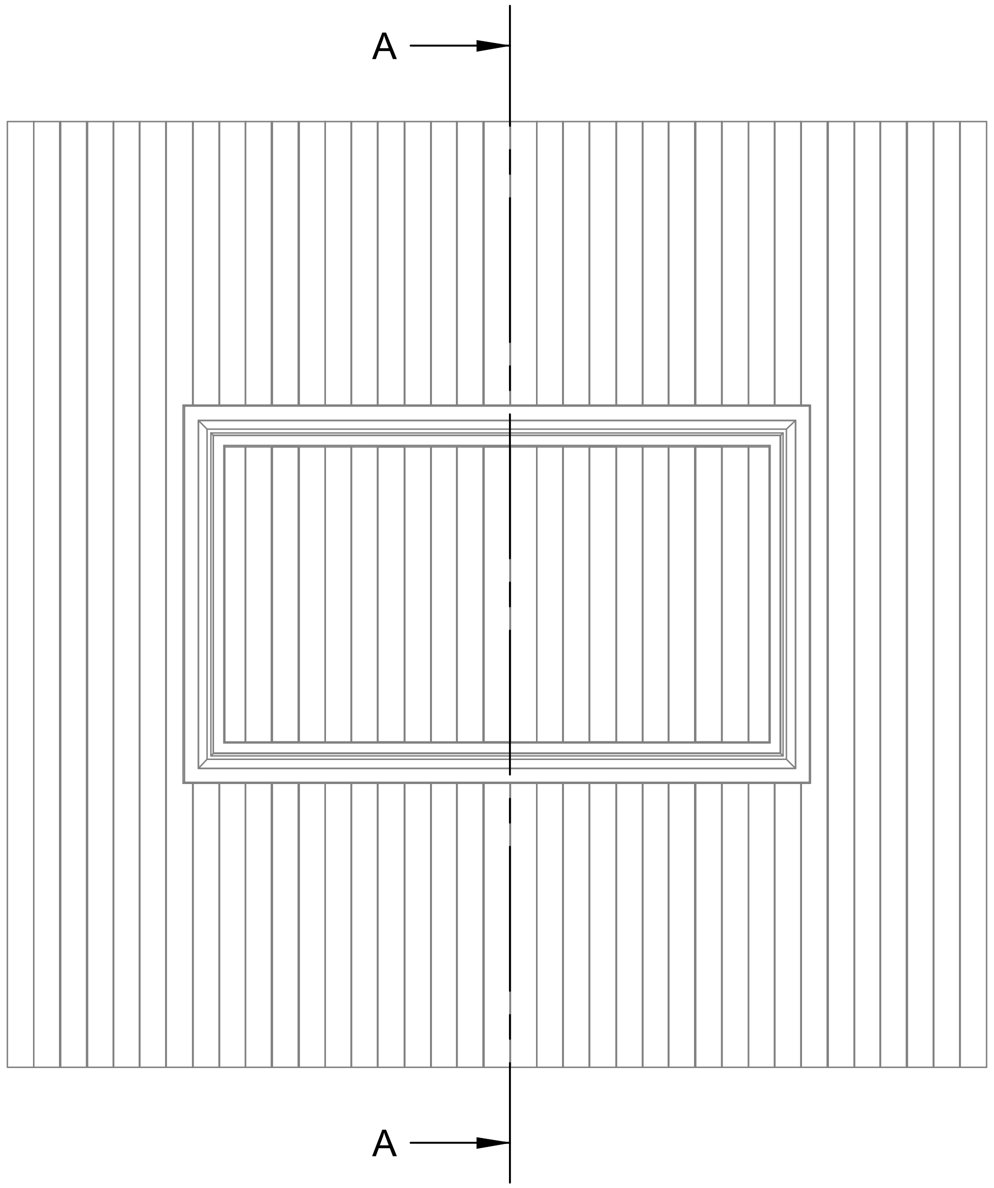

SCALE 1:25

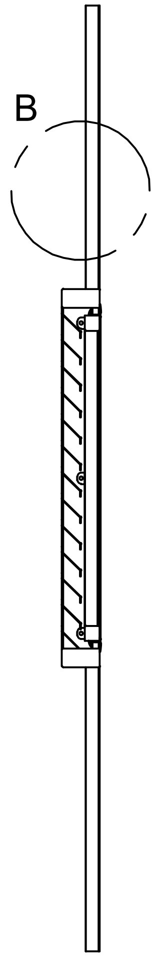

CUT A-A
SCALE 1:25

36-Grid Detail

transmissionzero

IMPERIAL

project	title		scale / drawing
Modular Portable Laboratory	TECHNICAL DESIGN PROJECT<br>GRID DETAIL	36	scale AS INDICATED<br>drawing 15549-DE-08

A3
420x297

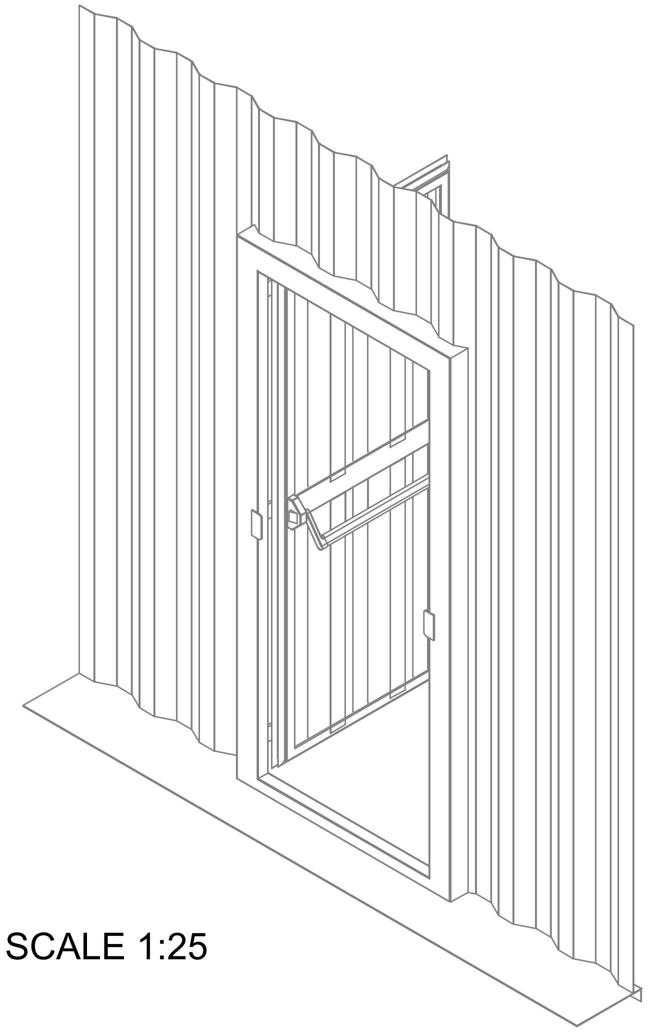

SCALE 1:25

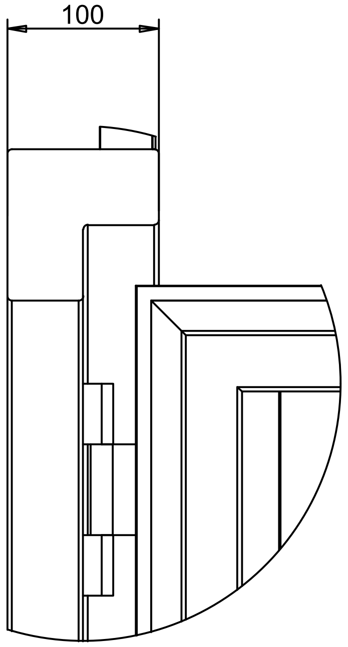

DETAIL B
SCALE 1:5

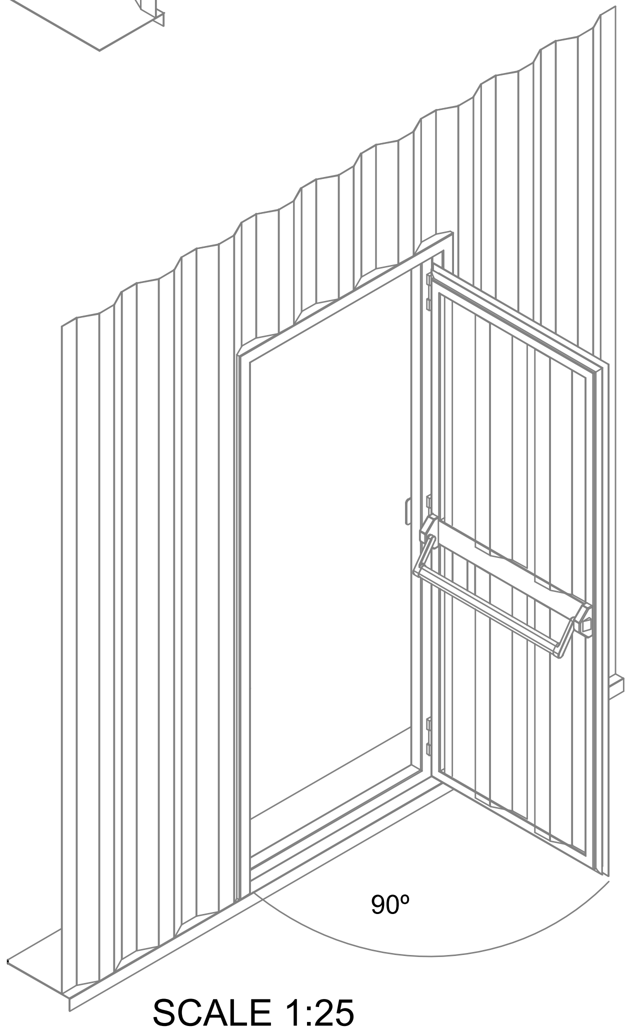

SCALE 1:25

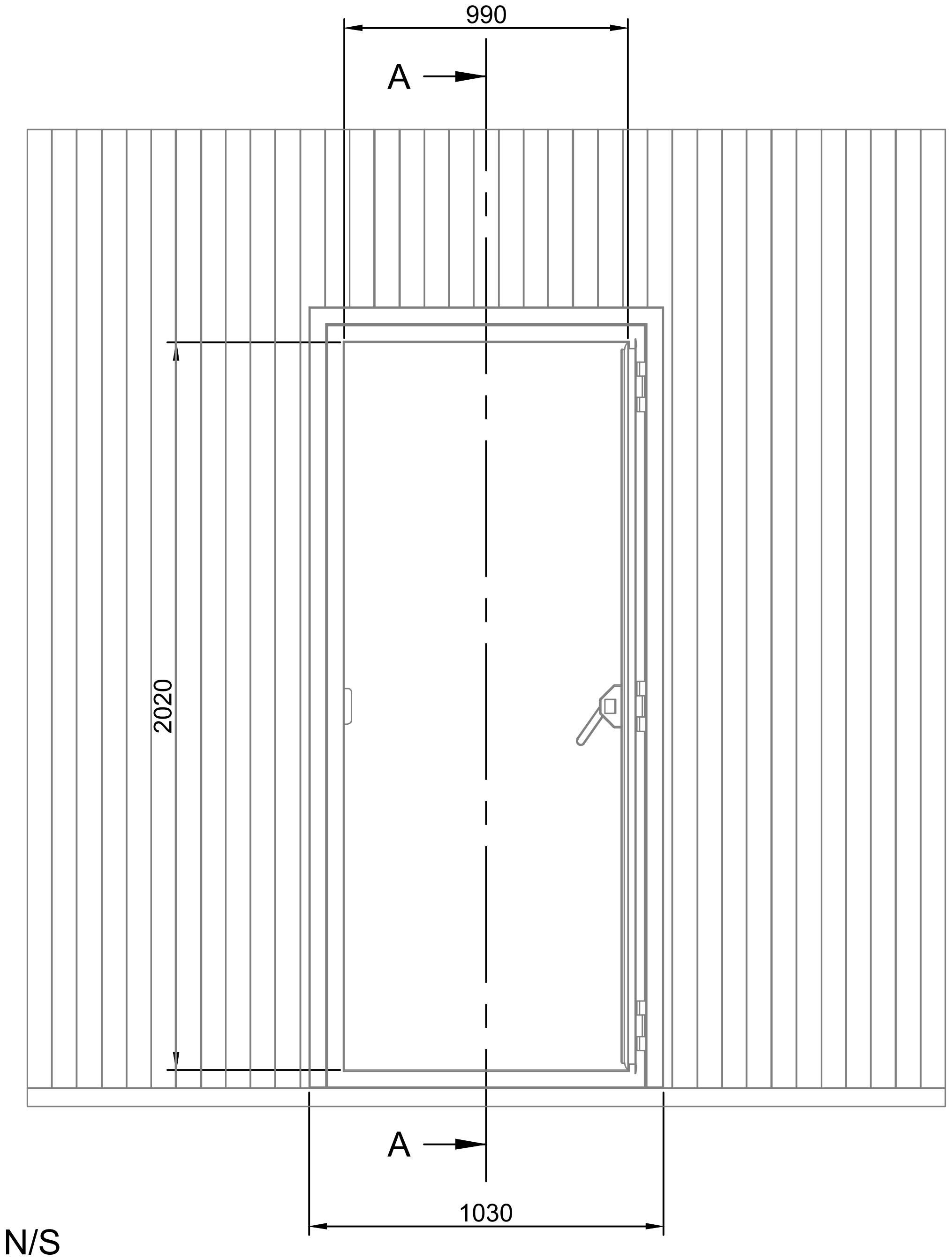

N/S

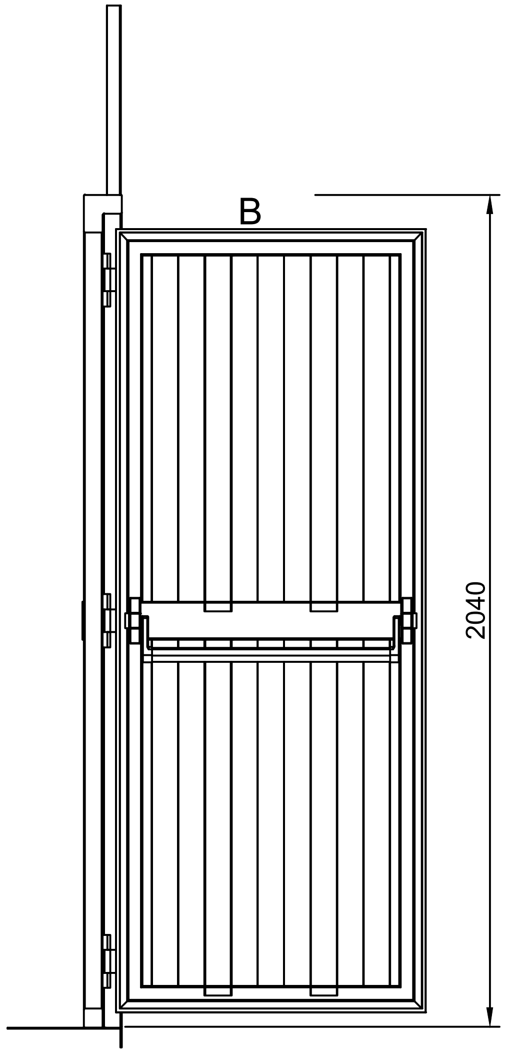

CUT A-A
N/S

37-Emergency Door Size

transmissionzero

IMPERIAL

project: Modular Portable Laboratory	TECHNICAL DESIGN PROJECT<br>title: EMERGENCY DOOR SIZE	37	scale: AS INDICATED	drawing: 15549-DE-09

A3
420x297

38-Laboratory Container Holes

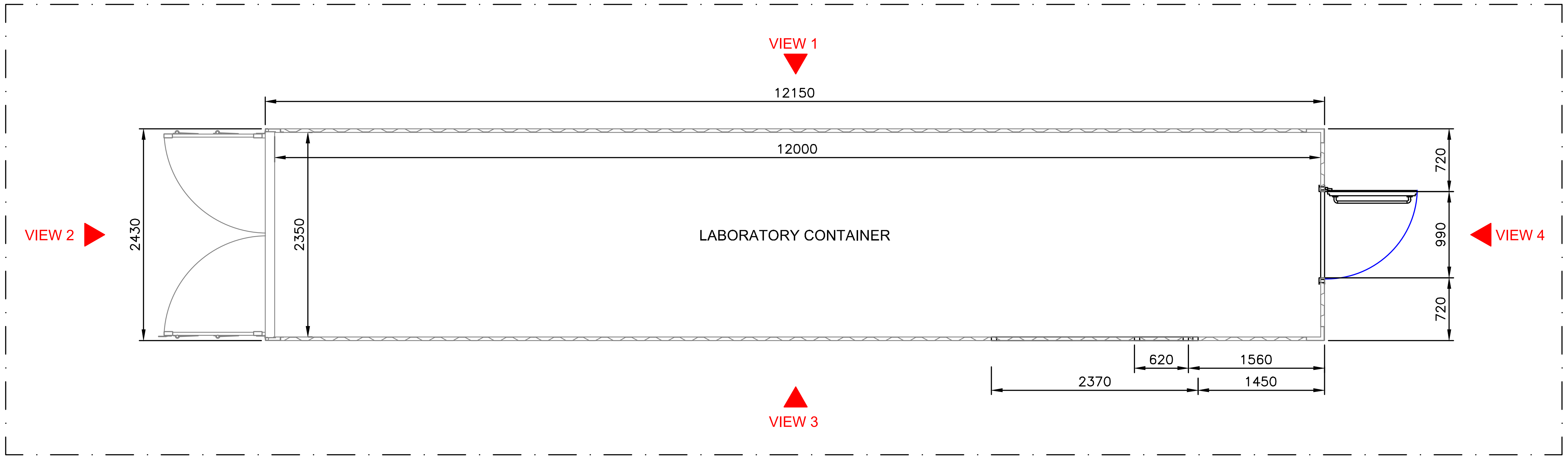

TOP VIEW

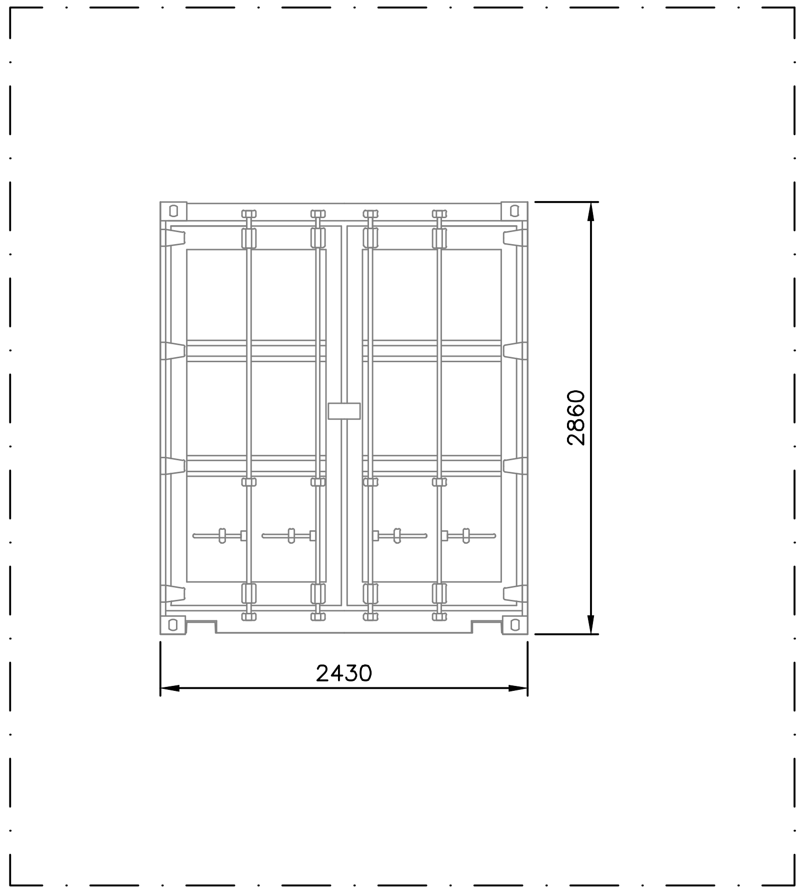

VIEW 2

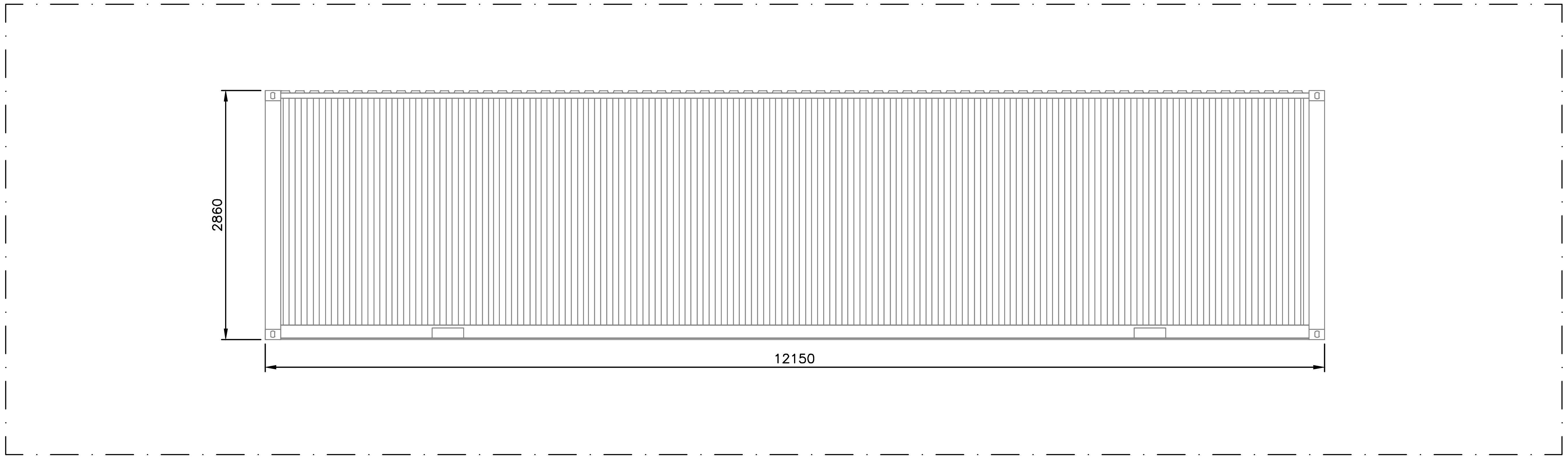

VIEW 1

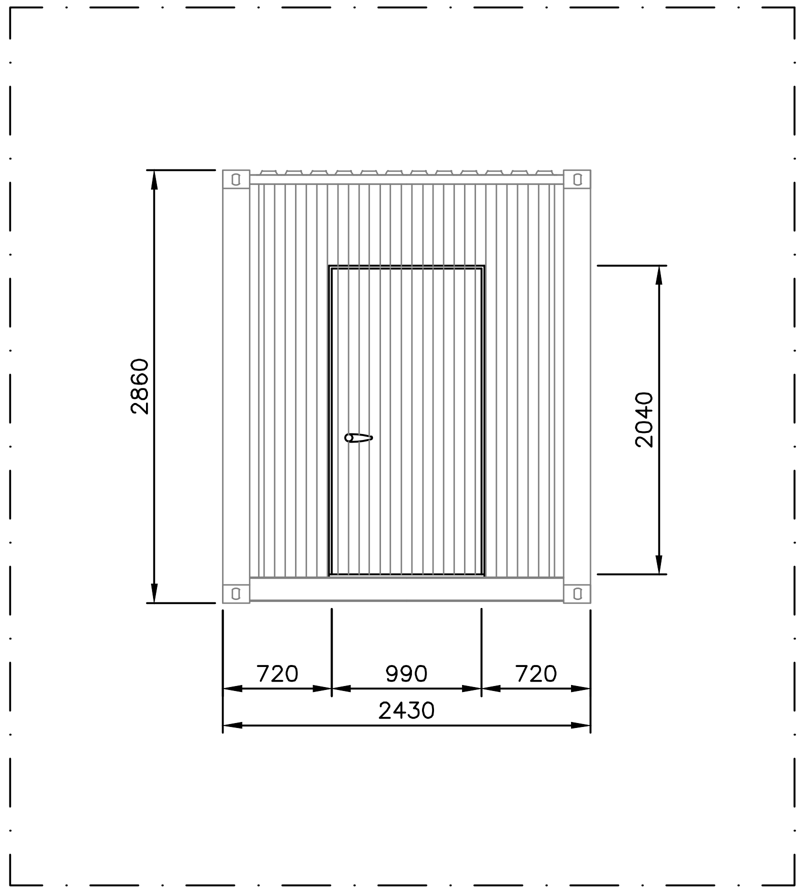

VIEW 4

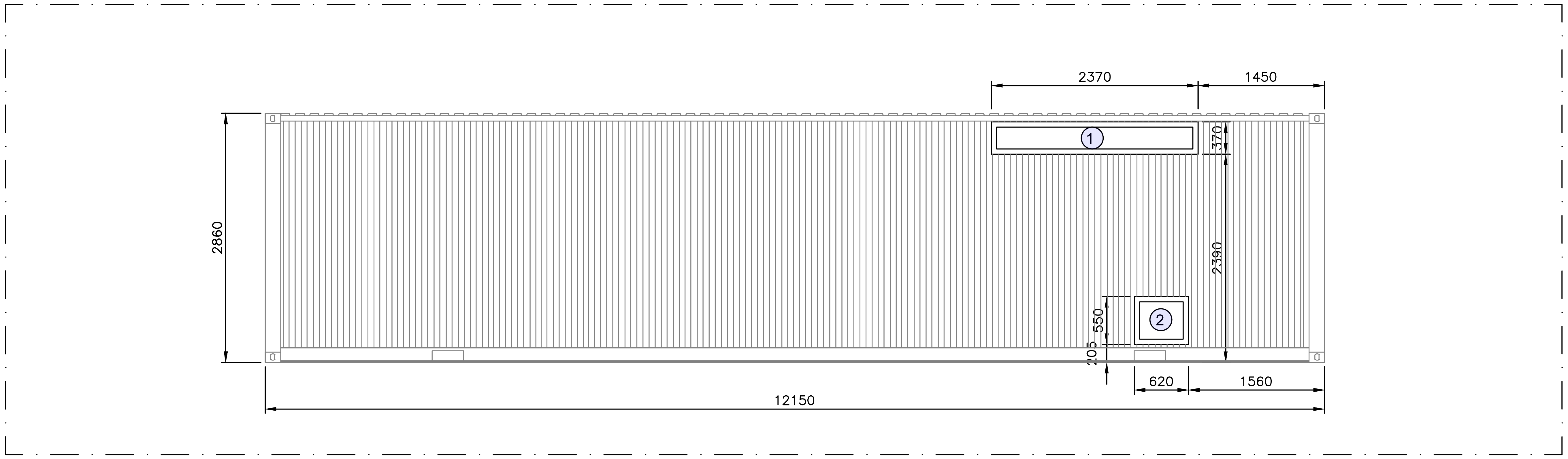

VIEW 3

NOTES: PAINT COLOR - RAL AGATE GREY 7038
INTERIOR FENCES OF THE HOLES ARE 60 mm.

transmission zero

IMPERIAL

project: Modular Portable Laboratory	title: TECHNICAL DESIGN PROJECT<br>LABORATORY CONTAINER HOLES	38	scale 1/50<br>drawing 15549-DE-10

A2
594x420

39-Secondary Installations Container Holes

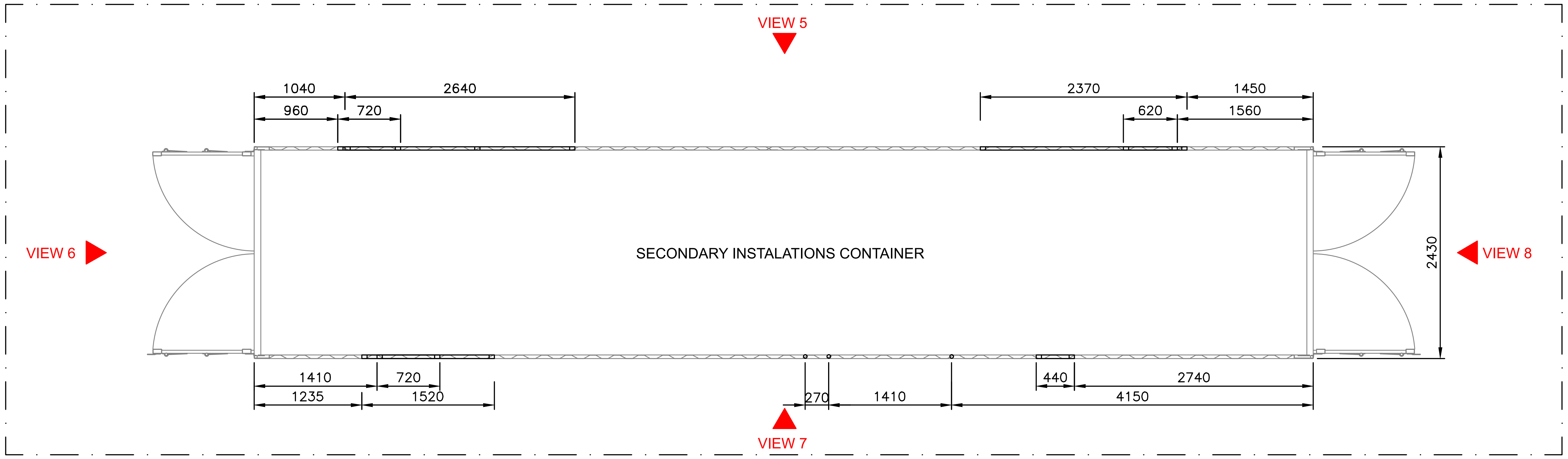

TOP VIEW

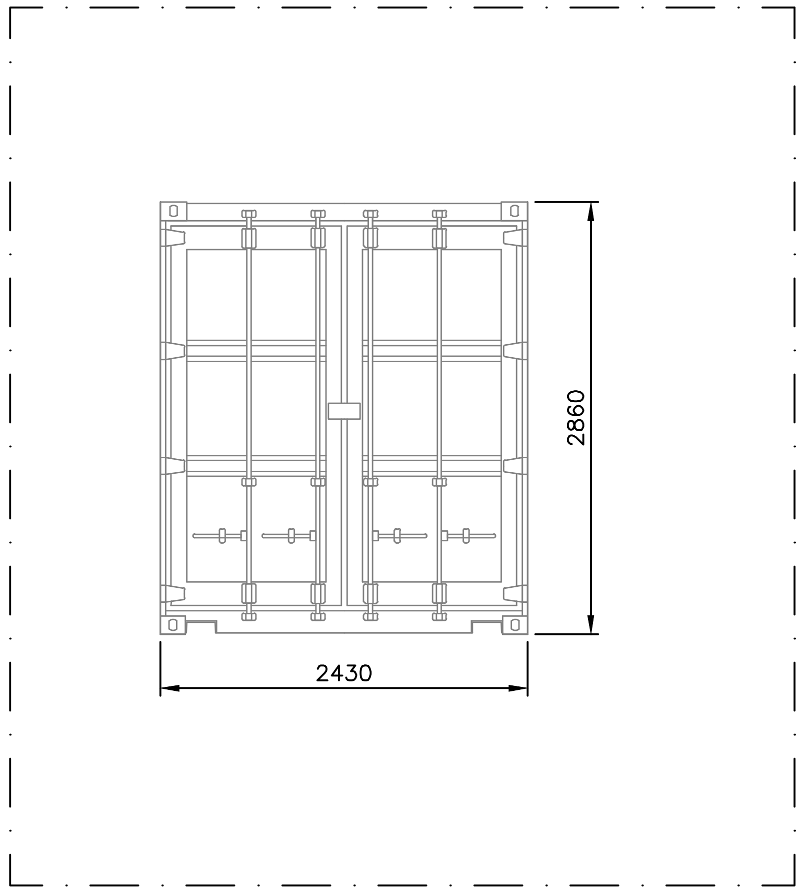

VIEW 6

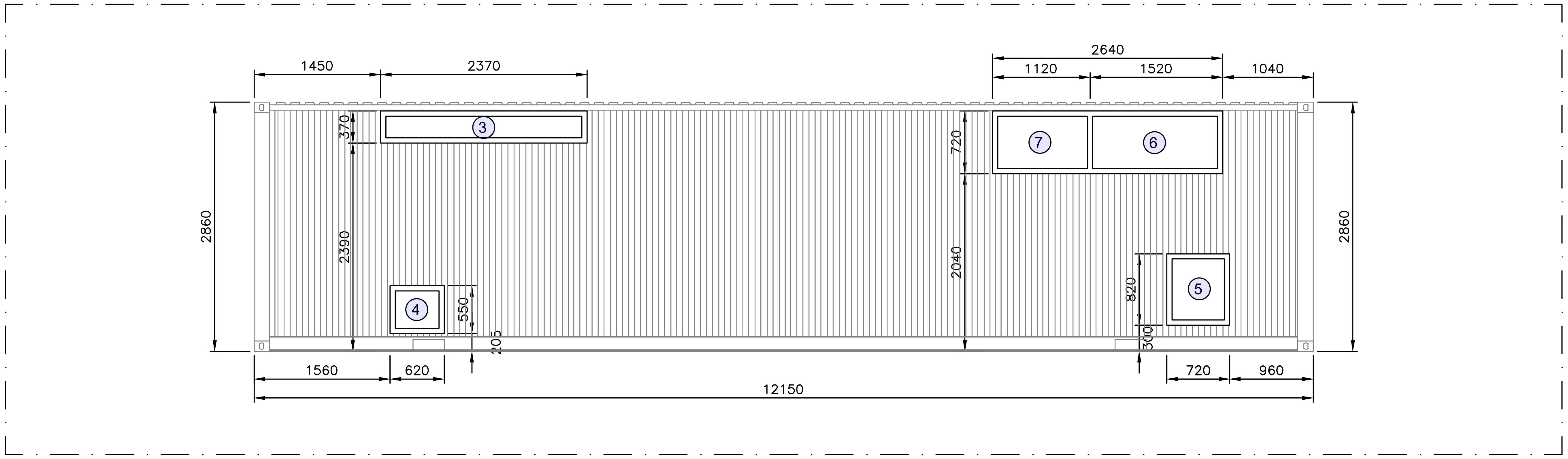

VIEW 5

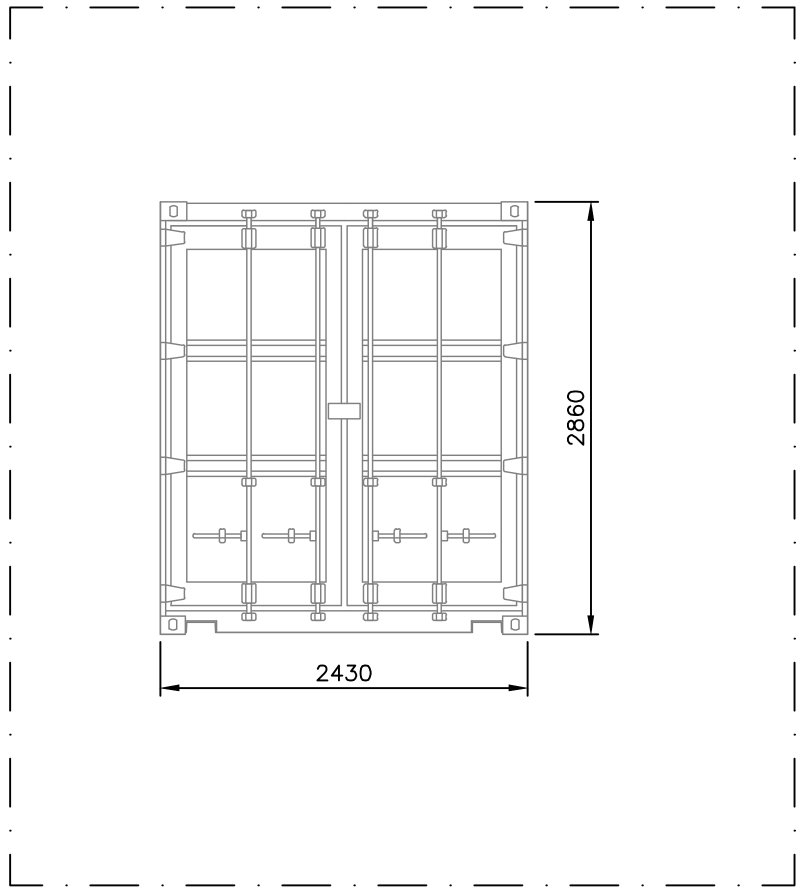

VIEW 8

NOTES: PAINT COLOR - RAL AGATE GREY 7038
INTERIOR FENCES OF THE HOLES ARE 60 mm.

10 11 12

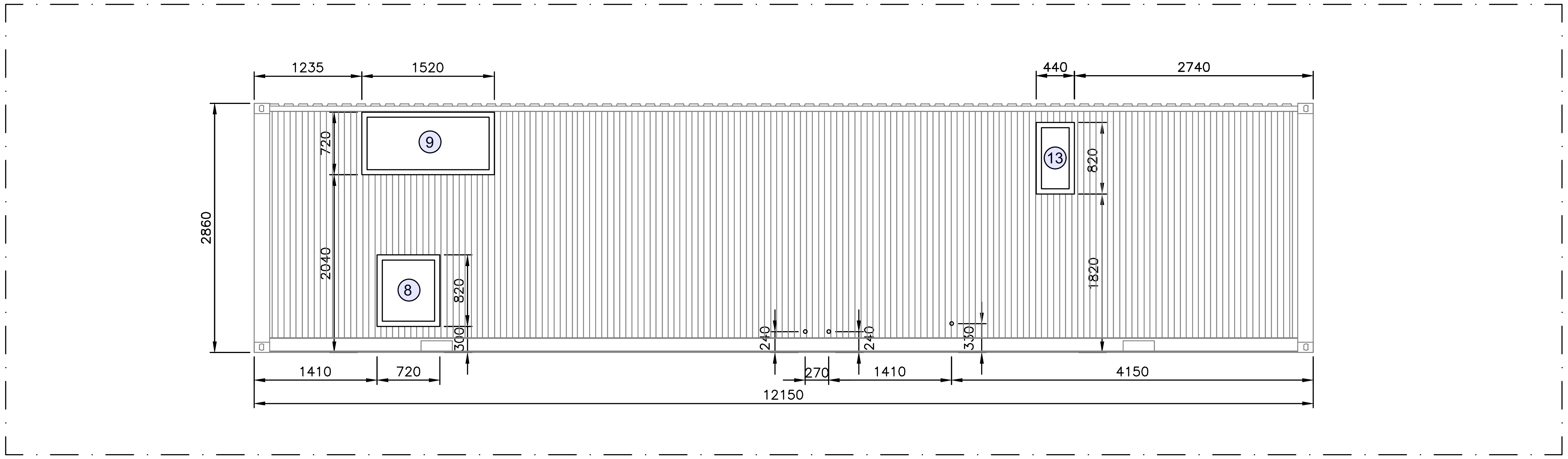

VIEW 7

transmissionzero

IMPERIAL

project: Modular Portable Laboratory	title: TECHNICAL DESIGN PROJECT SECONDARY INSTALATIONS CONTAINER. HOLES	39	scale: 1/50<br>drawing: 15549-DE-11

A2 594x420
