# Supplementary material for: Gene-drive-capable mosquitoes suppress patient-derived malaria in Tanzania
Source: Nature. 2025 Dec 10;649(8096):442–8. doi: 10.1038/s41586-025-09685-6 (PMC12779567; doi:10.1038/s41586-025-09685-6)
Supplement: Supplementary file 2 — Reporting Summary [file 41586_2025_9685_MOESM2_ESM.pdf]

nature portfolio

Corresponding author(s): George Christophides, Tibebu Habetwold, Dickson Lwetoijera, Nikolai Windbichler

Last updated by author(s): Sep 14, 2025

# Reporting Summary

Nature Portfolio wishes to improve the reproducibility of the work that we publish. This form provides structure for consistency and transparency in reporting. For further information on Nature Portfolio policies, see our Editorial Policies and the Editorial Policy Checklist.

## Statistics

For all statistical analyses, confirm that the following items are present in the figure legend, table legend, main text, or Methods section.

| n/a | Confirmed | |
|---|---|---|
| ☐ | ☒ | The exact sample size (*n*) for each experimental group/condition, given as a discrete number and unit of measurement |
| ☐ | ☒ | A statement on whether measurements were taken from distinct samples or whether the same sample was measured repeatedly |
| ☐ | ☒ | The statistical test(s) used AND whether they are one- or two-sided<br>*Only common tests should be described solely by name; describe more complex techniques in the Methods section.* |
| ☐ | ☒ | A description of all covariates tested |
| ☒ | ☐ | A description of any assumptions or corrections, such as tests of normality and adjustment for multiple comparisons |
| ☐ | ☐ | A full description of the statistical parameters including central tendency (e.g. means) or other basic estimates (e.g. regression coefficient) AND variation (e.g. standard deviation) or associated estimates of uncertainty (e.g. confidence intervals) |
| ☒ | ☐ | For null hypothesis testing, the test statistic (e.g. *F*, *t*, *r*) with confidence intervals, effect sizes, degrees of freedom and *P* value noted<br>*Give P values as exact values whenever suitable.* |
| ☒ | ☐ | For Bayesian analysis, information on the choice of priors and Markov chain Monte Carlo settings |
| ☐ | ☒ | For hierarchical and complex designs, identification of the appropriate level for tests and full reporting of outcomes |
| ☒ | ☐ | Estimates of effect sizes (e.g. Cohen's *d*, Pearson's *r*), indicating how they were calculated |

*Our web collection on statistics for biologists contains articles on many of the points above.*

## Software and code

Policy information about availability of computer code

| | |
|---|---|
| Data collection | *Provide a description of all commercial, open source and custom code used to collect the data in this study, specifying the version used OR state that no software was used.* |
| Data analysis | GraphPad Prism, R studio, ape package48 in RStudio |

For manuscripts utilizing custom algorithms or software that are central to the research but not yet described in published literature, software must be made available to editors and reviewers. We strongly encourage code deposition in a community repository (e.g. GitHub). See the Nature Portfolio guidelines for submitting code & software for further information.

## Data

Policy information about availability of data

All manuscripts must include a data availability statement. This statement should provide the following information, where applicable:

- Accession codes, unique identifiers, or web links for publicly available datasets
- A description of any restrictions on data availability
- For clinical datasets or third party data, please ensure that the statement adheres to our policy

All data supporting the findings of this study are available within the manuscript, its supplementary information files, and the source data provided. Raw sequencing data used to assess Plasmodium falciparum diversity have been deposited in the NCBI Sequence Read Archive under BioProject accession number PRJNA1299763. Source Data Sheets include: individual-level data on mosquito fecundity, fertility, and survival; raw parasitological survey data (parasitaemia and gametocytaemia)

from human participants; oocyst size measurements and corresponding mosquito genotypes; qPCR data on parasite presence in mosquito midguts and salivary glands. There are no restrictions on data availability. All materials and datasets can be accessed without limitations, and no third-party data were used that require separate permission.

## Research involving human participants, their data, or biological material

Policy information about studies with human participants or human data. See also policy information about sex, gender (identity/presentation), and sexual orientation and race, ethnicity and racism.

| | |
|---|---|
| Reporting on sex and gender | Participants were male and female children aged 6-14 years. Sex was not used as a stratification variable, and analyses were not disaggregated by sex or gender, as the primary objective was to collect gametocyte-positive blood for mosquito infection assays. Sex-disaggregated data were not expected to impact the study outcomes, which focused on parasite transmission potential rather than clinical or epidemiological differences between sexes. |
| Reporting on race, ethnicity, or other socially relevant groupings | All participants were recruited from rural communities in the Pwani region of Tanzania. While ethnicity or socio-cultural groupings were not specifically recorded, the study population represents local school-attending children in endemic rural Tanzanian settings. These social identifiers were not relevant to the scientific objectives, which were focused on Plasmodium falciparum diversity and mosquito transmission dynamics. |
| Population characteristics | The study population consisted of primary and lower-secondary schoolchildren (ages 6–14) residing in three rural villages (Wami Mkoko, Miono Kikalo, and Kibindu) in Pwani Region, Tanzania. Participants were screened for malaria parasitaemia and gametocytaemia using RDTs and microscopy. Children with high gametocyte densities were invited to provide blood samples for membrane feeding assays. |
| Recruitment | Participants were recruited via school-based malaria surveys in collaboration with school staff, healthcare workers, and community engagement teams. Inclusion criteria were age (6-14) and willingness to participate, with parental/guardian consent and child assent. Children with high gametocyte levels were invited for blood donation. Recruitment was therefore opportunistic and based on gametocyte carriage. There is potential for self-selection bias based on parental willingness, though this is unlikely to have influenced parasite diversity or mosquito infection outcomes. |
| Ethics oversight | All study protocols involving human participants were reviewed and approved by the Ifakara Health Institute Institutional Review Board (IHI-IRB) and the National Institute for Medical Research (NIMR), Tanzania. Informed written consent was obtained from parents or guardians, and oral assent was obtained from the children, in accordance with ethical guidelines for research involving minors. Community engagement activities accompanied all human participant research to ensure transparency, understanding, and trust. |

Note that full information on the approval of the study protocol must also be provided in the manuscript.

# Field-specific reporting

Please select the one below that is the best fit for your research. If you are not sure, read the appropriate sections before making your selection.

☒ Life sciences ☐ Behavioural & social sciences ☐ Ecological, evolutionary & environmental sciences

For a reference copy of the document with all sections, see nature.com/documents/nr-reporting-summary-flat.pdf

# Life sciences study design

All studies must disclose on these points even when the disclosure is negative.

| | |
|---|---|
| Sample size | No formal statistical power calculations were performed. Sample sizes were chosen based on prior similar experiments and logistical feasibility, ensuring sufficient biological replicates for statistical comparisons (e.g. mosquito fertility and fecundity, infection prevalence and intensity, oocyst size). |
| Data exclusions | Some mosquito samples were excluded when DNA could not be isolated, genotyping failed or insufficient numbers were available for analysis. No other data exclusion was done. |
| Replication | All quantitative experiments were replicated independently with consistent results. |
| Randomization | Randomisation was not applicable for mosquito line comparisons, which were grouped by line/genotype. Human blood samples were used opportunistically based on gametocytaemia levels, not randomised. |
| Blinding | Blinding in most experiments was not possible due to fluorescent markers or genotyping required for identification of transgenic mosquitoes. Genotyping was used post hoc to confirm genotype. Blinding was used in most cases when confirming and quantifying mosquito infection phenotypes. |

# Reporting for specific materials, systems and methods

We require information from authors about some types of materials, experimental systems and methods used in many studies. Here, indicate whether each material, system or method listed is relevant to your study. If you are not sure if a list item applies to your research, read the appropriate section before selecting a response.

## Materials & experimental systems

| n/a | Involved in the study |
|---|---|
| ☒ | ☐ Antibodies |
| ☒ | ☐ Eukaryotic cell lines |
| ☒ | ☐ Palaeontology and archaeology |
| ☒ | ☐ Animals and other organisms |
| ☐ | ☒ Clinical data |
| ☒ | ☐ Dual use research of concern |
| ☒ | ☐ Plants |

## Methods

| n/a | Involved in the study |
|---|---|
| ☒ | ☐ ChIP-seq |
| ☒ | ☐ Flow cytometry |
| ☒ | ☐ MRI-based neuroimaging |

# Clinical data

Policy information about clinical studies

All manuscripts should comply with the ICMJE guidelines for publication of clinical research and a completed CONSORT checklist must be included with all submissions.

| | |
|---|---|
| Clinical trial registration | n/a |
| Study protocol | n/a |
| Data collection | n/a |
| Outcomes | n/a |

# Plants

| | |
|---|---|
| Seed stocks | n/a |
| Novel plant genotypes | n/a |
| Authentication | n/a |
